# Interactions of antiparasitic sterols with sterol 14α-demethylase (CYP51) of human pathogens

**DOI:** 10.1186/2193-1801-3-679

**Published:** 2014-11-20

**Authors:** Jasmine Warfield, William N Setzer, Ifedayo Victor Ogungbe

**Affiliations:** Department of Chemistry and Biochemistry, Jackson State University, Jackson, MS 39217 USA; Department of Chemistry, University of Alabama in Huntsville, Huntsville, AL 35899 USA

**Keywords:** Protozoa, Docking, Sterol 14α-demethylase, Sterols, Antiparasitic

## Abstract

Sterol 14α-demethylase is a validated and an attractive drug target in human protozoan parasites. Pharmacological inactivation of this important enzyme has proven very effective against fungal infections, and it is a target that is being exploited for new antitrypanosomal and antileishmanial chemotherapy. We have used *in silico* calculations to identify previously reported antiparasitic sterol-like compounds and their *structural congeners* that have preferential and high docking affinity for CYP51. The sterol 14α-demethylase from *Trypanosoma cruzi* and *Leishmania infantum,* in particular, preferentially dock to taraxerol, *epi*-oleanolic acid, and α/β-amyrim structural scaffolds. These structural information and predicted interactions can be exploited for fragment/structure-based antiprotozoal drug design.

**Electronic supplementary material:**

The online version of this article (doi:10.1186/2193-1801-3-679) contains supplementary material, which is available to authorized users.

## Introduction

**Figure 1:**
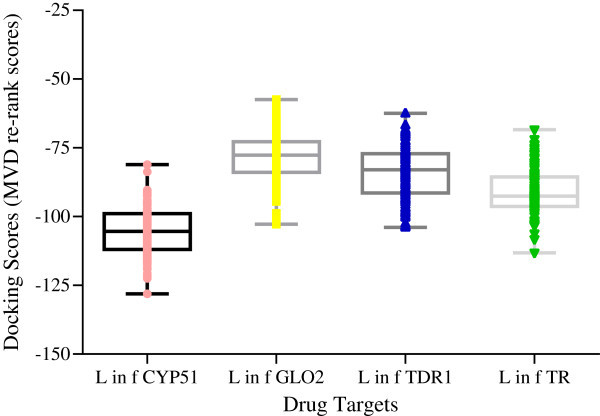
**Docking energies (re-rank score) of antiparasitic sterols in L. infantum drug targets (Sterol 14** **α** **demethylase (CYP51), glyoxalase II (GLO2), thiol-dependent reductase I (TDR1), trypanothione reductase (TR).** The figure shows the selectivity of sterol-like compounds for CYP51. One—way ANOVA followed by Tukey’s multiple comparison test suggest statistically significant difference between the means of all groups (P < 0.0001).

Sterol 14α-demethylase (CYP51), a cytochrome P450 mixed-function oxidase, is involved in the biosynthesis of numerous structurally similar sterols in animals, fungi, plants and protozoans. Several of these sterols serve as important component of cell membranes, and they are vital to the maintenance of cellular structural integrity in those organisms. Some derivatives of plant sterols also function as signaling molecules, and as antifungal agents (Rozhon et al. 2013; Geisler et al. 2013; Qi et al. 2006). CYP51 is a well-known druggable target in fungi. Several generations of azole-based CYP51 inhibitors have already been developed as clinical antifungal drugs (Peng et al. 2013). The specificity of these azole-based drugs for fungal CYP51 has been linked to the low sequence identity between the fungal enzyme and mammalian CYP51 (Aoyama 2005). The sterol 14α-demethylases in *Trypanosoma cruzi*, *Trypanosoma brucei*, *Leishmania infantum* and *Mycobacterium tuberculosis* have also been studied as potential targets for drug discovery and development. Although the apparent lack of *de novo* sterol biosynthesis in *M. tuberculosis*, and the fact that other mycobacteria cytochrome P450s like CYP121 are more sensitive to azole drugs makes CYP51 a less desirable drug target in that organism (Cole et al. 1998; McLean et al. 2002). Protozoans, on the other hand, especially *Trypanosoma cruzi* and *Leishmania infantum,* have been widely reported to be susceptible to chemotypes targeting CYP51 (Urbina et al. 1996; Buckner et al. 2012; Lorente et al. 2004; Doyle et al. 2010; Gunatilleke et al. 2012). Drug discovery targeting CYP51 remains one of the active areas of antitrypanosomal research. Identifying chemotypes via *in-silico* screening and subsequent phenotype-based in-vitro validation has become the bedrock of modern small molecule drug discovery. Our previous works involving virtual screening of antitrypanosomal natural products libraries against protozoan drug targets revealed that antitrypanosomal sterol–type compounds have high affinity for CYP51 as illustrated with a few drug targets in Figure 1 (Ogungbe and Setzer 2013; Setzer and Ogungbe 2012). It can be speculated that the predicted affinities is because of the structural compatibility between the sterol-like compounds and CYP51. The objective of this work is to identify antiparasitic compounds and their structural congeners that display selectivity for protozoal sterol 14α-demethylase, *in silico,* and on which structure- or shape-based analogues or potential drug leads can be designed or identified.

## Material and methods

### Compound and protein structure preparation

Ligands used for this study were geometry optimized using the molecular mechanics force field (MMFF) algorithm in Spartan’10 for Windows ( 2011). The docking studies were carried out using the crystal structures of *T. cruzi* CYP51 (PDB Id: 3khm, 3zg2, 4h6O (Lepesheva et al. 2010; Hargrove et al. 2013; Andriani et al. 2013)), *T. brucei* CYP51 (PDB Id: 3g1q, 3gw9, 4g3j ( Hargrove et al)), *M. tuberculosis* CYP51 (PBD Id: 2ci0, 2w0a (Chen et al. 2009; Podust et al. 2007)), *L. infantum* CYP51 (PBD Id: 3l4d (Hargrove et al. 2011)), and *H. sapiens* CYP51 (3jus, 3juv, 3ld6 (Strushkevich and Usanov 2014; Strushkevich et al. 2010)) from the RCSB Protein Data Bank. The protein structures were used as rigid model structures, no relaxation was performed and assignments of ionic charges on each protein structure were based on standard protonation states and the default templates of Molegro Virtual Docker ( 2011b; Thomsen and Christensen 2006). The similarity search was carried out using the default sub-structure similarity search engine of the Dictionary of Natural Products ( 2013). Statisitical comparison was carried using GraphPad Prism.

### Docking simulation and scoring

Flexible ligand models were used for docking and post-docking geometry optimizations. The post-docking geometry optimizations were carried out using the Nelder-Mead Simplex method in Molegro Virtual Docker. Simulations were carried out using the substrate binding site of CYP51. A docking sphere (15 Å radius) was placed on the binding sites of each protein structure in order to allow different orientations of each ligand to be searched in the binding cavities and for multiple protein-ligand poses to be returned. The RMSD threshold for multiple cluster poses was set at <1.00 Å. The docking algorithm was set at maximum iterations of 1500 with a simplex evolution population size of 50 and a minimum of 30 runs for each ligand. Each binding site of oligomeric structures was searched, and the lowest energy pose (based on the re-rank scores) for each ligand across all protein structures are presented in Additional file 1: Table S1–S5. The docking scores of known CYP51 inhibitors *N*-1-(2,4-dichlorophenyl)-2-(1H-imidazol-1-yl)ethyl)-4-(5-phenyl-1,3,4-oxadi-azol-2-yl)benzamide and ketoconazole are presented in Additional file 1: Table S6, and PDB files of CYP51-ligand complexes discussed in this article are provided as Additional file 2 in the supporting information.

## Results and discussion

### Antitrypanosomal sterols have high affinity for *H. sapiens*CYP51 *in silico*

**Figure 2:**
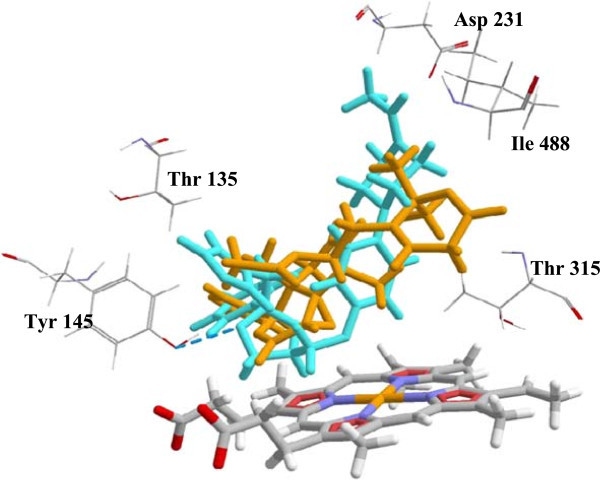
**The lowest energy docking poses of carapolide A (Orange) and ketoconazole (Blue) in the substrate binding site of human CYP51.** Hydrogen bonding interaction between ketoconazole and Tyr 145 is depicted with the blue dash lines. Both compounds were predicted to interact with the amino acid residues shown. The molecular surface diagram is shown in Additional file 1: Figure S2.

From our previous work on *T. brucei* and *L. infantum* drug targets (Ogungbe and Setzer 2013; Setzer and Ogungbe 2012), we proposed that structural compatibility dictates the molecular recognition between antitrypanosomal sterol–type compounds and CYP51. The first aim of this work was to identify compounds that are selective for trypanosomal CYP51s *in silico*. This was accomplished via a comparative docking analysis involving 137 sterol-like antiparasitic natural products and the sterol 14α-demethylase from *T. brucei, T. cruzi*, *M. tuberculosis*, *L. infantum* and *H. sapiens.* The result of the simulations is presented in Additional file 1: Figure 1 and Table S1. The results show that about 50 percent of the antitrypanosomal compounds preferentially dock to the human CYP51. The top-five compounds for *H. sapiens* CYP51 are 3-acetylkhayalactone, carapolide A, gedunin, grandifolide A and swietenine (Docking energies are -142.12, -130.87, -128.70, -128.36 and -128.02 kJ/mol, respectively). 3-Acetylkhayalactone was predicted to bind favourably in a 157.70 Å^3^ cavity distal from the substrate binding site. The other four compounds were predicted to bind at the substrate binding site. Visual inspection reveals that they overlap with the co-crystallized ligand ketoconazole. The strongest docking poses of carapolide A and ketoconazole are shown in Figure 2. Some of the target residues for the limonoid carapolide A are Ala 311, Gly 307, Ile 377 and 488, Tyr 131 and 145, Thr 135, 315 and 318, and Phe 234. Most of those residues have been reported to participate in the interactions between human CYP51 and antifungal azoles (Strushkevich et al. 2010).

### Substructure similarity search for selective sterols

**Figure 3:**
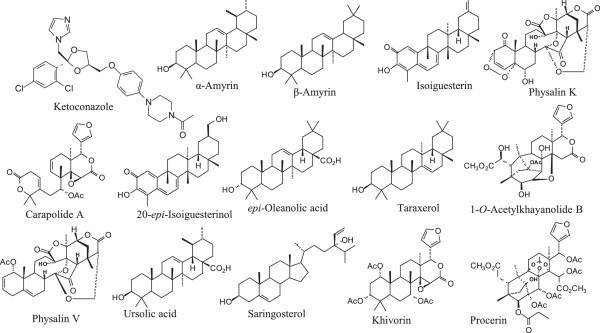
**Structures of antiparasitic sterol-like compounds that show preferential docking to** ***T. cruzi*** **-,** ***T. brucei*** **-,** ***L. infantum*** **-,** **and** ***M. tuberculosis*** **CYP51.**

A number of compounds studied were selective for *T. cruzi* CYP51. These include 20-*epi*-isoiguesterinol, isoiguesterin, khivorin, procerin, taraxerol, ursolic acid as well as α- and β-amyrin. Physalin K and procerin were selective for *T. brucei* CYP51. 7-Deacetylkhivorin was selective for *M. tuberculosis* CYP51 while 1-*O*-acetylkhayanolide B, physalin V, saringosterol and *epi*-oleanolic acid were selective for *L. infantum* CYP51 (Figure 3 and Table 1). A substructure similarity search was carried out for compounds that displayed selectivity towards CYP51 from *T. brucei, T. cruzi*, *and L. infantum*. This was carried out in order to identify sterol-like natural products that have strong docking affinities for CYP51s, and that can be evaluated as potential CYP51 inhibitors. We used the Dictionary of Natural Products DNP ( 2013) as a reference for those structures. From the similarity search, nine compounds were found to be structurally-related to 20-*epi*-isoiguesterinol and isoiguesterin. Out of the nine, eight are isomers of celastrol. The other compound was excelsine. The docking re-rank scores of those compounds, however, were less or about the same as those of 20-*epi*-isoiguesterinol and isoiguesterin for *T. cruzi* CYP51 (Additional file 1: Table S2).

**Table 1:** **Docking scores of selective antiparasitic agents for CYP51**

Antitrypanosomal agents	CYP51				
	***T. cruzi***	***T. brucei***	***M. Tuberculosis***	***L. infantum***	***H. sapiens***
1-*O*-Acetylkhayanolide B	-102.7	-106.1	-101.2	**-107.0**	-96.5
20-*epi*-Isoiguesterinol	-**101.2**	-91.9	-79.2	-34.7	-88.5
**7-Deacetylkhivorin**	-101.8	-70.0	**-114.1**	-88.2	-44.6
**Khivorin**	-**111.7**	-97.5	-101.9	-94.5	-96.6
**Physalin K**	-6.4	**-112.1**	-84.6	-94.0	-88.7
**Physalin V**	-104.3	-97.9	-86.3	**-114.6**	-77.4
**Procerin**	128.0	**-121.4**	-85.4	-48.6	-49.3
**Saringosterol**	-112.3	-102.5	-94.7	**-122.0**	-106.6
**Taraxerol**	**-104.7**	-74.8	-46.7	-89.5	-88.1
**Ursolic acid**	**-110.9**	-79.5	-82.7	-102.3	-90.2
**α-Amyrin**	**-102.4**	-80.2	2.5	-99.4	-88.8
**β-Amyrin**	**-104.8**	-79.5	5.5	-99.0	-78.5
***epi*** **-Oleanolic acid**	-37.0	-95.5	-88.2	**-105.7**	-85.8
**Isoiguesterin**	**-94.0**	-85.5	-70.9	-83.7	-78.9

The bold numbers indicates the lowest docking score for each compound.

**Figure 4:**
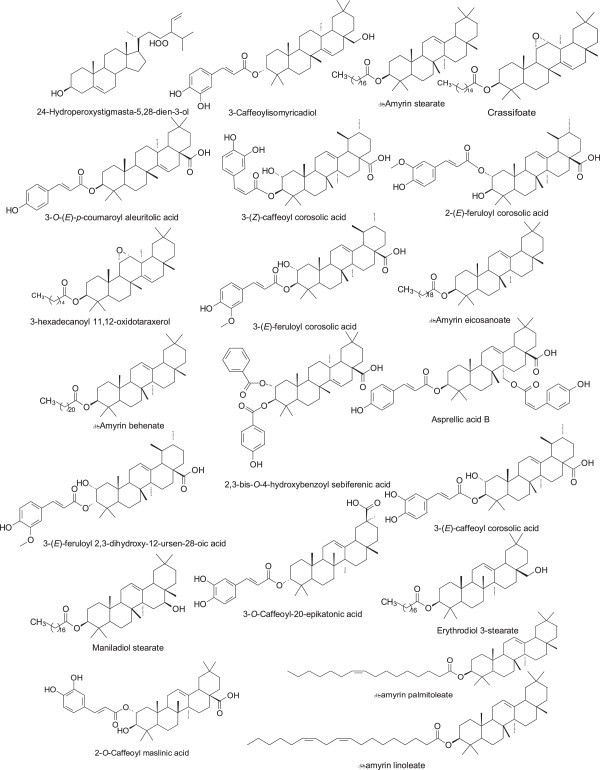
**Structures of sterol derivatives that were selective for** ***T. cruzi*** **and** ***L. infantum*** **CYP51.**

In the case of khivorin, 7-deacetylkhivorin, physalin K and V, and 1-*O*-acetylkhayanolide B, all the structurally- related compounds found in the DNP were part of our original compound dataset while the structurally complex limonoid procerin lacks any structural congener. Seventy-nine and 304 compounds were found to share substructural similarity with taraxerol and ursolic acid, respectively. Several of the 79 taraxerol structural congeners have high docking affinity for *T. cruzi* CYP51. These include the hexadecanoyl derivative of 11,12-oxidotaraxerol, the 2,3-bis-(4-hydroxybenzoyl) derivative of sebiferenic acid, the 3-*O*-(*E*)-*p*-coumarate derivative of aleuritolic acid in addition to crassifoate and 3-caffeoylisomyricadiol (Figure 4 and Additional file 1: Table S3). The structural congeners of ursolic acid that have high docking affinity for *T. cruzi* CYP51 are the 3-(*E*)-feruloyl derivative of 2,3-dihydroxy-12-ursen-28-oic acid, 3-(*Z*)-feruloyl corosolic acid, 3-(*E*)-caffeoyl corosolic acid, 3-(*Z*)-caffeoyl corosolic acid and 2-(*E*)-feruloyl corosolic (Figure 4 and Additional file 1: Table S4). In the case of *epi*-oleanolic acid, α-amyrin, and β-amyrin, 1213 structural congeners were obtained and those compounds were used for docking simulations with *T. cruzi* CYP51 and *L. infantum* CYP51. Our result shows that β-amyrin eicosanoate, β-amyrin stearate and β-amyrin behenate as well as, β-amyrin linoleate and β-amyrin-palmitoleate have the top docking affinities for *T. cruzi* CYP51 (Figure 4 and Additional file 1: Table S5). For *L. infantum* CYP51; maniladiol stearate, erythrodiol 3-stearate, asprellic acid B, the 2-*O*-caffeate derivative of maslinic acid, and the 3-*O*-caffeate derivative of 20-epikatonic acid have the top docking affinities (Figure 4 and Additional file 1: Table S5).

### Taraxerol structural congeners and *T. cruzi*CYP51

The triterpene taraxerol has been reported to have moderate antitrypanosomal and antiplasmodial activity (IC_50_ = 10.5 μM for *T. brucei rhodesiense* and 8.5 μM for *Plasmodium falciparum*) (Gachet et al. 2011). We were unable to find any record of its biological activity against *T. cruzi* in the literature. The top structural congeners of taraxerol from the calculations are 3-hexadecanoyl 11,12-oxidotaraxerol and crassifoate. These compounds were isolated from the leaves of *Erythroxylum passerinum* and *Nepeta crassifolia*, respectively (Barreiros et al. 2002; Ibrahim and Ali 2007). The high docking energies of these compounds for *T. cruzi* CYP51 can be attributed to their relative bulkiness and shape. The orientation of the compounds in the substrate binding site of *T. cruzi* CYP51 is also atypical of sterols. Their long hydrophobic lipid moieties are in close proximity with the heme cofactor, while their triterpene-structural moieties occupies the hydrophobic access channel of the binding site. This is similar to what has been reported for the extended arm of posaconazole (Lepesheva et al. 2010) although structural model of CYP51 co-crystallized with fatty acid ester of any sterol is yet to be reported. It would be of particular importance to determine the orientation(s) of fatty acid esters of sterols in the CYP51 binding cavity experimentally. Nevertheless, the shape that the two compounds adopt in the theoretical models can be used to design similar-shaped compounds that possess heme inactivating functional groups in addition to elongated hydrophobic moieties like in the case of posaconazole.

**Figure 5:**
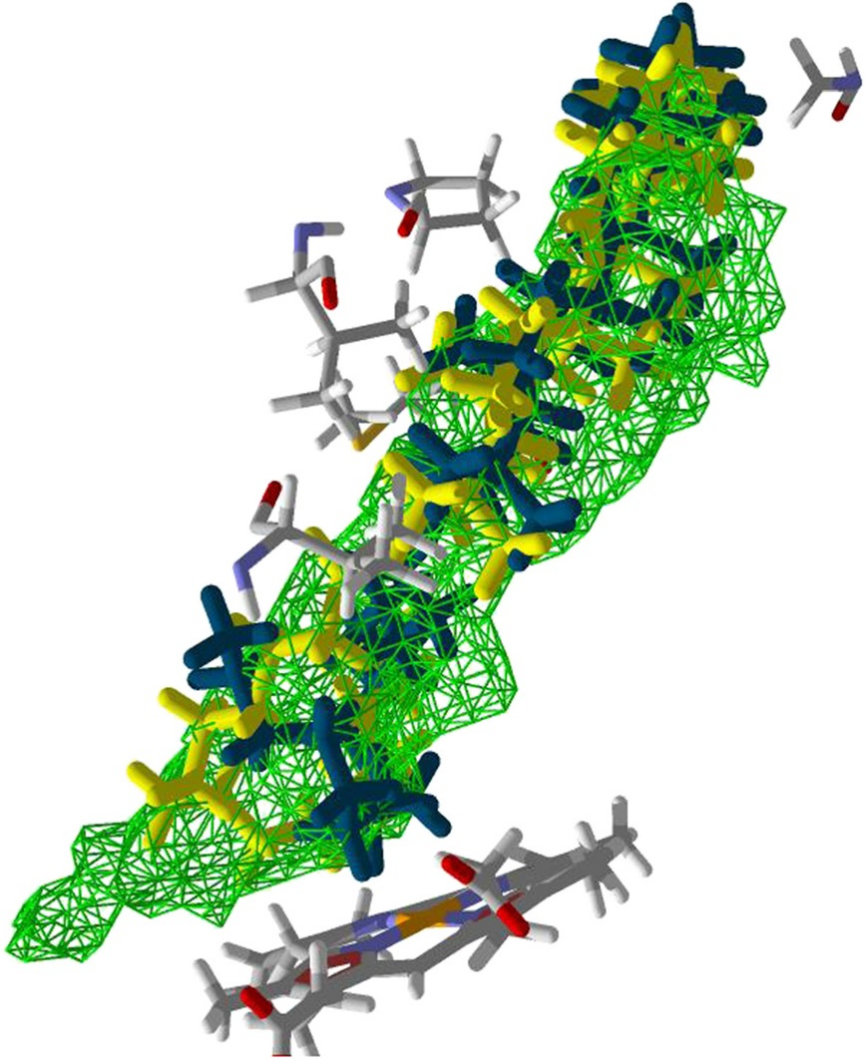
**The lowest energy pose of 3-hexadecanoyl 11,12-oxidotaraxerol (yellow) and crassifoate (blue) in the active site of** ***T. cruzi*** **CYP51.** The hydrophobic amino acid residues in the active site offer a favorable binding patch for the esterified sterols. The molecular surface diagram is shown in Additional file 1: Figure S3.

**Figure 6:**
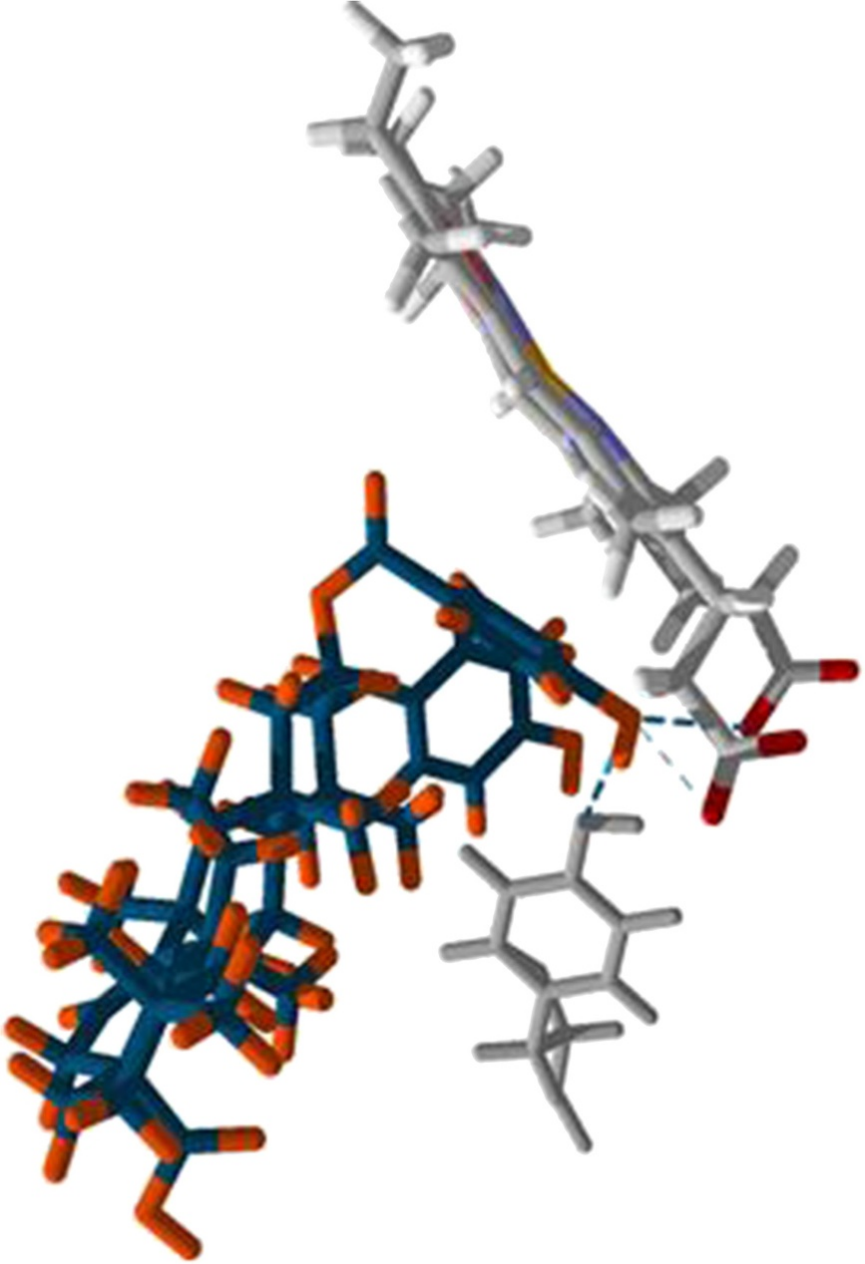
**The lowest energy pose of 2,3-bis-(4-hydroxybenzoyl) derivative of sebiferenic acid is predicted to interact via hydrogen bonding with the heme co-factor in the active site of** ***T. cruzi*** **CYP51.** The hydrophobic amino acid residues in the active site offer a favorable binding patch for esterified sterols. The molecular surface diagram is shown in Additional file 1: Figure S4.

The triterpene-structural moiety of 3-hexadecanoyl 11,12-oxidotaraxerol is predicted to interact with Met 460, Gly 49, Pro 210, Val 213 and Val 102 residues in the hydrophobic channel of the substrate binding site (Figure 5). The 3-*O*-caffeate derivative of isomyricadiol was predicted to have hydrogen bonding interactions with residues Tyr 103 and Met 358. It has favourable steric interactions with Ala 291, Met 284, Ala 115, Met 106, Ile 105 and Met 358 and 360. In the case of the 2,3-bis-(4-hydroxybenzoyl) derivative of sebiferenic acid, it has favorable steric interactions with Met 460, Tyr 116, Ala 115 and 291, Leu 356 and Val 213 in addition to hydrogen bonding interactions with Tyr 116 and the two propionic acid moiety of the heme co-factor as shown in Figure 6 while the lowest energy pose of the 3-*O*-*p*-coumarate derivative of aleuritolic acid have 460, Tyr 103 and 457, as well as an hydrogen bonding interaction with Ile 209.

### *epi*-Oleanolic acid structural congeners and *L. infantum*CYP51

**Figure 7:**
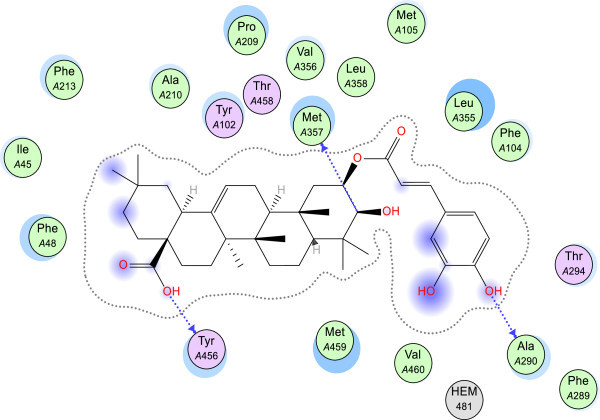
**Geometry-optimized pose of 2-** ***O*** **-caffeoyl maslinic acid.** The blue dash arrow represents hydrogen bonding interactions while the blue sphere on the residues denotes level of solvent exposure. Hydrophobic residues are olive green while polar residues are depicted as pink. The purple circles on the atoms/functional groups represent solvent exposure. The molecular surface diagram is shown in Additional file 1: Figure S5.

**Figure 8:**
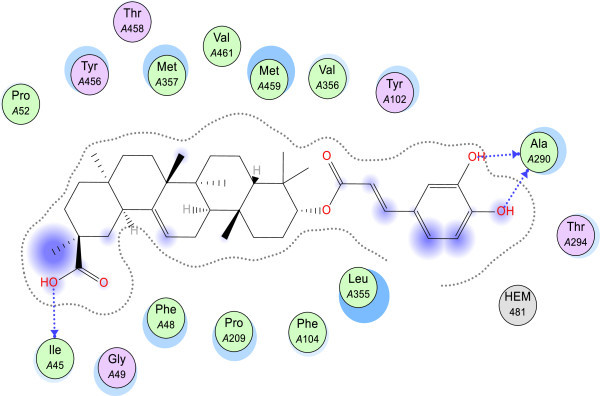
**Geometry-optimized pose of 3-O-caffeoyl-20-epikatonic acid.** The blue dash arrow represents hydrogen bonding interactions while the blue sphere on the residues denotes level of solvent exposure. Hydrophobic residues are olive green while polar residues are depicted as pink. The purple circles on the atoms/functional groups represent solvent exposure. The molecular surface diagram is shown in Additional file 1: Figure S6.

**Figure 9:**
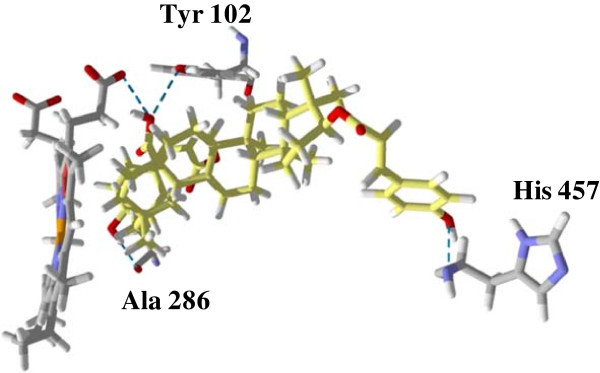
**The lowest energy pose of asprellic acid B.** Hydrogen bonding interactions with Tyr 102, Ala 286, heme-cofactor and His 457 are predicted as blue dash lines. The molecular surface diagram is shown in Additional file 1: Figure S7.

Several derivatives of oleanolic acid including *epi*-oleanolic acid have been reported to have weak to moderate antiprotozoal activities (Schmidt et al. 2012), however, the molecular targets or the mechanism(s) of action of these compounds are yet to be described. The results from this work showed that *epi*-oleanolic acid and a number of its congener have high docking affinities for *L. infantum* CYP51 relative to other antiparasitic sterols. In a similar fashion to taraxerol derivatives described above, the structural congeners of *epi*-oleanolic acids that have high docking affinity for *L. infantum* CYP51 possess either long hydrophobic tails or hydroxycinnamoyl groups (Figure 4 and Additional file 1: Table S5). These hydroxycinnamoyl groups were predicted to hydrogen bond with Ala 290, and they also have extensive steric interactions with the heme-cofactor (Figures 7 and 8). The 2-*O*-caffeate derivative of maslinic acid was predicted to have significant interactions (in terms of energy values) with Leu 355, Met 357 and Met 359, Phe 48, Tyr 102, and Val 356. Similarly, the 3-*O*-caffeate derivative of 20-epikatonic acid has significant interactions with Leu 355, Met 357 and 459, Phe 48, and Val 356. In the case of the asprellic acid B, the free acid group at position 28 was predicted to hydrogen bond with the heme co-factor while the caffeoyl moieties hydrogen bond with Ala 286 and His 457 (Figure 9). Cinnamate esters or hydroxycinnamates have not been reported to have inhibitory activity against CYP51. However, similar motifs have been reported to inhibit *Cochliobolus lunatus* benzoate hydroxylate (CYP53) (Korošec et al. 2014).

### α/β-amyrin structural congeners and *T. cruzi*CYP51

**Figure 10:**
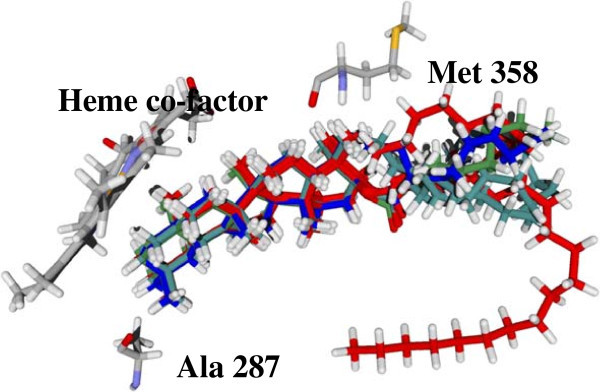
**The top five structural congeners of β-Amyrin.** An overlay of all five compounds shows that the sterol motif overlaps, and interacts with Ala 287 and Met 358. The molecular surface diagram is shown in Additional file 1: Figure S8.

The ubiquitous pentacyclic triterpenes α- and β-amyrin, as well as, their derivatives have been reported to exhibit antiprotozoal activities, amongst many other biological activities (Hoet et al. 2007; Mwangi et al. 2010; Schinor et al. 2007). However, they have not been reported to inhibit sterol 14α demethylase. In fact, the multifunctional cytochrome P450 AsCYP51H10 in Oats (*Avena* spp) has been shown to catalyze the epoxidation and hydroxylation of β-amyrin. The structural congeners of β-amyrin that have high affinity for *T. cruzi* CYP51 include β-amyrin eicosanoate, β-amyrin stearate, β-amyrin behenate, β-amyrin lineolate and β-amyrin palmitoleate. The sterol motif of those compounds was predicted to interact with similar residues in the TcCYP51 active site. These residues include Met 358 and 460, Leu 356, Ala 287, Tyr 103 and Lys 368. There is an exact spatial overlap of the sterol motif in the docking poses of the five lowest-energy fatty esters of β-amyrin (as shown in Figure 10) and steric interactions is the most predominate interactions predicted between *T. cruzi* CYP51 and these fatty esters. The hydrophobic tail of β-amyrin eicosanoate was predicted to adopt a kink conformation and was predicted to have steric interactions with His 468.

## Conclusion

The important findings from this work are 1) majority of antiparasitic sterol-like compounds preferentially dock to the human CYP51, perhaps, this is due to the relatively large size of its active site, 2) taraxerol, α/β-amyrin and a number of their fatty acyl esters and cinnamate derivatives have preferential affinity for *T. cruzi*’s CYP51, 3) *epi*-oleanolic and its hydroxycinnamate derivatives have high docking affinities for *L. infantum’s* CYP51. Those predictions can be validated experimentally and used as guide for future natural products or synthetic chemistry target-based (CYP51) drug discovery. It is important to note that, generally, docking scores from multiple crystallographic structures of the same protein tend to be similar. But, we also noticed that a significant number of compounds have substantially different scores in similar subunits of oligomeric structures. This is perhaps due to protein flexibility captured in the crystallographic structures. These subtle differences in protein structures perhaps result in prediction of interactions between the small molecules and different amino acid residues in the binding sites of similar subunits of oligomeric proteins. Also, it has not escaped us that the compounds identified from the simulations can also serve as substrates for any of the CYP51s and that most of the compounds we identified as potential inhibitors of CYP51 in this work may not have ideal drug-like ADMET (absorption, distribution, metabolism, excretion and toxicity) properties. However, structural optimization can help to introduce functional groups that can serve as inhibitory warheads and that can also improve ADMET properties.

## Electronic supplementary material

Additional file 1: **Docking (MVD re-rank) scores of antiparasitic sterols and CYP51.** (DOCX 4 MB)

Additional file 2: **PDB files of CYP51-ligand complexes.** (ZIP 900 KB)

## References

[CR1] Andriani G, Amata E, Beatty J, Clements Z, Coffey BJ, Courtemanche G, Devine W, Erath J, Juda CE, Wawrzak Z, Wood JT, Lepesheva GI, Rodriguez A, Pollastri MP (2013). Antitrypanosomal lead discovery: identification of a ligand-efficient inhibitor of Trypanosoma cruzi CYP51 and parasite growth. J Med Chem.

[CR2] Aoyama Y (2005). Recent progress in the CYP51 research focusing on its unique evolutionary and functional characteristics as a diversozyme P450. Front Biosci.

[CR3] Barreiros ML, David JM, Pereira PAP, Guedes MLS, David JP (2002). Fatty acid esters of triterpenes from *Erythroxylum passerinum*. J Braz Chem Soc.

[CR4] Buckner FS, Bahia MT, Suryadevara PK, White KL, Shackleford DM, Chennamaneni NK, Hulverson MA, Laydbak JU, Chatelain E, Scandale I, Verlinde CL, Charman SA, Lepesheva GI, Gelb MH (2012). Pharmacological characterization, structural studies, and in vivo activities of anti-Chagas disease lead compounds derived from tipifarnib. Antimicrob Agents Chemother.

[CR5] Chen CK, Doyle PS, Yermalitskaya LV, Mackey ZB, Ang KK, McKerrow JH, Podust LM (2009). *Trypanosoma cruzi* CYP51 inhibitor derived from a *Mycobacterium tuberculosis* screen hit. PLoS Negl Trop Dis.

[CR6] Cole ST, Brosch R, Parkhill J, Garnier T, Churcher C, Harris D, Gordon SV, Eiglmeier K, Gas S, Barry CE, Tekaia F, Badcock K, Basham D, Brown D, Chillingworth T, Connor R, Davies R, Devlin K, Feltwell T, Gentles S, Hamlin N, Holroyd S, Hornsby T, Jagels K, Krogh A, McLean J, Moule S, Murphy L, Oliver K, Osborne J (1998). Deciphering the biology of *Mycobacterium tuberculosis* from the complete genome sequence. Nature.

[CR7] Doyle PS, Chen CK, Johnston JB, Hopkins SD, Leung SS, Jacobson MP, Engel JC, McKerrow JH, Podust LM (2010). A nonazole CYP51 inhibitor cures Chagas’ disease in a mouse model of acute infection. Antimicrob Agents Chemother.

[CR8] Gachet MS, Kunert O, Kaiser M, Brun R, Zehl M, Keller W, Muñoz RA, Bauer R, Schuehly W (2011). Antiparasitic compounds from *Cupania cinerea* with activities against *Plasmodium falciparum* and *Trypanosoma brucei rhodesiense*. J Nat Prod.

[CR9] Geisler K, Hughes RK, Sainsbury F, Lomonossoff GP, Rejzek M, Fairhurst S, Olsen CE, Motawia MS, Melton RE, Hemmings AM, Bak S, Osbourn A (2013). Biochemical analysis of a multifunctional cytochrome P450 (CYP51) enzyme required for synthesis of antimicrobial triterpenes in plants. Proc Natl Acad Sci USA.

[CR10] GraphPad Prism version 6.00 for Windows (2013). GraphPad Software.

[CR11] Gunatilleke SS, Calvet CM, Johnston JB, Chen CK, Erenburg G, Gut J, Engel JC, Ang KK, Mulvaney J, Chen S, Arkin MR, McKerrow JH, Podust LM (2012). Diverse inhibitor chemotypes targeting *Trypanosoma cruzi* CYP51. PLoS Negl Trop Dis.

[CR12] Hargrove TY, Wawrzak Z, Liu J, Nes WD, Waterman MR, Lepesheva GI (2011). Substrate preferences and catalytic parameters determined by structural characteristics of sterol 14alpha-demethylase (CYP51) from *Leishmania infantum*. J Biol Chem.

[CR13] Hargrove TY, Wawrzak Z, Alexander PW, Chaplin JH, Keenan M, Charman SA, Perez CJ, Waterman MR, Chatelain E, Lepesheva GI (2013). Complexes of *Trypanosoma cruzi* sterol 14α-demethylase (CYP51) with two pyridine-based drug candidates for Chagas disease: structural basis for pathogen selectivity. J Biol Chem.

[CR14] Hargrove TY, Wawrzak Z, Waterman MR: **CYP51 structure-based VNI scaffold development.** doi:10.2210/pdb4g3j/pdb

[CR15] Hoet S, Pieters L, Muccioli GG, Habib-Jiwan JL, Opperdoes FR, Quetin-Leclercq J (2007). Antitrypanosomal activity of triterpenoids and sterols from the leaves of *Strychnos spinosa* and related compounds. J Nat Prod.

[CR16] Ibrahim SA, Ali MS (2007). Constituents of *Nepeta crassifolia* (Lamiaceae). Turk J Chem.

[CR17] Korošec B, Sova M, Turk S, Kraševec N, Novak M, Lah L, Stojan J, Podobnik B, Berne S, Zupanec N, Bunc M, Gobec S, Komel R (2014). Antifungal activity of cinnamic acid derivatives involves inhibition of benzoate 4-hydroxylase (CYP53). J Appl Microbiol.

[CR18] Lepesheva GI, Hargrove TY, Anderson S, Kleshchenko Y, Furtak V, Wawrzak Z, Villalta F, Waterman MR (2010). Structural insights into inhibition of sterol 14alpha-demethylase in the human pathogen *Trypanosoma cruzi*. J Biol Chem.

[CR19] Lorente SO, Rodrigues JC, Jiménez Jiménez C, Joyce-Menekse M, Rodrigues C, Croft SL, Yardley V, de Luca-Fradley K, Ruiz-Pérez LM, Urbina J, de Souza W, González Pacanowska D, Gilbert IH (2004). Novel azasterols as potential agents for treatment of leishmaniasis and trypanosomiasis. Antimicrob Agents Chemother.

[CR20] McLean KJ, Marshall KR, Richmond A, Hunter IS, Fowler K, Kieser T, Gurcha SS, Besra GS, Munro AW (2002). Azole antifungals are potent inhibitors of cytochrome P450 mono-oxygenases and bacterial growth in mycobacteria and streptomycetes. Microbiology.

[CR21] Mwangi ESK, Keriko JM, Machocho AK, Wanyonyi AW, Malebo HM, Chhabra SC, Tarus PK (2010). Antiprotozoal activity and cytotoxicity of metabolites from leaves of *Teclea trichocarpa*. J Med Plants Res.

[CR22] Ogungbe IV, Setzer WN (2013). *In-silico* Leishmania target selectivity of antiparasitic terpenoids. Molecules.

[CR23] Peng XM, Cai GX, Zhou CH (2013). Recent developments in azole compounds as antibacterial and antifungal agents. Curr Top Med Chem.

[CR24] Podust LM, von Kries JP, Eddine AN, Kim Y, Yermalitskaya LV, Kuehne R, Ouellet H, Warrier T, Alteköster M, Lee JS, Rademann J, Oschkinat H, Kaufmann SH, Waterman MR (2007). Small-molecule scaffolds for CYP51 inhibitors identified by high-throughput screening and defined by X-ray crystallography. Antimicrob Agents Chemother.

[CR25] Qi X, Bakht S, Qin B, Leggett M, Hemmings A, Mellon F, Eagles J, Werck-Reichhart D, Schaller H, Lesot A, Melton R, Osbourn A (2006). A different function for a member of an ancient and highly conserved cytochrome P450 family: from essential sterols to plant defense. Proc Natl Acad Sci USA.

[CR26] Rozhon W, Husar S, Kalaivanan F, Khan M, Idlhammer M, Shumilina D, Lange T, Hoffmann T, Schwab W, Fujioka S, Poppenberger B (2013). Genetic variation in plant CYP51s confers resistance against voriconazole, a novel inhibitor of brassinosteroid-dependent sterol biosynthesis. PLoS One.

[CR27] Schinor EC, Salvador MJ, Pral EMF, Alfieri SC, Albuquerque S, Dias DA (2007). Biological activities and chemical composition of crude extracts from *Chresta exsucca*. Braz J Pharmaceut Sc.

[CR28] Schmidt TJ, Khalid SA, Romanha AJ, Alves TM, Biavatti MW, Brun R, Da Costa FB, de Castro SL, Ferreira VF, de Lacerda MV, Lago JH, Leon LL, das Neves Amorim RC, Niehues M, Ogungbe IV, Pohlit AM, Scotti MT, Setzer WN, NC Soeiro M, Steindel M, Tempone AG (2012). The potential of secondary metabolites from plants as drugs or leads against protozoan neglected diseases - part I. Curr Med Chem.

[CR29] Setzer WN, Ogungbe IV (2012). *In-silico* investigation of antitrypanosomal phytochemicals from Nigerian medicinal plants. PLoS Negl Trop Dis.

[CR30] Spartan’10 for Windows, v 1.1 (2011). Wavefunction, Inc.

[CR31] Strushkevich N, Usanov SA: **Crystal structure of human lanosterol 14alpha-demethylase (CYP51) in complex with econazole.** doi:10.2210/pdb3jus/pdb

[CR32] Strushkevich N, Usanov SA, Park HW (2010). Structural basis of human CYP51 inhibition by antifungal azoles. J Mol Biol.

[CR33] The Dictionary of Natural Products (2013). DVD-ROM.

[CR34] Thomsen R, Christensen MH (2006). MolDock: a new technique for high-accuracy molecular docking. J Med Chem.

[CR35] Urbina JA, Payares G, Molina J, Sanoja C, Liendo A, Lazardi K, Piras MM, Piras R, Perez N, Wincker P, Ryley JF (1996). Cure of short- and long-term experimental Chagas’ disease using D0870. Science.

[CR36] *Molegro Virtual Docker v 5.0*. Molegro ApS, Aarhus, Denmark; 2011.

