# Supplementary material for: Interactions of antiparasitic sterols with sterol 14α-demethylase (CYP51) of human pathogens
Source: Springerplus. 2014 Nov 20;3:679. doi: 10.1186/2193-1801-3-679 (PMC4410773; doi:10.1186/2193-1801-3-679)
Supplement: Supplementary file 1 — Additional file 1: Docking (MVD re-rank) scores of antiparasitic sterols and CYP51. (DOCX 4 MB) [file 40064_2014_1378_MOESM1_ESM.docx]

**Supplementary Material**

Interactions of antiparasitic sterols with sterol 14α-demethylase (CYP51) of human pathogens.

Jasmine Warfield*^a^,* William N. Setzer*^b^* and Ifedayo Victor Ogungbe*^a*^*

*^a^* Department of Chemistry & Biochemistry, Jackson State University, Jackson, MS 39217, USA

*^b^* Department of Chemistry, University of Alabama in Huntsville, Huntsville, AL 35899, USA

***Correspondence:** Ifedayo.v.ogungbe@jsums.edu.

**Supplementary Fig.1** Docking energies (re-rank score) of antiparasitic sterols


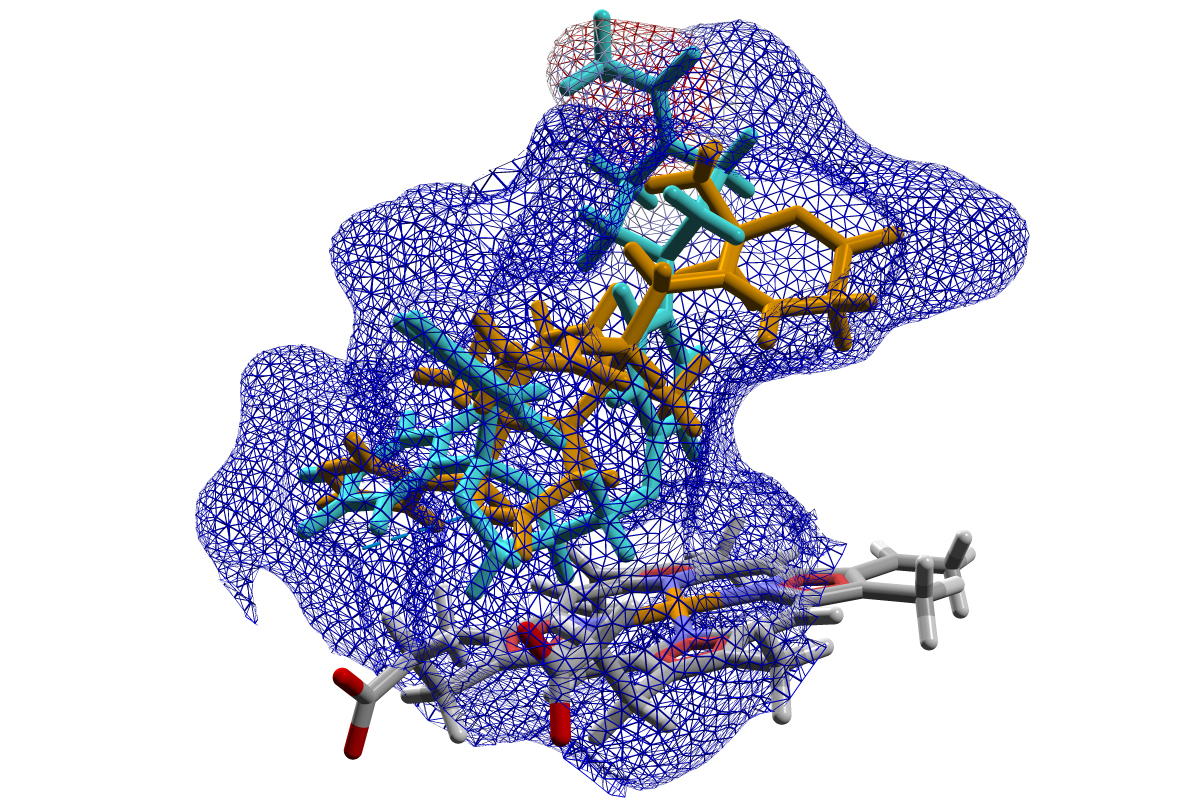


Supplementary Fig. 2 The lowest energy docking poses of carapolide A (Orange) and ketoconazole (Blue) in the substrate binding site of human CYP51. The blue line tracings depicts the molecular surface of CYP51 active site


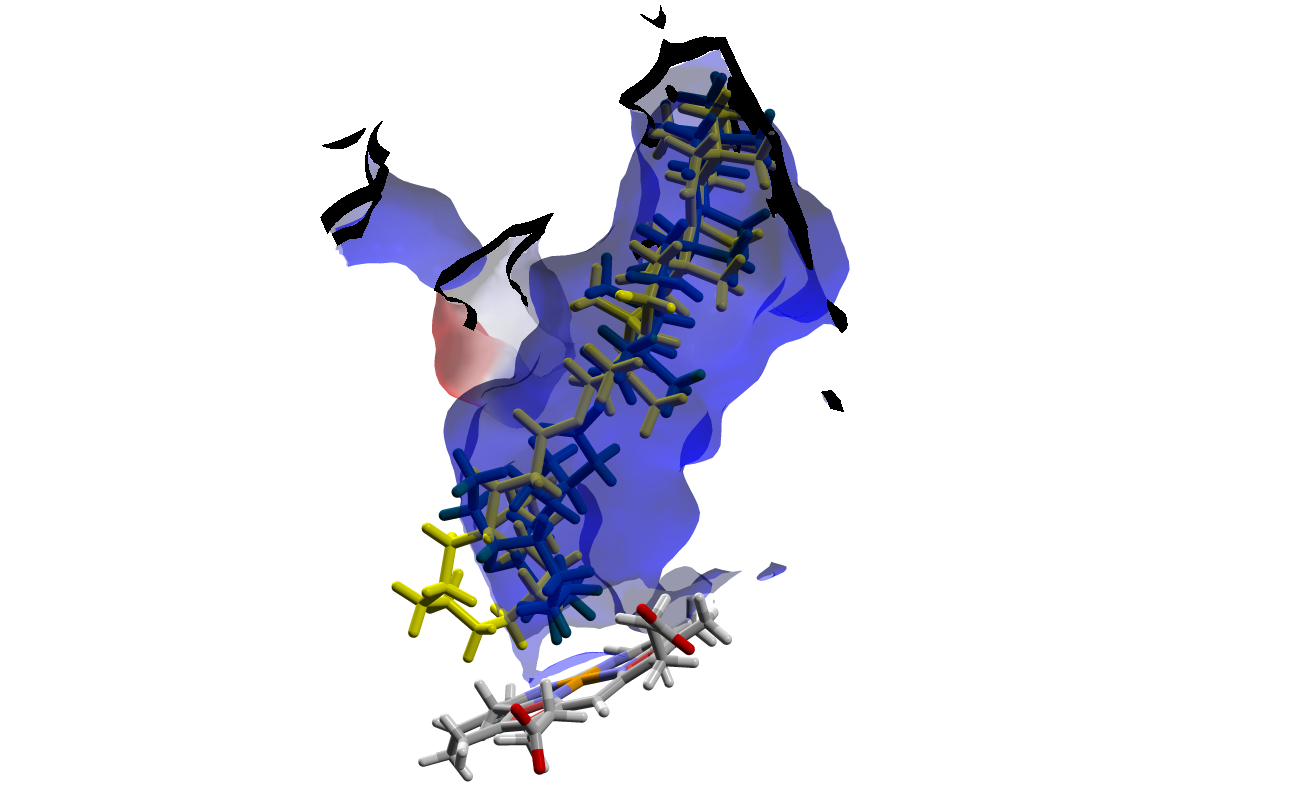


**Supplementary Fig. 3** The lowest energy pose of 3-hexadecanoyl 11,12-oxidotaraxerol (yellow) and crassifoate (blue) in the active site of *T. cruzi* CYP51. The blue shading depicts the molecular surface of *T. cruzi* CYP51 active site


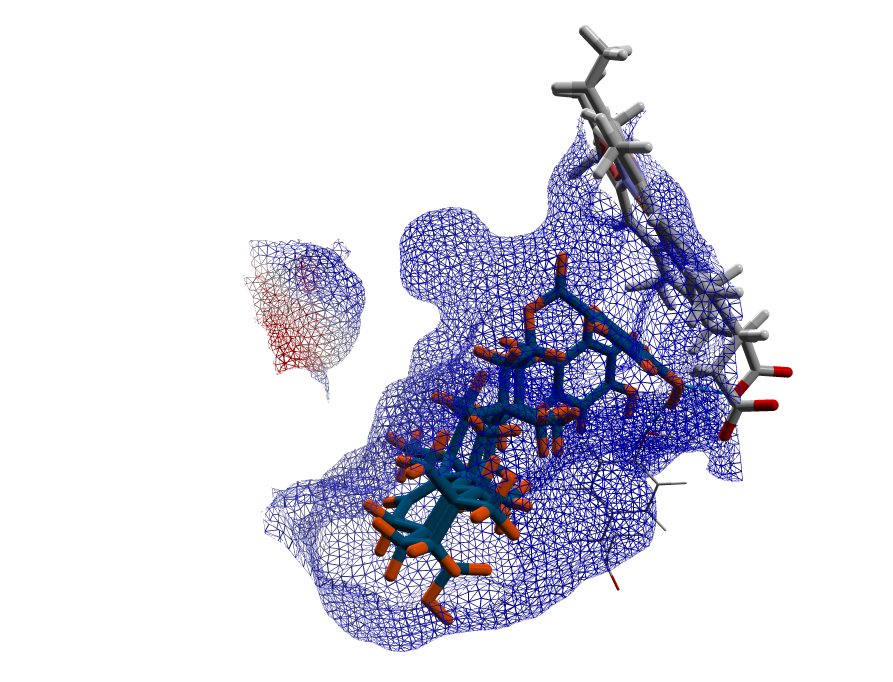


**Supplementary Fig. 4** The lowest energy pose of 2,3-bis-(4-hydroxybenzoyl) derivative of sebiferenic acid is predicted to interact via hydrogen bonding with the heme co-factor in the active site of *T. cruzi* CYP51. The blue line tracings depicts the molecular surface of *T. cruzi* CYP51 active site

**
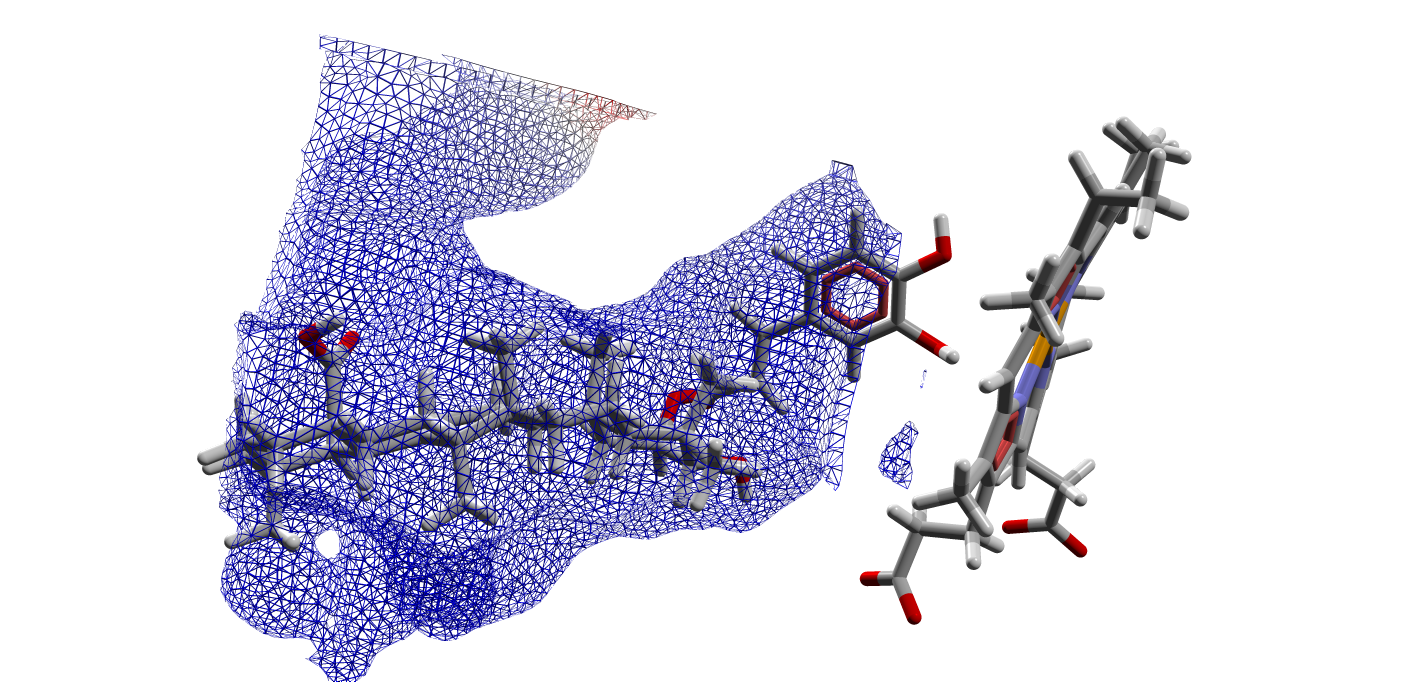
**

**Supplementary Fig. 5** The lowest energy pose of 2-*O*-caffeoyl maslinic acid in the active site of *L. infantum* CYP51. The blue line tracings depicts the molecular surface of *L. infantum* CYP51 active site

**
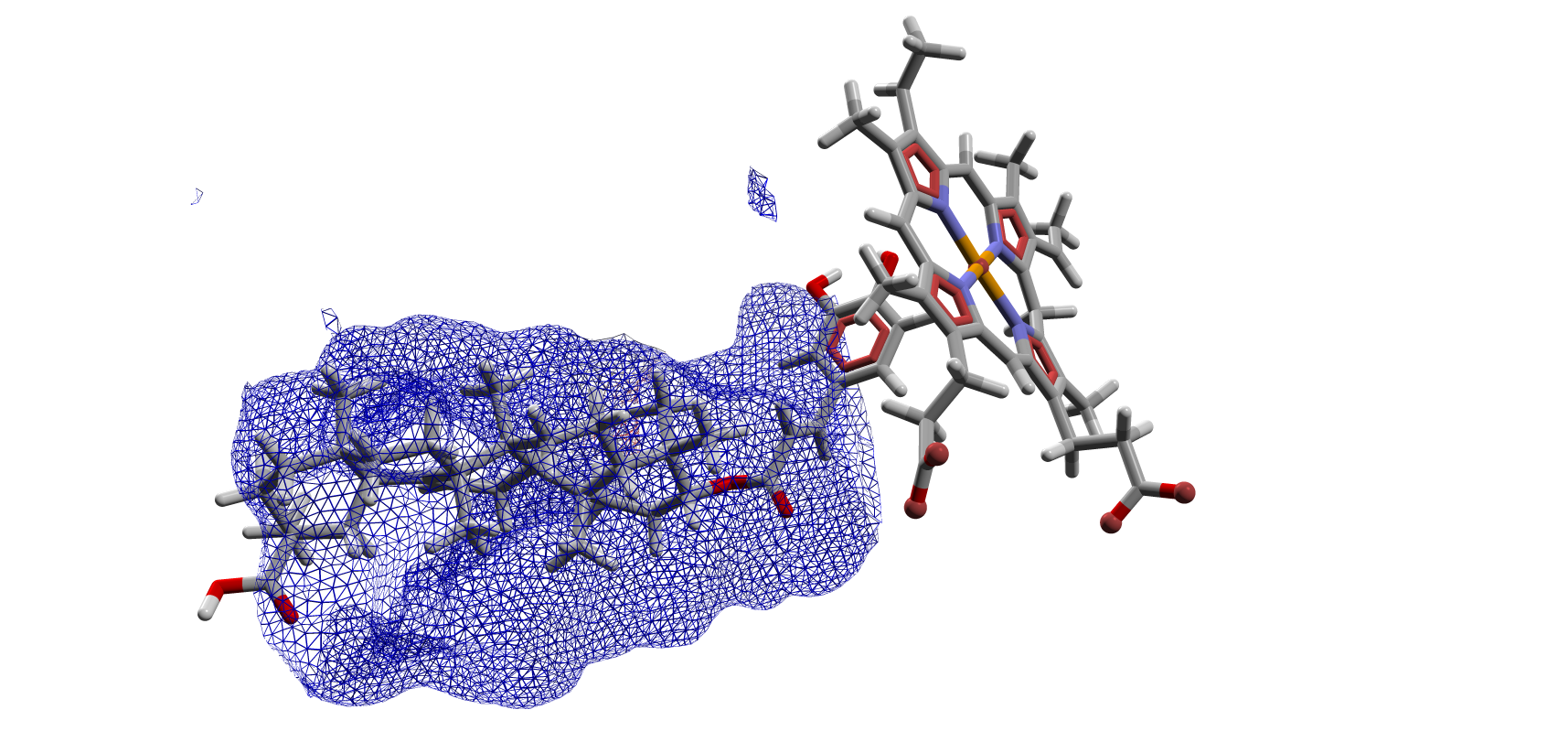
**

**Supplementary Fig. 6** The lowest energy pose of 3-*O*-caffeoyl-20-epikatonic acid in the active site of *L. infantum* CYP51. The blue line tracings depicts the molecular surface of *L. infantum* CYP51 active site

**
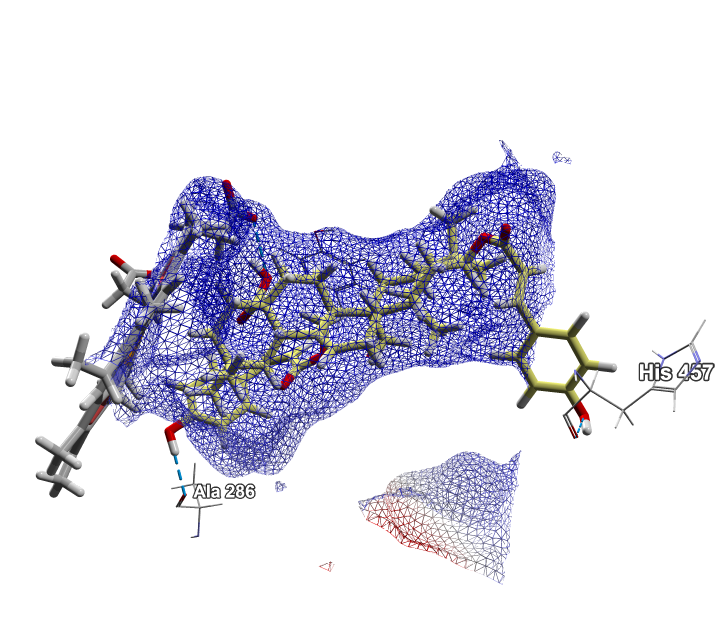
**

**Supplementary Fig. 7** The lowest energy pose of asprellic acid B in the active site of *L. infantum* CYP51. The blue line tracings depicts the molecular surface of *L. infantum* CYP51 active site

**
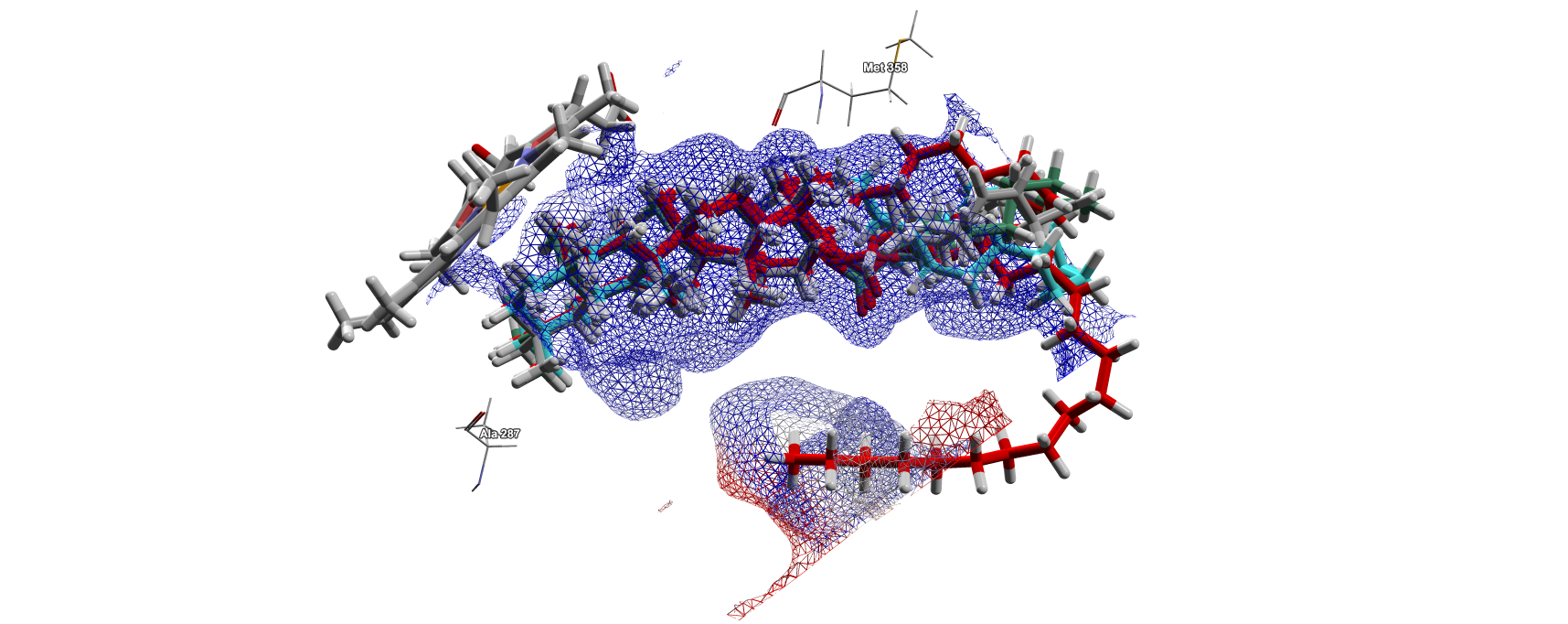
**

**Supplementary Fig. 8** The lowest energy pose of top five structural congeners of *β*-Amyrin in the active site of *T. cruzi* CYP51. The blue line tracings depicts the molecular surface of *T. cruzi* CYP51 active site

**Supplementary Table 1:** Docking energies of antiparasitic sterol and triterpene derivatives to parasitic and human CYP51 (KJ/mol).

| **Antiparasitic Compounds** | ***Trypanosoma cruzi*** | ***Trypanosoma brucei*** | ***Mycobacterium tuberculosis*** | ***Leishmania infantum*** | ***Homo sapiens*** |
| --- | --- | --- | --- | --- | --- |
| (24Z)-3-Oxotirucalla-7, 24-dien-26-oic acid | -75.69 | -107.23 | -61.17 | -95.37 | -121.59 |
| 1,3,7-Trideacetylkhivorin | -85.31 | -93.09 | -75.89 | -41.19 | -103.12 |
| 11α-acetoxy-2α-hydroxy-6-deoxyswietenine acetate | -55.10 | -105.11 | -98.45 | -101.20 | -122.04 |
| 14-Hydroxyixocarpanolide | -56.74 | -99.43 | -55.87 | -103.76 | -111.85 |
| 15-β-Heptylchaparrinone | -111.28 | -114.37 | -68.62 | -96.21 | -112.20 |
| 1-Deacetylkhivorin | -109.62 | -108.50 | -103.20 | -101.04 | -116.24 |
| 1-*O*-Acetylkhayanolide A | -106.95 | -102.14 | -110.50 | -91.50 | -114.72 |
| 1-*O*-Acetylkhayanolide B | -102.69 | -106.06 | -101.18 | -107.00 | -96.52 |
| 1-*O*-Deacetyl-2α-hydroxykhayanolide E | -83.51 | -101.51 | -92.33 | -110.86 | -106.05 |
| 1-*O*-Deacetyl-6-deoxykhayanolide E | -102.77 | -93.91 | -81.73 | -94.69 | -103.76 |
| 20-epi-Isoiguesterinol | -101.21 | -91.86 | -79.23 | -34.73 | -88.54 |
| 24, 25-Epoxywithanolide D | -80.37 | -128.42 | -107.05 | -128.05 | -114.83 |
| 24-Hydroperoxy-24-vinylcholesterol | -117.56 | -102.42 | -60.86 | -120.98 | -107.38 |
| 3,7-Dideacetylkhivorin | -83.66 | -92.80 | -77.78 | -80.65 | -83.30 |
| 3-Acetylkhayalactone | -85.52 | -102.52 | -90.28 | -104.17 | -142.12 |
| 3-Deacetylkhivorin | -105.55 | -88.25 | -75.18 | -104.52 | -85.90 |
| 3-O-Acetylanthothecanolide | -75.28 | -76.25 | -106.21 | -102.19 | -121.26 |
| 3-O-Acetylswietenine | -104.59 | -95.24 | -96.16 | -83.72 | -123.39 |
| 3-O-Acetylswietenolide | -99.15 | -100.94 | -104.88 | -103.19 | -97.97 |
| 3-*O*-Methyl-6-oxopristimerol | -85.85 | -86.76 | -52.88 | -12.82 | -107.35 |
| 3β-Isobutyryloxy-1-oxomeliac-8(30)-enate | -85.01 | -93.62 | -80.98 | -102.61 | -123.99 |
| 6-Acetylswietenolide | -74.94 | -93.02 | -103.67 | -102.68 | -117.90 |
| 6-Deoxyswietenolide | -84.61 | -93.77 | -87.68 | -92.13 | -109.07 |
| 6-Hydroxykhayalactone | -109.11 | -111.47 | -112.26 | -84.50 | -122.19 |
| 6-Oxopristimerol | -83.81 | -84.17 | -86.93 | -44.05 | -104.59 |
| 7-Deacetoxy-7-oxogedunin | -86.74 | -82.42 | -49.23 | -104.35 | -117.38 |
| 7-Deacetylgedunin | -93.46 | -81.15 | -77.19 | -80.69 | -119.47 |

**Supplementary Table 1 Cont’d:** Docking energies of antiparasitic sterol and triterpene derivatives to parasitic and human CYP51.

| **Antiparasitic Compounds** | ***Trypanosoma cruzi*** | ***Trypanosoma brucei*** | ***Mycobacterium tuberculosis*** | ***Leishmania infantum*** | ***Homo sapiens*** |
| --- | --- | --- | --- | --- | --- |
| 7-Deacetylkhivorin | -101.82 | -70.02 | -114.07 | -88.21 | -44.59 |
| Anthothecanolide | -98.62 | -104.88 | -52.14 | -107.93 | -118.66 |
| Betulin | -97.31 | -98.56 | -85.27 | -101.58 | -85.67 |
| Betulinaldehyde | -93.01 | -101.50 | -68.91 | -100.10 | -84.43 |
| Betulinic acid | -56.35 | -88.29 | -87.52 | -91.19 | -94.93 |
| Carapa spirolactone | -96.90 | -93.49 | -109.86 | -98.42 | -108.13 |
| Carapin | -92.33 | -113.02 | -86.10 | -83.39 | -111.75 |
| Carapolide A | -92.49 | -116.99 | -101.68 | -103.04 | -130.87 |
| Carapolide B | -97.58 | -119.29 | -94.32 | -85.63 | -115.98 |
| Carapolide C | -95.52 | -108.99 | -48.30 | -104.45 | -108.91 |
| Cholesterol | -100.76 | -99.35 | -83.22 | -110.47 | -113.18 |
| Clerosterol | -108.83 | -101.72 | -78.15 | -116.34 | -115.99 |
| Colosolic acid | -100.45 | -91.16 | -74.77 | -111.96 | -90.29 |
| Deacetylkhayanolide E | -90.13 | -95.04 | -95.06 | -103.24 | -107.76 |
| epi-Oleanolic acid | -36.96 | -95.46 | -88.21 | -105.70 | -85.84 |
| Erythrodiol | -108.85 | -83.43 | -82.80 | -100.52 | -86.87 |
| Evodulone | -113.14 | -100.20 | -74.29 | -104.13 | -106.09 |
| Fissinolide | -90.67 | -91.17 | -106.72 | -103.31 | -106.03 |
| Friedelin | -99.98 | -82.90 | -39.59 | -72.16 | -89.96 |
| Gedunin | -92.63 | -89.65 | -84.92 | -107.45 | -128.70 |
| Grandifolide A | -33.81 | -106.85 | -104.94 | -104.49 | -128.36 |
| Grandifolin | -43.69 | -93.97 | -85.81 | -107.60 | -109.06 |
| Grandifoliolenone | -102.88 | -88.62 | -90.24 | -12.45 | -109.48 |
| Grandifotane | -97.59 | -105.53 | -94.69 | -108.83 | -118.13 |
| Isoiguesterin | -94.01 | -85.49 | -70.92 | -83.68 | -78.90 |
| Khayalactone | -70.19 | -104.60 | -109.45 | -98.12 | -124.31 |
| Khayanolide A | -111.27 | -114.72 | -91.64 | -108.76 | -122.82 |

**Supplementary Table 1 Cont’d:** Docking energies of antiparasitic sterol and triterpene derivatives to parasitic and human CYP51.

| **Antiparasitic Compounds** | ***Trypanosoma cruzi*** | ***Trypanosoma brucei*** | ***Mycobacterium tuberculosis*** | ***Leishmania infantum*** | ***Homo sapiens*** |
| --- | --- | --- | --- | --- | --- |
| Khayanolide B | -92.28 | -95.56 | -87.28 | -103.11 | -109.63 |
| Khivorin | -111.73 | -97.53 | -101.92 | -94.53 | -96.59 |
| Lanosterol | -108.23 | -91.74 | -90.00 | -113.50 | -102.63 |
| Lupeol | -89.26 | -94.85 | 1.58 | -98.01 | -93.34 |
| Methyl acetoxyangolensate | -93.38 | -98.70 | -89.79 | -89.97 | -100.75 |
| Methyl angolensate | -85.91 | -91.26 | 1.19 | -88.10 | -111.73 |
| Methyl hydroxyangolensate | -75.15 | -98.19 | -24.72 | -68.58 | -112.95 |
| Methyl ivorensate | -60.78 | -90.00 | -52.35 | -96.15 | -116.99 |
| Methyl lawnermate | -102.51 | -94.97 | -89.97 | -90.35 | -92.08 |
| Methyl seco-34-betulonic acid | -101.05 | -106.92 | -75.95 | -92.79 | -100.36 |
| Mexicanolide | -67.81 | -99.40 | -91.57 | -91.06 | -103.09 |
| Oleanolic acid | -96.25 | -91.21 | -61.28 | -105.44 | -84.28 |
| Physagulin A | -104.41 | -119.21 | -105.19 | -88.44 | -105.36 |
| Physagulin B | -118.20 | -101.21 | -106.13 | -99.50 | -111.70 |
| Physagulin C | -118.85 | -116.40 | -70.40 | -122.47 | -111.81 |
| Physagulin F | -112.71 | -111.55 | -35.92 | -100.29 | -119.66 |
| Physagulin H | -100.40 | -107.69 | -97.04 | -115.25 | -116.33 |
| Physagulin I | -110.77 | -97.44 | -53.73 | -96.95 | -116.94 |
| Physagulin J | -118.32 | -120.93 | -107.25 | -99.34 | -125.92 |
| Physagulin K | -94.46 | -83.29 | -16.34 | -105.20 | -120.69 |
| Physagulin L | -101.61 | -103.34 | -97.03 | -108.95 | -121.76 |
| Physagulin L' | -92.18 | -103.08 | -105.02 | -96.22 | -114.81 |
| Physagulin M | -111.57 | -117.73 | -84.62 | -103.70 | -99.35 |
| Physagulin M' | -103.18 | -108.19 | -101.80 | -85.65 | -120.12 |
| Physagulin N | -104.30 | -120.61 | -86.24 | -113.67 | -120.87 |
| Physagulin N' | -105.37 | -97.97 | -85.95 | -94.62 | -107.98 |
| Physagulin O | -112.98 | -110.87 | -88.78 | -95.75 | -114.72 |

**Supplementary Table 1 Cont’d:** Docking energies of antiparasitic sterol and triterpene derivatives to parasitic and human CYP51.

| **Antiparasitic Compounds** | ***Trypanosoma cruzi*** | ***Trypanosoma brucei*** | ***Mycobacterium tuberculosis*** | ***Leishmania infantum*** | ***Homo sapiens*** |
| --- | --- | --- | --- | --- | --- |
| Physalin A | -93.04 | -101.80 | -80.77 | -104.92 | -95.65 |
| Physalin B | -70.02 | -103.07 | -69.12 | -110.58 | -101.68 |
| Physalin D | -18.68 | -119.75 | -68.72 | -113.53 | -107.19 |
| Physalin E | -88.94 | -101.17 | -44.33 | -115.31 | -72.99 |
| Physalin F | -7.35 | -111.57 | -72.27 | -111.89 | -104.25 |
| Physalin G | -80.21 | -119.60 | -62.93 | -94.37 | -101.92 |
| Physalin H | -84.49 | -112.64 | -70.80 | -112.40 | -108.72 |
| Physalin I | -89.80 | -94.82 | -80.33 | -83.80 | -112.08 |
| Physalin J | -10.20 | -104.88 | -18.73 | -111.03 | -101.44 |
| Physalin K | -6.38 | -112.10 | -84.64 | -93.95 | -88.71 |
| Physalin U | -88.96 | -92.47 | -84.76 | -96.93 | -110.30 |
| Physalin V | -104.31 | -97.95 | -86.27 | -114.56 | -77.36 |
| Physalin W | -91.58 | -85.92 | -87.04 | -91.39 | -91.44 |
| Physangulide | -93.43 | -92.51 | -94.24 | -116.51 | -108.81 |
| Physanolide A | -95.45 | -105.89 | -64.18 | -75.73 | -103.29 |
| Pristimerin | -98.51 | -101.99 | -79.13 | -102.54 | -100.59 |
| Proceranolide | -76.32 | -94.02 | -93.11 | -95.72 | -106.77 |
| Proceranolide butanoate | -93.15 | -104.40 | -105.35 | -92.61 | -108.37 |
| Proceranone | -97.29 | -102.63 | -102.71 | -99.62 | -110.35 |
| Procerin | 127.95 | -121.45 | -85.38 | -48.59 | -49.28 |
| Rotundic acid | -55.54 | -90.01 | -98.36 | -107.13 | -107.78 |
| Saringosterol | -112.30 | -102.54 | -94.67 | -121.96 | -106.64 |
| Seneganolide | -103.06 | -93.86 | -57.67 | -101.91 | -115.77 |
| Simalikalactone D | -73.64 | -96.85 | -84.80 | -74.86 | -102.95 |
| Stigmasterol | -105.51 | -102.72 | -77.63 | -116.33 | -118.61 |
| Swiemahogin A | -119.12 | -109.84 | -95.03 | -116.89 | -127.18 |
| Swietenine | -107.83 | -112.71 | -113.21 | -107.57 | -128.02 |

**Supplementary Table 1 Cont’d:** Docking energies of antiparasitic sterol and triterpene derivatives to parasitic and human CYP51.

| **Antiparasitic Compounds** | ***Trypanosoma cruzi*** | ***Trypanosoma brucei*** | ***Mycobacterium tuberculosis*** | ***Leishmania infantum*** | ***Homo sapiens*** |
| --- | --- | --- | --- | --- | --- |
| Swietenolide | -78.55 | -95.74 | -98.06 | -93.50 | -107.93 |
| Taraxerol | -104.66 | -74.76 | -46.68 | -89.50 | -88.06 |
| Ursolic acid | -110.93 | -79.51 | -82.65 | -102.30 | -90.24 |
| Uvaol | -106.26 | -90.14 | -80.28 | -101.13 | -90.89 |
| Vamonolide | -94.79 | -90.64 | -84.87 | -88.26 | -105.00 |
| Wallichianol | -104.50 | -106.27 | -39.31 | -117.19 | -106.26 |
| Withangulatin A | -109.41 | -122.58 | -107.55 | -116.31 | -121.64 |
| Withangulatin B | -101.75 | -95.75 | -100.03 | -113.43 | -111.39 |
| Withangulatin C | -77.63 | -115.34 | -88.31 | -105.00 | -104.55 |
| Withangulatin D | -90.35 | -104.13 | -58.44 | -83.05 | -114.84 |
| Withangulatin E | -111.26 | -99.37 | -98.89 | -120.73 | -117.38 |
| Withangulatin F | -104.04 | -103.90 | -79.09 | -97.81 | -100.12 |
| Withangulatin G | -106.38 | -103.33 | -98.41 | -115.76 | -101.12 |
| Withangulatin H | -115.98 | -90.65 | -89.43 | -119.22 | -104.56 |
| Withangulatin I | -94.56 | -120.22 | -77.25 | -118.55 | -109.31 |
| α-Amyrin | -102.40 | -80.20 | -2.53 | -99.37 | -88.79 |
| β-Amyrin | -104.77 | -79.50 | -5.48 | -98.99 | -78.49 |
| β-Sitosterol | -104.02 | -96.76 | -76.43 | -117.91 | -109.15 |

**Supplementary Table 2:** Docking energies of 20-*epi*-isoiguesterinol and isoiguesterin analogues for *T. cruzi* CYP51.

| **Compounds** | **Docking energies** | **Compounds** | **Docking energies** |
| --- | --- | --- | --- |
| Celastrol 15α-Hydroxy, 21-oxo, Me ester | -74.5 | Celastrol Me ester | -32.3 |
| Celastrol 21-Oxo, Me ester | -79.3 | Celastrol | -32.6 |
| Celastrol 21β-Hydroxy, Me ester | -93.4 | Excelsine | -73.5 |
| Celastrol 22β-Hydroxy, Me ester | -90.7 | 20-e*pi*-isoiguesterinol | -101.2 |
| Celastrol 30-Hydroxy, Me ester | -91.9 | Iguesterin 29-Hydroxy, 20β,21-dihydro | -91.3 |
| Celastrol 15α-Hydroxy, Me ester | -84.9 |  |  |

**Supplementary Table 3:** Docking energies of taraxerol analogues for *T. cruzi* CYP51.

| **Compounds** | **Docking energies** | **Compounds** | **Docking energies** |
| --- | --- | --- | --- |
| 1,2,3-Trihydroxy-14-taraxeren-28-oic acid, (1β,2α,3β)-form, 2,3-Bis(4-hydroxybenzoyl) | -70.06 | 14-Taraxerene-3,29-diol, 3α-form, 29-Carboxylic acid | -94.83 |
| 1,2,3-Trihydroxy-14-taraxeren-28-oic acid, (1β,2α,3β)-form | -64.77 | 14-Taraxerene-3,29-diol, 3α-form | -107.74 |
| 1,2,3-Trihydroxy-14-taraxeren-28-oic acid | -77.41 | 14-Taraxerene-3,29-diol, 3β-form, Di-Ac | -102.93 |
| 1,3-Dihydroxy-14-taraxeren-28-oic acid(1β,3β)-form, 3-(4-Hydroxybenzoyl) | -94.51 | 14-Taraxerene-3,29-diol, 3β-form | -78.14 |
| 1,3-Dihydroxy-14-taraxeren-28-oic acid(1β,3β)-form, 3-(4-Hydroxybenzoyl)1-ketone, 3-Ac | -108.93 | 14-Taraxerene-3,29-diol | -78.41 |
| 1,3-Dihydroxy-14-taraxeren-28-oic acid(1β,3β)-form | -75.79 | 14-Taraxerene-3,30-diol, 3α-form, 3-Ac | -100.41 |
| 1,3-Dihydroxy-14-taraxeren-28-oic acid | -98.51 | 14-Taraxerene-3,30-diol, 3α-form | -89.97 |
| 11,12-Epoxy-14-taraxeren-3-ol, (3β,11α,12α)-form, Ac | -97.50 | 14-Taraxerene-3,30-diol | -90.22 |
| 11,12-Epoxy-14-taraxeren-3-ol, (3β,11α,12α)-form, Hexadecanoyl (**3-hexadecanoyl 11,12-oxidotaraxerol**) | -150.90 | 2,3,24-Trihydroxy-12,27-cyclo-14-taraxeren-28-oic acid, (2α,3α)-form, 24-Deoxy | -92.83 |
| 11,12-Epoxy-14-taraxeren-3-ol, (3β,11α,12α)-form, Octadecanoyl (**Crassifoate**) | -150.01 | 2,3,24-Trihydroxy-12,27-cyclo-14-taraxeren-28-oic acid, (2α,3α)-form | -63.73 |
| 11,12-Epoxy-14-taraxeren-3-ol, (3β,11α,12α)-form | -101.32 | 2,3,24-Trihydroxy-12,27-cyclo-14-taraxeren-28-oic acid | -77.29 |
| 11,12-Epoxy-14-taraxeren-3-ol | -101.30 | 2,3-Dihydroxy-14-taraxeren-28-oic acid (2α,3α)-form, 2-(4-Hydroxybenzoyl) | -103.30 |
| 11,12-Epoxy-3-hydroxy-14-taraxen-16-one (3β,11α,12α)-form, 3-Ac | -88.10 | 2,3-Dihydroxy-14-taraxeren-28-oic acid (2α,3α)-form | -98.24 |
| 11,12-Epoxy-3-hydroxy-14-taraxen-16-one (3β,11α,12α)-form | -71.89 | 2,3-Dihydroxy-14-taraxeren-28-oic acid (2α,3β)-form, 2-(4-Hydroxybenzoyl) | -56.93 |
| 11,12-Epoxy-3-hydroxy-14-taraxen-16-one | -71.24 | 2,3-Dihydroxy-14-taraxeren-28-oic acid (2α,3β)-form, 3-(4-Hydroxybenzoyl) | -119.50 |
| 11,12-Epoxy-3-hydroxy-14-taraxeren-28-oic acid, (3β,11α,12α)-form | -99.93 | 2,3-Dihydroxy-14-taraxeren-28-oic acid (2α,3β)-form,2,3-Bis-(4-hydroxybenzoyl) (**2,3-bis-O-4-hydroxybenzoyl sebiferenic acid**) | -134.54 |
| 11,12-Epoxy-3-hydroxy-14-taraxeren-28-oic acid | -99.82 | 2,3-Dihydroxy-14-taraxeren-28-oic acid (2α,3β)-form | -99.15 |
| 14-Taraxeren-3-ol, 3β-form, Ac | -99.60 | 2,3-Dihydroxy-14-taraxeren-28-oic acid | -98.44 |
| 14-Taraxeren-3-ol, 3β-form, Formyl | -89.51 | 3,11,23-Trihydroxy-14-taraxeren-28-oic acid, (3β,11α)-form, 3-Ac | -117.05 |

**Supplementary Table 3 Cont’d:**  Docking energies of taraxerol analogues for *T. cruzi* CYP51.

| **Compounds** | **Docking energies** | **Compounds** | **Docking energies** |
| --- | --- | --- | --- |
| 14-Taraxeren-3-ol, 3β-form,4-Hydroxy-E-cinnamoyl | -42.16 | 3,11,23-Trihydroxy-14-taraxeren-28-oic acid, (3β,11α)-form | -94.52 |
| 14-Taraxeren-3-ol, 3β-form,Tetracosanoyl | -87.37 | 3,11,23-Trihydroxy-14-taraxeren-28-oic acid | -100.68 |
| 14-Taraxerene-1,3-diol, (1α,3β)-form, 1-Ketone | 67.98 | 3,25-Epoxy-25-hydroxy-14-taraxeren-1-one, (3β,25α)-form | -73.52 |
| 14-Taraxerene-1,3-diol, (1α,3β)-form | -58.80 | 3,25-Epoxy-25-hydroxy-14-taraxeren-1-one | -71.35 |
| 14-Taraxerene-1,3-diol | -90.46 | 3,25-Epoxy-6,25-dihydroxy-14-taraxerene-1,16,21-trione, (3β,6α,25α)-form | -73.49 |
| 14-Taraxerene-3,11-diol, (3β,11α)-form, 11-O-(4-Hydroxy-E-cinnamoyl) | -85.21 | 3,25-Epoxy-6,25-dihydroxy-14-taraxerene-1,16,21-trione | -73.80 |
| 14-Taraxerene-3,11-diol, (3β,11α)-form | -75.78 | 3-Hydroxy-14-taraxeren-28-al, 3β-form | -87.11 |
| 14-Taraxerene-3,11-diol | -75.51 | 3-Hydroxy-14-taraxeren-28-al | -72.73 |
| 14-Taraxerene-3,22-diol, (3β,22β)-form, 3-Ac | -58.05 | 3-Hydroxy-14-taraxeren-28-oic acid, 3α-form, 3-O-(3,4-Dihydroxy-E-cinnamoyl) | -85.64 |
| 14-Taraxerene-3,22-diol, (3β,22β)-form, 3-O-(4-Hydroxy-Z-cinnamoyl) | -116.60 | 3-Hydroxy-14-taraxeren-28-oic acid, 3α-form, 3-O-(4-Hydroxy-E-cinnamoyl) | -92.96 |
| 14-Taraxerene-3,22-diol, (3β,22β)-form | -76.82 | 3-Hydroxy-14-taraxeren-28-oic acid, 3α-form, Ac | -118.50 |
| 14-Taraxerene-3,22-diol | -68.25 | 3-Hydroxy-14-taraxeren-28-oic acid, 3α-form | -101.41 |
| 14-Taraxerene-3,24-diol, 3β-form, Di-Ac | -101.77 | 3-Hydroxy-14-taraxeren-28-oic acid, 3β-form, 3-O-(4-Hydroxy-E-cinnamoyl) (**3-*O*-(*E*)-*p*-coumaroyl aleuritolic acid**) | -119.70 |
| 14-Taraxerene-3,24-diol, 3β-form | -96.92 | 3-Hydroxy-14-taraxeren-28-oic acid, 3β-form, 3-O-(4-Hydroxybenzoyl) | -43.79 |
| 14-Taraxerene-3,24-diol | -95.56 | 3-Hydroxy-14-taraxeren-28-oic acid, 3β-form, Ac | -110.71 |
| 14-Taraxerene-3,28-diol, 3α-form, 3-O-(3,4-Dihydroxy-E-cinnamoyl) (3-Caffeoylisomyricadiol) | -137.69 | 3-Hydroxy-14-taraxeren-28-oic acid, 3β-form | -101.49 |
| 14-Taraxerene-3,28-diol, 3α-form | -97.18 | 3-Hydroxy-14-taraxeren-28-oic acid | -101.60 |
| 14-Taraxerene-3,28-diol, 3β-form, 3-Ac | -96.29 | 6,7, 21,28-Diepoxy-14-taraxerene-3,28-diol(3α,6α,7α,21β,28α) | -89.82 |
| 14-Taraxerene-3,28-diol, 3β-form | -92.17 | 6,7, 21,28-Diepoxy-14-taraxerene-3,28-diol(3α,6α,7α,21β,28α)28-Ac | -74.65 |
| 14-Taraxerene-3,28-diol | -97.16 | 6,7, 21,28-Diepoxy-14-taraxerene-3,28-diol | -89.56 |
|  |  | Mudanpinoic acid | -76.40 |

**Supplementary Table 4:**  Docking energies of ursolic acid analogues for *T. cruzi* CYP51.

| **Compounds** | **Docking energies** | **Compounds** | **Docking energies** |
| --- | --- | --- | --- |
| 1,2,3,19,23-Pentahydroxy-12-ursen-28-oic acid, (1α,2α,3β,19α)-form | -105.09 | 2,3-Dihydroxy-12-ursen-28-oic acid, (2α,3β)-form, 3-(3,4-Dihydroxy-E-cinnamoyl) (**3-(*E*)-caffeoyl corosolic acid**) | -141.72 |
| 1,2,3,19,23-Pentahydroxy-12-ursen-28-oic acid, (1β,2α,3β,19α)-form | -79.10 | 2,3-Dihydroxy-12-ursen-28-oic acid, (2α,3β)-form, 3-(3,4-Dihydroxy-Z-cinnamoyl) (**3-(Z)-caffeoyl corosolic acid**) | -141.61 |
| 1,2,3,19,23-Pentahydroxy-12-ursen-28-oic acid | -85.06 | 2,3-Dihydroxy-12-ursen-28-oic acid, (2α,3β)-form, 3-(4-Hydroxy-3-methoxy-E-cinnamoyl) | -139.29 |
| 1,2,3,19-Tetrahydroxy-12-ursen-28-oic acid, (1α,2α,3β,19α)-form | -59.28 | 2,3-Dihydroxy-12-ursen-28-oic acid, (2α,3β)-form, 3-(4-Hydroxy-Z-cinnamoyl) (**3-(Z)-feruloyl corosolic acid**) | -134.97 |
| 1,2,3,19-Tetrahydroxy-12-ursen-28-oic acid, (1β,2α,3α,19α)-form | -88.00 | 2,3-Dihydroxy-12-ursen-28-oic acid, (2α,3β)-form, 3-Ac | -86.15 |
| 1,2,3,19-Tetrahydroxy-12-ursen-28-oic acid, (1β,2α,3β,19α)-form, 2-Ketone | -69.79 | 2,3-Dihydroxy-12-ursen-28-oic acid, (2α,3β)-form, 3-Me ether | -76.15 |
| 1,2,3,19-Tetrahydroxy-12-ursen-28-oic acid, (1β,2α,3β,19α)-form, Me ester | -85.64 | 2,3-Dihydroxy-12-ursen-28-oic acid, (2α,3β)-form, Di-Ac, Me ester | -99.24 |
| 1,2,3,19-Tetrahydroxy-12-ursen-28-oic acid, (1β,2α,3β,19α)-form | -65.32 | 2,3-Dihydroxy-12-ursen-28-oic acid, (2α,3β)-form, Di-Ac | -93.52 |
| 1,2,3,19-Tetrahydroxy-12-ursen-28-oic acid, (1β,2β,3β,19α)-form, Me ester | -87.99 | 2,3-Dihydroxy-12-ursen-28-oic acid, (2α,3β)-form, Me ester | -88.21 |
| 1,2,3,19-Tetrahydroxy-12-ursen-28-oic acid, (1β,2β,3β,19α)-form | -66.36 | 2,3-Dihydroxy-12-ursen-28-oic acid, (2α,3β)-form | -76.19 |
| 1,2,3,19-Tetrahydroxy-12-ursen-28-oic acid | -59.32 | 2,3-Dihydroxy-12-ursen-28-oic acid, (2α,3α)-form | -77.70 |
| 1,2,3,23-Tetrahydroxy-12-ursen-28-oic acid, (1β,2α,3β)-form | 112.49 | 2,3-Dihydroxy-12-ursen-28-oic acid, (2β,3β)-form | -32.52 |
| 1,2,3-Trihydroxy-12-ursen-28-oic acid, (1α,2α,3β)-form | -79.50 | 2,3-Dihydroxy-12-ursen-28-oic acid | -76.57 |
| 1,2,3-Trihydroxy-12-ursen-28-oic acid | -63.92 | 2,3-Dihydroxy-12-ursen-28-oic acid, (2α,3β)-form, 3-(4-Hydroxy-E-cinnamoyl) | -131.57 |
| 1,3-Dihydroxy-12-ursen-28-oic acid, (1α,3β)-form | -80.80 | 2,3-Dihydroxy-12-ursen-28-oic acid, (2α,3β)-form, 2-(4-Hydroxy-3-methoxy-E-cinnamoyl) (**2-(Z)-feruloyl corosolic acid**) | -136.11 |
| 1,3-Dihydroxy-12-ursen-28-oic acid, (1β,3β)-form | -80.90 | 2,3-Dihydroxy-12-ursen-28-oic acid, (2α,3β)-form, 2-(4-Hydroxy-E-cinnamoyl) | -125.14 |
| 1,3-Dihydroxy-12-ursen-28-oic acid | -80.86 | 2,3-Dihydroxy-12-ursen-28-oic acid, (2α,3β)-form, 2-(4-Hydroxy-Z-cinnamoyl) | -134.47 |
| 12-Ursene-1,3,19,23,28-pentol, (1α,3β,19α) form, 23,28-Dicarboxylic acid, 1-ketone, di-Me ester | -100.91 | 2,3-Dihydroxy-12-ursen-28-oic acid, (2α,3β)-form, 2-Ac | -94.70 |

**Supplementary Table 4 Cont’d:**  Docking energies of ursolic acid analogues for *T. cruzi* CYP51.

| **Compounds** | **Docking energies** | **Compounds** | **Docking energies** |
| --- | --- | --- | --- |
| 12-Ursene-1,3,19,23,28-pentol, (1α,3β,19α) form, 23,28-Dicarboxylic acid, 1-ketone | -109.92 | 2,3-Dihydroxy-12-ursen-28-oic acid, (2α,3β)-form, 2-Me ether, 3-(4-hydroxy-E-cinnamoyl) | -128.89 |
| 12-Ursene-1,3,19,23,28-pentol, (1α,3β,19α) form, 28-Dicarboxylic acid | -84.13 | 2,3-Dihydroxy-12-ursen-28-oic acid, (2α,3β)-form, 2-Me ether | -61.41 |
| 12-Ursene-3,11,28-triol, (3β,11α) form, 28-Carboxylic acid, 11-Me ether, 3-Ac, Me ester | -101.80 | 2,3-Dihydroxy-23-oxo-12-ursen-28-oic acid, (2α,3β)-form | -97.46 |
| 12-Ursene-3,11,28-triol, (3β,11α) form, 28-Carboxylic acid, 11-Me ether | -57.92 | 2,3-Dihydroxy-23-oxo-12-ursen-28-oic acid, (2β,3β)-form | -96.68 |
| 12-Ursene-3,11,28-triol, (3β,11α) form, 28-Carboxylic acid, 11-hydroperoxide, 3-Ac | -99.22 | 2,3-Dihydroxy-23-oxo-12-ursen-28-oic acid | -97.76 |
| 12-Ursene-3,11,28-triol, (3β,11α) form, 28-Carboxylic acid, 11-ketone, 3-Ac | -38.75 | 3,19,21,23-Tetrahydroxy-12-taraxasten-28-oic acid, (3α,19β,21α)-form | -105.80 |
| 12-Ursene-3,11,28-triol, (3β,11α) form, 28-Carboxylic acid, 11-ketone, 3-benzoyl | -100.51 | 3,19,21,23-Tetrahydroxy-12-taraxasten-28-oic acid | -105.81 |
| 2-Ursene-3,11,28-triol, (3β,11α) form, 28-Carboxylic acid, 11-ketone | -98.65 | 3,19,21,23-Tetrahydroxy-12-ursen-28-oic acid, (3β,19α,21β)-form | -44.43 |
| 12-Ursene-3,11,28-triol, (3β,11α) form, 28-Carboxylic acid | -90.72 | 3,19,21,23-Tetrahydroxy-12-ursen-28-oic acid | -43.66 |
| 12-Ursene-3,21,28-triol, (3β,21α) form, 28-Carboxylic acid | -69.66 | 3,19,23,24-Tetrahydroxy-12-ursen-28-oic acid, (3α,19α)-form | -64.28 |
| 12-Ursene-3,21,28-triol, (3β,21β) form, 28-Carboxylic acid | -69.23 | 3,19,23,24-Tetrahydroxy-12-ursen-28-oic acid, (3β,19α)-form, 23-Aldehyde | -96.95 |
| 2,25-Epoxy-2,3,19-trihydroxy-12-ursen-28-oic acid, (2α,3β,19α)-form | -99.75 | 3,19,23,24-Tetrahydroxy-12-ursen-28-oic acid, (3β,19α)-form, 23-Carboxylic acid | -88.31 |
| 2,25-Epoxy-2,3,19-trihydroxy-12-ursen-28-oic acid | -100.65 | 3,19,23,24-Tetrahydroxy-12-ursen-28-oic acid, (3β,19α)-form, 24-Carboxylic acid | -102.01 |
| 2,3,11,19-Tetrahydroxy-12-ursen-28-oic acid, (2α,3α,11α,19α)-form, 11-Ketone | -86.06 | 3,19,23,24-Tetrahydroxy-12-ursen-28-oic acid, (3β,19α)-form | -93.37 |
| 2,3,11,19-Tetrahydroxy-12-ursen-28-oic acid, (2α,3α,11α,19α)-form | -81.46 | 3,19,23,24-Tetrahydroxy-12-ursen-28-oic acid, (3β,19α,20αH)-form, 23-Carboxylic acid, 23-Me ester | -81.29 |
| 2,3,11,19-Tetrahydroxy-12-ursen-28-oic acid, (2α,3β,11α,19α)-form, 2,11-Di-Ac | 38.76 | 3,19,23,24-Tetrahydroxy-12-ursen-28-oic acid, (3β,19α,20αH)-form, 23-Carboxylic acid | -64.52 |
| 2,3,11,19-Tetrahydroxy-12-ursen-28-oic acid, (2α,3β,11α,19α)-form | -81.38 | 3,19,23,24-Tetrahydroxy-12-ursen-28-oic acid, (3β,19α,20αH)-form | -67.24 |
| 2,3,11,19-Tetrahydroxy-12-ursen-28-oic acid | -65.10 | 3,19,23,24-Tetrahydroxy-12-ursen-28-oic acid | -68.73 |
| 2,3,11,23-Tetrahydroxy-12-ursen-28-oic acid, (2α,3β,11α)-form | -95.51 | 3,19,23,30-Tetrahydroxy-12-ursen-28-oic acid, (3β,19α)-form | -82.14 |

**Supplementary Table 4 Cont’d:**  Docking energies of ursolic acid analogues for *T. cruzi* CYP51.

| **Compounds** | **Docking energies** | **Compounds** | **Docking energies** |
| --- | --- | --- | --- |
| 2,3,11,23-Tetrahydroxy-12-ursen-28-oic acid, (2α,3β,11β)-form, 11-Ketone, Me ester | -119.79 | 3,19,23,30-Tetrahydroxy-12-ursen-28-oic acid | -63.72 |
| 2,3,11,23-Tetrahydroxy-12-ursen-28-oic acid, (2α,3β,11β)-form, 11-Ketone | -115.47 | 3,19,23-Trihydroxy-12-ursen-28-oic acid, (3β,19α)-form, 23-Aldehyde | -38.04 |
| 2,3,11,23-Tetrahydroxy-12-ursen-28-oic acid, (2α,3β,11β)-form, 11-Me ether | -102.03 | 3,19,23-Trihydroxy-12-ursen-28-oic acid, (3β,19α)-form, 3,23-Disulfate | -92.20 |
| 2,3,11,23-Tetrahydroxy-12-ursen-28-oic acid, (2α,3β,11β)-form | -95.53 | 3,19,23-Trihydroxy-12-ursen-28-oic acid, (3β,19α)-form, 3-O-(4-Hydroxy-E-cinnamoyl) | -135.58 |
| 2,3,11,23-Tetrahydroxy-12-ursen-28-oic acid | -95.56 | 3,19,23-Trihydroxy-12-ursen-28-oic acid, (3β,19α)-form | -85.72 |
| 2,3,16,23-Tetrahydroxy-12-ursen-28-oic acid, (2ξ,3β,16α)-form | -84.63 | 3,19,23-Trihydroxy-12-ursen-28-oic acid | -85.89 |
| 2,3,16,23-Tetrahydroxy-12-ursen-28-oic acid | -91.27 | 3,19,24-Trihydroxy-12-ursen-28-oic acid, (3α,19α)-form | -87.44 |
| 2,3,19,22-Tetrahydroxy-12-ursen-28-oic acid, (2α,3α,19α,22α)-form | -94.28 | 3,19,24-Trihydroxy-12-ursen-28-oic acid, (3β,19α)-form, 24-O-(3-Hydroxy-4-methoxycinnamoyl) | -122.77 |
| 2,3,19,22-Tetrahydroxy-12-ursen-28-oic acid | -92.96 | 3,19,24-Trihydroxy-12-ursen-28-oic acid, (3β,19α)-form | -86.89 |
| 2,3,19,23,24-Pentahydroxy-12-ursen-28-oic acid, (2α,3α,19α)-form | -79.09 | 3,19,24-Trihydroxy-12-ursen-28-oic acid, (3β,19α,20αH)-form | -91.07 |
| 2,3,19,23,24-Pentahydroxy-12-ursen-28-oic acid, (2α,3β,19α)-form | -75.38 | 3,19,24-Trihydroxy-12-ursen-28-oic acid | -84.25 |
| 2,3,19,23,24-Pentahydroxy-12-ursen-28-oic acid | -80.99 | 3,19-Dihydroxy-12-ursen-28-oic acid, (3α,19α)-form, 3-Ac | -54.39 |
| 2,3,19,23-Tetrahydroxy-12-ursen-28-oic acid, (2α,3α)-form | -100.49 | 3,19-Dihydroxy-12-ursen-28-oic acid, (3α,19α)-form | -39.70 |
| 2,3,19,23-Tetrahydroxy-12-ursen-28-oic acid, (2α,3β)-form, 23-O-(4-Hydroxy-E-cinnamoyl) | -135.50 | 3,19-Dihydroxy-12-ursen-28-oic acid, (3β,19α)-form, Me ester | -59.84 |
| 2,3,19,23-Tetrahydroxy-12-ursen-28-oic acid, (2α,3β)-form, 23-O-(4-Hydroxy-Z-cinnamoyl) | -132.36 | 3,19-Dihydroxy-12-ursen-28-oic acid, (3β,19α)-form | -39.98 |
| 2,3,19,23-Tetrahydroxy-12-ursen-28-oic acid, (2α,3β)-form, 23-Sulfate | -114.78 | 3,19-Dihydroxy-12-ursen-28-oic acid, (3β,19α,20αH)-form | -90.44 |
| 2,3,19,23-Tetrahydroxy-12-ursen-28-oic acid, (2α,3β)-form, 3,23-Disulfate | -110.62 | 3,19-Dihydroxy-12-ursen-28-oic acid | -39.92 |
| 2,3,19,23-Tetrahydroxy-12-ursen-28-oic acid, (2α,3β)-form, Me ester | 79.09 | 3,19-Dihydroxy-12-ursene-23,28-dioic acid, (3α,19α,20β)-form | -67.50 |
| 2,3,19,23-Tetrahydroxy-12-ursen-28-oic acid, (2α,3β)-form | -75.55 | 3,19-Dihydroxy-12-ursene-23,28-dioic acid, (3β,19α,20α)-form | -64.67 |
| 2,3,19,23-Tetrahydroxy-12-ursen-28-oic acid, (2α,3β,20β)-form | -100.08 | 3,19-Dihydroxy-12-ursene-23,28-dioic acid, (3β,19α,20β)-form | -79.97 |

**Supplementary Table 4 Cont’d:**  Docking energies of ursolic acid analogues for *T. cruzi* CYP51.

| **Compounds** | **Docking energies** | **Compounds** | **Docking energies** |
| --- | --- | --- | --- |
| 2,3,19,23-Tetrahydroxy-12-ursen-28-oic acid, (2β,3α)-form | -98.39 | 3,19-Dihydroxy-12-ursene-23,28-dioic acid | -64.68 |
| 2,3,19,23-Tetrahydroxy-12-ursen-28-oic acid | -104.29 | 3,19-Dihydroxy-12-ursene-24,28-dioic acid, (3α,19α)-form | -78.35 |
| 2,3,19,24-Tetrahydroxy-12-ursen-28-oic acid, (2α,3α)-form | -93.13 | 3,19-Dihydroxy-12-ursene-24,28-dioic acid, (3β,19α)-form | -67.57 |
| 2,3,19,24-Tetrahydroxy-12-ursen-28-oic acid, (2α,3β)-form, 24-Aldehyde | -96.02 | 3,19-Dihydroxy-12-ursene-24,28-dioic acid, (3β,19α,20β)-form | -88.81 |
| 2,3,19,24-Tetrahydroxy-12-ursen-28-oic acid, (2α,3β)-form, 24-Butyl ether | -85.13 | 3,19-Dihydroxy-12-ursene-24,28-dioic acid | -77.06 |
| 2,3,19,24-Tetrahydroxy-12-ursen-28-oic acid, (2α,3β)-form | -93.24 | 3,19-Dihydroxy-24-oxo-12-ursen-28-oic acid, (3β,19α)-form | -89.20 |
| 2,3,19,24-Tetrahydroxy-12-ursen-28-oic acid | -93.20 | 3,19-Dihydroxy-24-oxo-12-ursen-28-oic acid | -87.24 |
| 2,3,19,24-Tetrahydroxy-12-ursene-23,28-dioic acid, (2α,3β,19α)-form | -102.40 | 3,20-Dihydroxy-12-ursen-28-oic acid, 3β-form, 20-Ac | -98.06 |
| 2,3,19,24-Tetrahydroxy-12-ursene-23,28-dioic acid | -105.46 | 3,20-Dihydroxy-12-ursen-28-oic acid, 3β-form, 3-(4-Hydroxy-Z-cinnamoyl) | -121.27 |
| 2,3,19,30-Tetrahydroxy-12-ursene-24,28-dioic acid, (2α,3β,19α)-form | -98.49 | 3,20-Dihydroxy-12-ursen-28-oic acid, 3β-form | -85.21 |
| 2,3,19,30-Tetrahydroxy-12-ursene-24,28-dioic acid | -106.66 | 3,20-Dihydroxy-12-ursen-28-oic acid | -85.24 |
| 2,3,19-Trihydroxy-12-ursen-28-oic acid, (2α,3α,19α)-form, 2,3-O-Isopropylidene | -90.88 | 3,21,23-Trihydroxy-12-ursen-28-oic acid, (3β,21α)-form | -93.36 |
| 2,3,19-Trihydroxy-12-ursen-28-oic acid, (2α,3α,19α)-form, 2-Ac | -82.82 | 3,21,23-Trihydroxy-12-ursen-28-oic acid | -92.04 |
| 2,3,19-Trihydroxy-12-ursen-28-oic acid, (2α,3β,19α)-form, 3-O-(4-Hydroxy-3-methoxy-E-cinnamoyl) | -123.91 | 3,22-Dihydroxy-12-ursen-28-oic acid, (3β,22α)-form | -109.29 |
| 2,3,19-Trihydroxy-12-ursen-28-oic acid, (2α,3β,19α)-form, 3-O-(4-Hydroxy-E-cinnamoyl) | -126.06 | 3,22-Dihydroxy-12-ursen-28-oic acid | -101.81 |
| 2,3,19-Trihydroxy-12-ursen-28-oic acid, (2α,3β,19α)-form, 3-O-(4-Hydroxy-Z-cinnamoyl) | -113.81 | 3,23-Dihydroxy-12-ursen-28-oic acid, 3β-form | -76.54 |
| 2,3,19-Trihydroxy-12-ursen-28-oic acid, (2α,3β,19α)-form, 3-O-(4-Hydroxybenzoyl) | -105.44 | 3,23-Dihydroxy-12-ursen-28-oic acid | -78.37 |
| 2,3,19-Trihydroxy-12-ursen-28-oic acid, (2α,3β,19α)-form | -70.15 | 3,24-Dihydroxy-12-ursen-28-oic acid, 3α-form | -64.70 |
| 2,3,19-Trihydroxy-12-ursen-28-oic acid, (2α,3β,19β)-form, 28→19-Lactone, 2-Ac | -83.33 | 3,24-Dihydroxy-12-ursen-28-oic acid, 3β-form, 24-(4-Hydroxycinnamoyl)(E-) | -120.37 |
| 2,3,19-Trihydroxy-12-ursen-28-oic acid, (2α,3β,19β)-form | -62.87 | 3,24-Dihydroxy-12-ursen-28-oic acid, 3β-form, 24-O-(4-Hydroxy-3-methoxy-E-cinnamoyl) | -127.44 |

**Supplementary Table 4 Cont’d:**  Docking energies of ursolic acid analogues for *T. cruzi* CYP51.

| **Compounds** | **Docking energies** | **Compounds** | **Docking energies** |
| --- | --- | --- | --- |
| 2,3,19-Trihydroxy-12-ursen-28-oic acid, (2β,3α)-form | -71.71 | 3,24-Dihydroxy-12-ursen-28-oic acid, 3β-form, 24-O-(4-Hydroxy-3-methoxy-Z-cinnamoyl) | -122.66 |
| 2,3,19-Trihydroxy-12-ursen-28-oic acid, (2β,3β,19α)-form, 2,3-Isopropylidene | -92.70 | 3,24-Dihydroxy-12-ursen-28-oic acid, 3β-form | -64.15 |
| 2,3,19-Trihydroxy-12-ursen-28-oic acid, (2β,3β,19α)-form | -72.87 | 3,24-Dihydroxy-12-ursen-28-oic acid | -69.84 |
| 2,3,19-Trihydroxy-12-ursen-28-oic acid, (2α,3α,19α)-form, 2-Ketone | -68.00 | 3,25-Epoxy-3,22-dihydroxy-12-ursen-28-oic acid, 22β-form, 3-Et ether, 22-angeloyl | -124.07 |
| 2,3,19-Trihydroxy-12-ursen-28-oic acid | -72.75 | 3,25-Epoxy-3,22-dihydroxy-12-ursen-28-oic acid, 22β-form, 3-Me ether, 22-angeloyl | -120.19 |
| 2,3,19-Trihydroxy-12-ursen-28-oic acid, (2α,3α,19α)-form, 3-O-(4-Hydroxy-3-methoxy-E-cinnamoyl) | -94.97 | 3,25-Epoxy-3,22-dihydroxy-12-ursen-28-oic acid | 64.24 |
| 2,3,19-Trihydroxy-12-ursen-28-oic acid, (2α,3α,19α)-form, Me ester | -64.57 | 3,25-Epoxy-3-hydroxy-12-ursen-28-oic acid, 3-Et ether | -92.83 |
| 2,3,19-Trihydroxy-12-ursen-28-oic acid, (2α,3α,19α)-form | -76.04 | 3,25-Epoxy-3-hydroxy-12-ursen-28-oic acid | 41.63 |
| 2,3,19-Trihydroxy-12-ursen-28-oic acid, (2α,3β,19α)-form, 2-Ac | -97.28 | 3,27-Dihydroxy-12-ursen-28-oic acid, (3β,20β)-form, 27-O-(4-Hydroxy-E-cinnamoyl) | -51.06 |
| 2,3,19-Trihydroxy-12-ursen-28-oic acid, (2α,3β,19α)-form, 2-Ketone | -69.54 | 3,27-Dihydroxy-12-ursen-28-oic acid, (3β,20β)-form | -98.94 |
| 2,3,19-Trihydroxy-12-ursen-28-oic acid, (2α,3β,19α)-form, 3-Ac | -79.66 | 3,27-Dihydroxy-12-ursen-28-oic acid, 3β-form, 27-(4-Hydroxy-3-methoxycinnamoyl)(E-) | -103.99 |
| 2,3,19-Trihydroxy-12-ursen-28-oic acid, (2α,3β,19α)-form, 3-O-(3,4-Dihydroxy-E-cinnamoyl) | -116.69 | 3,27-Dihydroxy-12-ursen-28-oic acid, 3β-form, 27-(4-Hydroxy-3-methoxycinnamoyl)(Z-) | -63.64 |
| 2,3,19-Trihydroxy-12-ursene-23,28-dioic acid, (2α,3β,19α)-form | -36.71 | 3,27-Dihydroxy-12-ursen-28-oic acid, 3β-form, 27-(4-Hydroxycinnamoyl)(E-) | -78.16 |
| 2,3,19-Trihydroxy-12-ursene-23,28-dioic acid | -36.79 | 3,27-Dihydroxy-12-ursen-28-oic acid, 3β-form, 27-(4-Hydroxycinnamoyl)(Z-) | -47.95 |
| 2,3,19-Trihydroxy-12-ursene-24,28-dioic acid, (2α,3α,19α)-form | -77.28 | 3,27-Dihydroxy-12-ursen-28-oic acid, 3β-form, 27-Aldehyde | -91.41 |
| 2,3,19-Trihydroxy-12-ursene-24,28-dioic acid, (2α,3β,19α)-form | -39.90 | 3,27-Dihydroxy-12-ursen-28-oic acid, 3β-form, 27-O-(4-Hydroxyphenyl) | -75.69 |
| 2,3,19-Trihydroxy-12-ursene-24,28-dioic acid | -42.71 | 3,27-Dihydroxy-12-ursen-28-oic acid, 3β-form | -99.69 |
| 2,3,19-Trihydroxy-23-oxo-12-ursen-28-oic acid, (2α,3α)-form | -94.64 | 3,27-Dihydroxy-12-ursen-28-oic acid | -36.78 |
| 2,3,19-Trihydroxy-23-oxo-12-ursen-28-oic acid, (2α,3β)-form | -94.68 | 3,30-Dihydroxy-12-ursen-28-oic acid, 3β-form, 3-O-(4-Hydroxycinnamoyl) | -130.62 |

**Supplementary Table 4 Cont’d:**  Docking energies of ursolic acid analogues for *T. cruzi* CYP51.

| **Compounds** | **Docking energies** | **Compounds** | **Docking energies** |
| --- | --- | --- | --- |
| 2,3,19-Trihydroxy-23-oxo-12-ursen-28-oic acid | -94.73 | 3,30-Dihydroxy-12-ursen-28-oic acid, 3β-form, 30-O-(4-Hydroxycinnamoyl) | -126.79 |
| 2,3,20,23,24,30-Hexahydroxy-12-ursen-28-oic acid, (2β,3α,20β)-form | -89.88 | 3,30-Dihydroxy-12-ursen-28-oic acid, (3β,20αH)-form | -74.42 |
| 2,3,20,23,24,30-Hexahydroxy-12-ursen-28-oic acid | -95.76 | 3,30-Dihydroxy-12-ursen-28-oic acid, 3β-form | -74.92 |
| 2,3,20-Trihydroxy-12-ursene-24,28-dioic acid, (2α,3β,20β)-form, Di-Me ester | -75.78 | 3,30-Dihydroxy-12-ursen-28-oic acid | -74.03 |
| 2,3,20-Trihydroxy-12-ursene-24,28-dioic acid, (2α,3β,20β)-form | -81.14 | 3,5,6,24-Tetrahydroxy-12-ursen-28-oic acid, (3α,5α,6β)-for | -75.76 |
| 2,3,20-Trihydroxy-12-ursene-24,28-dioic acid | -80.27 | 3,5,6,24-Tetrahydroxy-12-ursen-28-oic acid | -84.98 |
| 2,3,23,24-Tetrahydroxy-12-ursen-28-oic acid, (2α,3α)-form | -27.80 | 3,6,19,23-Tetrahydroxy-12-ursen-28-oic acid, (3β,6α,19α)-form | -93.25 |
| 2,3,23,24-Tetrahydroxy-12-ursen-28-oic acid, (2α,3β)-form | -84.84 | 3,6,19,23-Tetrahydroxy-12-ursen-28-oic acid, (3β,6β,19α)-form, 23-Aldehyde | -94.71 |
| 2,3,23,24-Tetrahydroxy-12-ursen-28-oic acid, 23-Aldehyde | -95.61 | 3,6,19,23-Tetrahydroxy-12-ursen-28-oic acid, (3β,6β,19α)-form, 23-Carboxylic acid | -97.14 |
| 2,3,23,24-Tetrahydroxy-12-ursen-28-oic acid, 23-Carboxylic acid | -76.64 | 3,6,19,23-Tetrahydroxy-12-ursen-28-oic acid, (3β,6β,19α)-form, 6-Ketone, 23-aldehyde | -87.06 |
| 2,3,23,24-Tetrahydroxy-12-ursen-28-oic acid | -102.81 | 3,6,19,23-Tetrahydroxy-12-ursen-28-oic acid, (3β,6β,19α)-form, 6-Ketone | -88.98 |
| 2,3,23,30-Tetrahydroxy-12-ursen-28-oic acid, (2α,3β)-form | -99.07 | 3,6,19,23-Tetrahydroxy-12-ursen-28-oic acid, (3β,6β,19α)-form | -82.06 |
| 2,3,23,30-Tetrahydroxy-12-ursen-28-oic acid | -12.52 | 3,6,19,23-Tetrahydroxy-12-ursen-28-oic acid | -85.16 |
| 2,3,23-Trihydroxy-12-ursen-28-oic acid, (2α,3α)-form | -93.84 | 3,6,19-Trihydroxy-12-ursen-28-oic acid, (3β,6α,19α)-form | -71.49 |
| 2,3,23-Trihydroxy-12-ursen-28-oic acid, (2α,3β)-form, 2,23-Di-Ac | -91.90 | 3,6,19-Trihydroxy-12-ursen-28-oic acid, (3β,6β,19α)-form | -73.10 |
| 2,3,23-Trihydroxy-12-ursen-28-oic acid, (2α,3β)-form, 2-Ac | -72.94 | 3,6,19-Trihydroxy-12-ursen-28-oic acid, (3β,6β,19α)-form, Me ester | -77.89 |
| 2,3,23-Trihydroxy-12-ursen-28-oic acid, (2α,3β)-form, 3-O-(4-Hydroxy-E-cinnamoyl) | -112.41 | 3,6,19-Trihydroxy-12-ursen-28-oic acid, (3β,6β,19α)-form | -81.40 |
| 2,3,23-Trihydroxy-12-ursen-28-oic acid, (2α,3β)-form | -93.92 | 3,6,19-Trihydroxy-12-ursen-28-oic acid | -71.81 |
| 2,3,23-Trihydroxy-12-ursen-28-oic acid, (2β,3β)-form | -93.80 | 3,6,23,30-Tetrahydroxy-12-ursen-28-oic acid, (3β,6α)-form, 23-(4-Hydroxy-E-cinnamoyl) | -101.76 |
| 2,3,23-Trihydroxy-12-ursen-28-oic acid | -93.80 | 3,6,23,30-Tetrahydroxy-12-ursen-28-oic acid, (3β,6α)-form | -73.36 |
| 2,3,24,30-Tetrahydroxy-12-ursen-28-oic acid, (2α,3β,20β)-form, 28→30 Lactone | -102.58 | 3,6,23,30-Tetrahydroxy-12-ursen-28-oic acid | -79.28 |

**Supplementary Table 4 Cont’d:**  Docking energies of ursolic acid analogues for *T. cruzi* CYP51.

| **Compounds** | **Docking energies** | **Compounds** | **Docking energies** |
| --- | --- | --- | --- |
| 2,3,24,30-Tetrahydroxy-12-ursen-28-oic acid, (2α,3β,20β)-form | -94.46 | 3,6,23-Trihydroxy-12-ursen-28-oic acid, (3β,6β)-form | -90.26 |
| 2,3,24,30-Tetrahydroxy-12-ursen-28-oic acid | -108.22 | 3,6,23-Trihydroxy-12-ursen-28-oic acid | -103.23 |
| 2,3,24-Trihydroxy-12-ursen-28-oic acid, (2α,3α)-form, 24-Ac | -88.48 | 3,6-Dihydroxy-12-ursen-28-oic acid, (3β,6β)-form, 6-Ketone, 3-benzoyl | -109.54 |
| 2,3,24-Trihydroxy-12-ursen-28-oic acid, (2α,3α)-form, 24-Aldehyde | -81.37 | 3,6-Dihydroxy-12-ursen-28-oic acid, (3β,6β)-form | -85.98 |
| 2,3,24-Trihydroxy-12-ursen-28-oic acid, (2α,3α)-form, 24-O-(4-Hydroxy-3-methoxy-E-cinnamoyl) | -123.13 | 3,6-Dihydroxy-12-ursen-28-oic acid | -106.78 |
| 2,3,24-Trihydroxy-12-ursen-28-oic acid, (2α,3α)-form | -81.70 | 3,7,24-Trihydroxy-12-ursen-28-oic acid, (3β,7β)-form | -106.76 |
| 2,3,24-Trihydroxy-12-ursen-28-oic acid, (2α,3β)-form, 24-O-(4-Hydroxy-E-cinnamoyl) | -103.29 | 3,7,24-Trihydroxy-12-ursen-28-oic acid | -96.92 |
| 2,3,24-Trihydroxy-12-ursen-28-oic acid, (2α,3β)-form, 24-O-(4-Hydroxy-Z-cinnamoyl) | -54.48 | 3,7-Dihydroxy-12-ursen-28-oic acid, (3β,7α)-form | -79.39 |
| 2,3,24-Trihydroxy-12-ursen-28-oic acid, (2α,3β)-form, 3-O-(4-Hydroxycinnamoyl) | -105.92 | 3,7-Dihydroxy-12-ursen-28-oic acid | -56.42 |
| 2,3,24-Trihydroxy-12-ursen-28-oic acid, (2α,3β)-form | -81.67 | 3-Hydroxy-12-taraxasten-28-oic acid, (3β,18β)-form | -84.59 |
| 2,3,24-Trihydroxy-12-ursen-28-oic acid, (2β,3α)-form | -81.49 | 3-Hydroxy-12-taraxasten-28-oic acid | -71.99 |
| 2,3,24-Trihydroxy-12-ursen-28-oic acid | -81.45 | 3-Hydroxy-12-ursen-28-oic acid, (3β,18α)-form | -87.07 |
| 2,3,30-Trihydroxy-12-ursen-28-oic acid, (2α,3β,20β)-form, 28→30 Lactone | -97.84 | 3-Hydroxy-12-ursen-28-oic acid, 3α-form, 3-O-(4-Acetylphenyl) | -125.92 |
| 2,3,30-Trihydroxy-12-ursen-28-oic acid, (2α,3β,20β)-form | -94.13 | -Hydroxy-12-ursen-28-oic acid, 3β-form, Ac | -96.92 |
| 2,3,30-Trihydroxy-12-ursen-28-oic acid | -94.75 | 3-Hydroxy-12-ursen-28-oic acid, 3β-form, Formyl | -96.88 |
| 2,3,6,19,23-Pentahydroxy-12-ursen-28-oic acid, (2α,3β,6β,19α)-form | -84.63 | 3-Hydroxy-12-ursen-28-oic acid, 3β-form, Malonyl ester | -85.17 |
| 2,3,6,19,23-Pentahydroxy-12-ursen-28-oic acid | -84.67 | 3-Hydroxy-12-ursen-28-oic acid, 3β-form, Me ester | -79.48 |
| 2,3,6,19-Tetrahydroxy-12-ursen-28-oic acid, (2α,3β,6β,19α)-form | -101.27 | 3-Hydroxy-12-ursen-28-oic acid, 3β-form | -83.03 |
| 2,3,6,19-Tetrahydroxy-12-ursen-28-oic acid | -101.30 | 3-Hydroxy-12-ursen-28-oic acid | -83.04 |
| 2,3,6,20,23,30-Hexahydroxy-12-ursen-28-oic acid, (2β,3α,6α,20β)-form | -62.46 | 3-Hydroxy-12-ursen-28-oic acid, 3α-form | -82.98 |
| 2,3,6,20,23,30-Hexahydroxy-12-ursen-28-oic acid | -97.16 | 3-Hydroxy-12-ursen-28-oic acid, 3β-form, 3-O-(4-Acetylphenyl) | -127.15 |

**Supplementary Table 4 Cont’d:**  Docking energies of ursolic acid analogues for *T. cruzi* CYP51.

| **Compounds** | **Docking energies** | **Compounds** | **Docking energies** |
| --- | --- | --- | --- |
| 2,3,6,23-Tetrahydroxy-12-ursen-28-oic acid, (2α,3β,6β)-form | -57.05 | 3-Hydroxy-12-ursen-28-oic acid, 3β-form, 3-O-(4-Hydroxy-Z-cinnamoyl) | -118.58 |
| 2,3,6,23-Tetrahydroxy-12-ursen-28-oic acid | -83.91 | 3-Hydroxy-12-ursen-28-oic acid, 3β-form, 3-O-(4-Hydroxycinnamoyl) | -115.85 |
| 2,3,6-Trihydroxy-12-ursen-28-oic acid, (2α,3β,6β)-form | -82.62 | 3-Hydroxy-12-ursen-28-oic acid, 3β-form, 3-O-(4-Methoxy-Z-cinnamoyl), Me ester | -101.41 |
| 2,3,6-Trihydroxy-12-ursen-28-oic acid | -93.57 | 3-Hydroxy-12-ursen-28-oic acid, 3β-form, 3-O-Docosanoyl | -126.55 |
| 2,3,7,19,23-Pentahydroxy-12-ursen-28-oic acid, (2α,3β,7α)-form | -67.19 | 3-Hydroxy-12-ursen-28-oic acid, 3β-form, Ac, 2-methoxybenzyl ester | -72.68 |
| 2,3,7,19,23-Pentahydroxy-12-ursen-28-oic acid | -83.12 | 3-Hydroxy-12-ursen-28-oic acid, 3β-form, Ac, 4-methoxybenzyl ester | -109.70 |
| 2,3,7,19-Tetrahydroxy-12-ursen-28-oic acid, (2α,3α,7β)-form | -77.97 | 3-Hydroxy-12-ursene-23,28-dioic acid, 3β-form | -77.91 |
| 2,3,7,19-Tetrahydroxy-12-ursen-28-oic acid, (2α,3β,7α)-form | -70.45 | 3-Hydroxy-12-ursene-23,28-dioic acid | -77.98 |
| 2,3,7,19-Tetrahydroxy-12-ursen-28-oic acid | -77.95 | 3-Hydroxy-12-ursene-27,28-dioic acid, 3β-form, Ac | -96.24 |
| 2,3,9-Trihydroxy-12-ursen-28-oic acid, (2α,3β,9α)-form | -20.27 | 3-Hydroxy-12-ursene-27,28-dioic acid, 3β-form | -86.20 |
| ,3,9-Trihydroxy-12-ursen-28-oic acid | -101.49 | 3-Hydroxy-12-ursene-27,28-dioic acid | -86.23 |
| 2,3-Dihydroxy-12,18-ursadien-28-oic acid, 18α,19α-Epoxide | -81.53 | 3-Hydroxy-12-ursene-27,28-dioic acid | -86.22 |
| 2,3-Dihydroxy-12-ursen-28-oic acid, (2α,3α)-form, 2-Me ether | -72.28 | Coreanogenic acid | -77.43 |
| 2,3-Dihydroxy-12-ursen-28-oic acid, (2α,3α)-form, 3-(4-Hydroxy-3-methoxy-E-cinnamoyl) (**3-(*E*)-feruloyl 2,3-Dihydroxy-12-ursen-28-oic acid**) | -135.71 | Sanguidiogenin E | -51.03 |

**Supplementary Table 5:** Docking energies of *epi*-Oleanolic acid analogues for *L. infantum* and *T. cruzi* CYP51.

| **Compounds** | ***T. cruzi* CYP51** | ***L. infantum* CYP51** | **Compounds** | ***T. cruzi* CYP51** | ***L. infantum* CYP51** |
| --- | --- | --- | --- | --- | --- |
| 1,2,3,19,23-Pentahydroxy-12-oleanen-28-oic acid, (1β,2α,3β,19α)-form | -55.33 | -67.47 | 2,3,23-Trihydroxy-11-oxo-12-oleanen-28-oic acid | -102.16 | -108.07 |
| 1,2,3,19,23-Pentahydroxy-12-oleanen-28-oic acid | -79.88 | -89.99 | 2,3,23-Trihydroxy-12-oleanen-28-oic acid, (2α,3α)-form, 23-O-(4-Hydroxy-E-cinnamoyl) | -116.05 | -101.43 |
| 1,2,3,23-Tetrahydroxy-12-oleanen-28-oic acid, (1β,2α,3β)-form | -101.50 | -87.07 | 2,3,23-Trihydroxy-12-oleanen-28-oic acid, (2α,3α)-form, 23-O-(4-Hydroxy-Z-cinnamoyl) | -115.58 | -100.52 |

**Supplementary Table 5 Cont’d:** Docking energies of *epi*-Oleanolic acid analogues for *L. infantum* and *T. cruzi* CYP51.

| **Compounds** | ***T. cruzi* CYP51** | ***L. infantum* CYP51** | **Compounds** | ***T. cruzi* CYP51** | ***L. infantum* CYP51** |
| --- | --- | --- | --- | --- | --- |
| 1,2,3,23-Tetrahydroxy-12-oleanen-28-oic acid | -97.53 | -94.09 | 2,3,23-Trihydroxy-12-oleanen-28-oic acid, (2α,3β)-form, 3-O-(3,4-Dihydroxy-E-cinnamoyl) | -108.94 | -85.12 |
| 1,2,3,24-Tetrahydroxy-12-oleanen-28-oic acid, (1α,2α,3β)-form | -72.57 | -67.93 | 2,3,23-Trihydroxy-12-oleanen-28-oic acid, (2α,3β)-form, 3-O-(3,4-Dihydroxy-Z-cinnamoyl) | -108.46 | -81.13 |
| 1,2,3,24-Tetrahydroxy-12-oleanen-28-oic acid, (1β,2α,3α)-form | -101.47 | -27.72 | 2,3,23-Trihydroxy-12-oleanen-28-oic acid, (2α,3β)-form, 3-O-(4-Hydroxy-E-cinnamoyl) | -118.87 | -120.99 |
| 1,2,3,24-Tetrahydroxy-12-oleanen-28-oic acid | -101.10 | -69.04 | 2,3,23-Trihydroxy-12-oleanen-28-oic acid, (2α,3β)-form, 3-O-(4-Hydroxy-Z-cinnamoyl) | -86.06 | -126.41 |
| 1,2,3-Trihydroxy-12-oleanen-28-oic acid, (1α,2α,3β)-form | -90.46 | -81.37 | 2,3,23-Trihydroxy-12-oleanen-28-oic acid, (2α,3β)-form, Me ester | -89.66 | -107.73 |
| 1,2,3-Trihydroxy-12-oleanen-28-oic acid | -95.83 | -57.85 | 2,3,23-Trihydroxy-12-oleanen-28-oic acid, (2α,3β)-form, Tri-Ac, Me ester | -42.38 | -79.76 |
| 1,3,16,23-Tetrahydroxy-12-oleanen-28-oic acid, (1α,3β,16α)-form | -90.73 | -99.38 | 2,3,23-Trihydroxy-12-oleanen-28-oic acid, (2α,3β)-form | -83.91 | -104.21 |
| 1,3,16,23-Tetrahydroxy-12-oleanen-28-oic acid | -90.73 | -99.15 | 2,3,23-Trihydroxy-12-oleanen-28-oic acid, (2β,3β)-form | -96.26 | -99.93 |
| 1,3,22-Trihydroxy-12-oleanen-28-oic acid, (1α,3β,22β)-form | -66.71 | -11.98 | 2,3,23-Trihydroxy-12-oleanen-28-oic acid | -84.25 | -103.97 |
| 1,3,22-Trihydroxy-12-oleanen-28-oic acid | -90.71 | -52.69 | 2,3,23-Trihydroxy-12-oleanen-28-oic acid, (2α,3α)-form | -74.41 | -103.99 |
| 1,3,23-Trihydroxy-12-oleanen-28-oic acid, (1α,3β)-form, 1-Ketone | -85.40 | -52.12 | 2,3,23-Trihydroxy-12-oleanen-28-oic acid, (2α,3β)-form, 2-Ac, Me ester | -68.49 | -104.35 |
| 1,3,23-Trihydroxy-12-oleanen-28-oic acid, (1α,3β)-form | -74.91 | -103.98 | 2,3,23-Trihydroxy-12-oleanen-28-oic acid, (2α,3β)-form, 2-Me ether, 3,23-di-Ac | -40.10 | -96.83 |
| 1,3,23-Trihydroxy-12-oleanen-28-oic acid, (1β,3β)-form | -77.63 | -104.59 | 2,3,23-Trihydroxy-12-oleanen-28-oic acid, (2α,3β)-form, 2-Me ether | -86.71 | -93.36 |
| 1,3,23-Trihydroxy-12-oleanen-28-oic acid | -78.66 | -104.48 | 2,3,23-Trihydroxy-12-oleanen-28-oic acid, (2α,3β)-form, 23-O-(3,4,5-Trihydroxybenzoyl) | -124.65 | -96.16 |
| 1,3,9,22-Tetrahydroxy-12-oleanen-28-oic acid, (1α,3β,9α,22α)-form | -46.87 | -76.52 | 2,3,23-Trihydroxy-12-oleanen-28-oic acid, (2α,3β)-form, 23-O-(4-Hydroxy-E-cinnamoyl) | -108.86 | -98.51 |
| 1,3,9,22-Tetrahydroxy-12-oleanen-28-oic acid | -98.63 | -46.63 | 2,3,23-Trihydroxy-12-oleanen-28-oic acid, (2α,3β)-form, 3,23-Isopropylidene, 2-Ac, Me ester | -91.07 | -99.03 |
| 1,3-Dihydroxy-12-oleanen-28-oic acid, (1α,3β)-form, 1-Ketone, 28-alcohol | -86.06 | -87.99 | 2,3,23-Trihydroxy-12-oleanen-28-oic acid, (2α,3β)-form, 3,23-Isopropylidene | -45.13 | -49.42 |

**Supplementary Table 5 Cont’d:** Docking energies of *epi*-Oleanolic acid analogues for *L. infantum* and *T. cruzi* CYP51.

| **Compounds** | ***T. cruzi* CYP51** | ***L. infantum* CYP51** | **Compounds** | ***T. cruzi* CYP51** | ***L. infantum* CYP51** |
| --- | --- | --- | --- | --- | --- |
| 1,3-Dihydroxy-12-oleanen-28-oic acid, (1α,3β)-form, 1-Ketone | -86.14 | -93.99 | 2,3,23-Trihydroxy-12-oleanene-28,30-dioic acid, (2α,3α)-form | -75.31 | -39.67 |
| 1,3-Dihydroxy-12-oleanen-28-oic acid, (1α,3β)-form | -59.13 | -100.05 | 2,3,23-Trihydroxy-12-oleanene-28,30-dioic acid, (2β,3β)-form, 2,3,23-Tri-Ac, 28-Me ester | -99.24 | -81.68 |
| 1,3-Dihydroxy-12-oleanen-28-oic acid, 1-Ketone, 3-Ac | -57.66 | -102.37 | 2,3,23-Trihydroxy-12-oleanene-28,30-dioic acid, (2β,3β)-form, 23-Aldehyde, 30-Me ester | -74.40 | -93.10 |
| 1,3-Dihydroxy-12-oleanen-28-oic acid, 1-Ketone, Me ester | -49.14 | -97.00 | 2,3,23-Trihydroxy-12-oleanene-28,30-dioic acid, (2β,3β)-form, 3-Ac, 30-Me ester | -122.98 | -114.25 |
| 1,3-Dihydroxy-12-oleanen-28-oic acid, 1β-form | -83.39 | -82.33 | 2,3,23-Trihydroxy-12-oleanene-28,30-dioic acid, (2β,3β)-form, 30-Me ester | -99.38 | -78.13 |
| 1,3-Dihydroxy-12-oleanen-28-oic acid | -91.18 | -99.11 | 2,3,23-Trihydroxy-12-oleanene-28,30-dioic acid, (2β,3β)-form | -96.95 | -103.37 |
| 1,3-Dihydroxy-12-oleanen-29-oic acid, (1α,3β)-form | -85.47 | -37.48 | 2,3,23-Trihydroxy-12-oleanene-28,30-dioic acid | -98.28 | -101.87 |
| 1,3-Dihydroxy-12-oleanen-29-oic acid | -86.28 | -37.25 | 2,3,24-Trihydroxy-12-oleanene-28,30-dioic acid, (2α,3α)-form | -58.05 | -62.82 |
| 1,3-Dihydroxy-12-oleanen-30-oic acid, (1α,3β)-form | -73.77 | -56.91 | 2,3,24-Trihydroxy-12-oleanene-28,30-dioic acid | -59.90 | -42.22 |
| 1,3-Dihydroxy-12-oleanen-30-oic acid | -73.26 | -56.57 | 2,3,27-Trihydroxy-11-oxo-12-oleanene-23,28-dioic acid, (2β,3β)-form | -95.54 | -85.48 |
| 1-Hydroperoxy-3,23-dihydroxy-12-oleanen-28-oic acid, (3β,11α)-form | -93.83 | -84.13 | 2,3,27-Trihydroxy-11-oxo-12-oleanene-23,28-dioic acid | -92.93 | -85.02 |
| 11-Hydroperoxy-3,23-dihydroxy-12-oleanen-28-oic acid | -85.65 | -83.62 | 2,3,27-Trihydroxy-12-oleanene-23,28-dioic acid, (2β,3β)-form | -98.11 | -108.54 |
| 12-Oleanen-3-ol, (3β,18α)-form, Ac | -79.18 | -64.51 | 2,3,27-Trihydroxy-12-oleanene-23,28-dioic acid | -91.39 | -87.38 |
| 12-Oleanen-3-ol, (3β,18α)-form | -80.98 | -26.11 | 2,3,29-Trihydroxy-12-oleanen-28-oic acid, (2α,3β)-form | -101.00 | -36.61 |
| 12-Oleanen-3-ol, 3β-form, Eicosanoyl (β-Amyrenyl eicosanoate) | -153.42 | -130.96 | ,3,29-Trihydroxy-12-oleanen-28-oic acid, (2β,3β)-form | -104.53 | -38.40 |
| 12-Oleanen-3-ol, 3β-form, Heptadecanoyl | -123.68 | -124.63 | 2,3,29-Trihydroxy-12-oleanen-28-oic acid | -104.72 | -39.21 |
| 12-Oleanen-3-ol, 3β-form, Hexadecanoyl | -140.50 | -112.03 | 2,3,30-Trihydroxy-12-oleanen-28-oic acid, (2α,3β)-form | -101.77 | -97.19 |
| 12-Oleanen-3-ol, 3β-form, Hexanoyl | -115.16 | -97.97 | 2,3,30-Trihydroxy-12-oleanen-28-oic acid, (2β,3β)-form, 30-Aldehyde | -91.62 | -99.92 |

**Supplementary Table 5 Cont’d:** Docking energies of *epi*-Oleanolic acid analogues for *L. infantum* and *T. cruzi* CYP51.

| **Compounds** | ***T. cruzi* CYP51** | ***L. infantum* CYP51** | **Compounds** | ***T. cruzi* CYP51** | ***L. infantum* CYP51** |
| --- | --- | --- | --- | --- | --- |
| 12-Oleanen-3-ol, 3β-form, Me ether | -92.79 | -67.55 | 2,3,30-Trihydroxy-12-oleanen-28-oic acid, (2β,3β)-form | -101.24 | -102.61 |
| 12-Oleanen-3-ol, 3β-form, Nonyl ether | -119.72 | -130.53 | 2,3,30-Trihydroxy-12-oleanen-28-oic acid | -100.68 | -96.99 |
| 12-Oleanen-3-ol, 3β-form, O-(4-Hydroxy-3-methoxy-E-cinnamoyl) | -128.79 | -127.59 | 2,3,6,16,23-Pentahydroxy-12-oleanen-28-oic acid, (2β,3β,6β,16α)-form | -108.86 | -121.45 |
| 12-Oleanen-3-ol, 3β-form, O-(4-Hydroxy-E-cinnamoyl) | -116.66 | -118.57 | 2,3,6,16,23-Pentahydroxy-12-oleanen-28-oic acid | -108.84 | -121.30 |
| 12-Oleanen-3-ol, 3β-form, O-(4-Hydroxy-Z-cinnamoyl) | -115.01 | -115.73 | 2,3,6,16,24-Pentahydroxy-12-oleanen-28-oic acid, (2β,3β,6β,16α)-form | -103.17 | -50.21 |
| 12-Oleanen-3-ol, 3β-form, O-(5-Phenyl-2,4-pentadienoyl) | -127.78 | -126.27 | 2,3,6,16,24-Pentahydroxy-12-oleanen-28-oic acid | -102.89 | -57.95 |
| 12-Oleanen-3-ol, 3α-form, Ac | -102.93 | -63.89 | 2,3,6,16-Tetrahydroxy-12-oleanen-28-oic acid, (2β,3β,6β,16α)-form | -70.79 | -100.52 |
| 12-Oleanen-3-ol, 3β-form, O-(6-Oxo-2E,4E-decadienoyl | -127.13 | -128.38 | 2,3,6,16-Tetrahydroxy-12-oleanen-28-oic acid | -92.14 | -85.77 |
| 12-Oleanen-3-ol, 3β-form, O-(9Z,12Z,15Z-Octadecatrienoyl | -136.47 | -121.96 | 2,3,6,19,21,23-Hexahydroxy-12-oleanen-28-oic acid, (2α,3β,6β,19α,21β)-form | -97.77 | -111.78 |
| 12-Oleanen-3-ol, 3β-form, O-(9Z,12Z-Octadecadienoyl) (**β-amyrin linoleate**) | -151.49 | -120.65 | 2,3,6,19,21,23-Hexahydroxy-12-oleanen-28-oic acid | -54.43 | -84.54 |
| 12-Oleanen-3-ol, 3β-form, O-(9Z-Hexadecenoyl) (**β-amyrin palmitoleate**) | -153.43 | -139.31 | 2,3,6,19,23-Pentahydroxy-12-oleanen-28-oic acid, (2α,3β,6β,19α)-form | -88.33 | -94.98 |
| 12-Oleanen-3-ol, 3β-form, O-Phenylacetyl | -119.63 | -68.68 | 2,3,6,19,23-Pentahydroxy-12-oleanen-28-oic acid | -9.05 | -65.93 |
| 12-Oleanen-3-ol, 3β-form, O-[3-(4-Hydroxyphenyl)propanoyl] | -117.88 | -117.57 | 2,3,6,19,24-Pentahydroxy-11-oxo-12-oleanen-28-oic acid, (2α,3β,6β,19α)-form | -65.03 | -114.30 |
| 12-Oleanen-3-ol, 3β-form, Octadecanoyl (β-Amyrin stearate) | -146.24 | -128.58 | 2,3,6,19,24-Pentahydroxy-11-oxo-12-oleanen-28-oic acid | -72.54 | -114.72 |
| 12-Oleanen-3-ol, 3β-form, Octanoyl | -124.32 | -111.02 | 2,3,6,19-Tetrahydroxy-12-oleanen-28-oic acid, (2α,3β,6β,19α)-form | -42.50 | -83.43 |
| 12-Oleanen-3-ol, 3β-form, Propyl ether | -86.14 | -76.42 | 2,3,6,19-Tetrahydroxy-12-oleanen-28-oic acid | -98.47 | -84.08 |
| 2-Oleanen-3-ol, 3β-form, Tetradecanoyl | -127.97 | -122.19 | 2,3,6,23,24-Pentahydroxy-12-oleanen-28-oic acid, (2α,3β,6β)-form | -83.66 | -82.57 |
| 12-Oleanen-3-ol, 3α-form | -83.00 | -89.19 | 2,3,6,23,24-Pentahydroxy-12-oleanen-28-oic acid | -102.79 | -66.04 |
| 12-Oleanen-3-ol, 3β-form, Tridecanoyl | -115.99 | -117.90 | 2,3,6,23-Tetrahydroxy-12-oleanen-28-oic acid, (2α,3β,6β)-form, 23-Carboxylic acid | -62.37 | -110.15 |

**Supplementary Table 5 Cont’d:** Docking energies of *epi*-Oleanolic acid analogues for *L. infantum* and *T. cruzi* CYP51.

| **Compounds** | ***T. cruzi* CYP51** | ***L. infantum* CYP51** | **Compounds** | ***T. cruzi* CYP51** | ***L. infantum* CYP51** |
| --- | --- | --- | --- | --- | --- |
| 12-Oleanen-3-ol, 3β-form | -82.47 | -88.85 | ,3,6,23-Tetrahydroxy-12-oleanen-28-oic acid, (2α,3β,6β)-form, 23-O-(3,4,5-Trihydroxybenzoyl) | -118.97 | -138.16 |
| 12-Oleanen-3-ol | -82.83 | -89.07 | 2,3,6,23-Tetrahydroxy-12-oleanen-28-oic acid, (2α,3β,6β)-form | -42.54 | -105.57 |
| 12-Oleanen-3-ol, 3β-form, Ac | -102.67 | -63.95 | 2,3,6,23-Tetrahydroxy-12-oleanen-28-oic acid, (2β,3β,6α)-form | -60.04 | -105.52 |
| 12-Oleanen-3-ol, 3β-form, Benzoyl | -114.57 | -51.88 | 2,3,6,23-Tetrahydroxy-12-oleanen-28-oic acid, (2β,3β,6β)-form | -115.70 | -104.00 |
| 12-Oleanen-3-ol, 3β-form, Cinnamoyl | -124.56 | -80.63 | 2,3,6,23-Tetrahydroxy-12-oleanen-28-oic acid | -42.35 | -105.52 |
| 12-Oleanen-3-ol, 3β-form, Decanoyl | -131.90 | -123.59 | 2,3,6,24-Tetrahydroxy-12-oleanen-28-oic acid, (2β,3β,6β)-form | -99.26 | -92.90 |
| 12-Oleanen-3-ol, 3β-form, Docosanoyl (β-Amyrenyl behenate) | -146.79 | -121.55 | 2,3,6,24-Tetrahydroxy-12-oleanen-28-oic acid | -94.96 | -79.53 |
| 12-Oleanen-3-ol, 3β-form, Dodecanoyl | -135.03 | -124.62 | 2,3,6-Trihydroxy-12-oleanen-28-oic acid, (2α,3β,6β)-form | -96.06 | -32.41 |
| 2-Oleanene-1,2,3,11-tetrol, (1β,2α,3β,11α)-form, 3-Ac | -82.42 | -98.23 | 2,3,6-Trihydroxy-12-oleanen-28-oic acid | -96.16 | -32.22 |
| 12-Oleanene-1,2,3,11-tetrol, (1β,2α,3β,11α)-form | -63.86 | -98.62 | 2,3,7,23-Tetrahydroxy-12-oleanen-28-oic acid, (2α,3β,7α)-form | -93.08 | -48.78 |
| 12-Oleanene-1,2,3,11-tetrol | -92.27 | -95.26 | 2,3,7,23-Tetrahydroxy-12-oleanen-28-oic acid | -88.91 | -68.59 |
| 12-Oleanene-1,2,3,16,24-pentol, (1β,2α,3β,16β)-form | -90.66 | 32.50 | 2,3-Dihydroxy-11-oxo-12-oleanen-28-oic acid, (2α,3β)-form | -53.46 | -33.73 |
| 12-Oleanene-1,2,3,16,24-pentol | -90.89 | 21.43 | 2,3-Dihydroxy-11-oxo-12-oleanen-28-oic acid | -50.43 | -33.24 |
| 12-Oleanene-1,2,3-triol, (1α,2α,3β)-form | -97.03 | -89.48 | 2,3-Dihydroxy-12-oleanen-23-oic acid, (2β,3β)-form, Me ester | -90.36 | -55.11 |
| 12-Oleanene-1,2,3-triol | -100.91 | -104.99 | 2,3-Dihydroxy-12-oleanen-23-oic acid, (2β,3β)-form | -47.40 | -93.17 |
| 12-Oleanene-1,3,11,28-tetrol, (1β,3β,11α)-form, 11-Me ether | -70.63 | -75.47 | 2,3-Dihydroxy-12-oleanen-23-oic acid | -67.87 | -52.16 |
| 12-Oleanene-1,3,11,28-tetrol, (1β,3β,11α)-form | -89.90 | 29.92 | 2,3-Dihydroxy-12-oleanen-23-oic acid, (2α,3α)-form | -62.64 | -87.69 |
| 12-Oleanene-1,3,11,28-tetrol | -90.10 | 26.72 | 2,3-Dihydroxy-12-oleanen-28-oic acid, (2α,3β)-form, 2,3-O-Isopropylidene, Me ester | -40.55 | -81.78 |

**Supplementary Table 5 Cont’d:** Docking energies of *epi*-Oleanolic acid analogues for *L. infantum* and *T. cruzi* CYP51.

| **Compounds** | ***T. cruzi* CYP51** | ***L. infantum* CYP51** | **Compounds** | ***T. cruzi* CYP51** | ***L. infantum* CYP51** |
| --- | --- | --- | --- | --- | --- |
| 12-Oleanene-1,3,11-triol, (1β,3β,11α)-form, 1-(1-Hydroxy-1-methylethyl) ether, 3-Ac | 23.64 | -94.00 | 2,3-Dihydroxy-12-oleanen-28-oic acid, (2α,3β)-form, 3-O-(4-Hydroxy-E-cinnamoyl) | -85.83 | -119.80 |
| 12-Oleanene-1,3,11-triol, (1β,3β,11α)-form, 11-Et ether, 3-Ac | 78.65 | -93.61 | 2,3-Dihydroxy-12-oleanen-28-oic acid, (2α,3β)-form, 3-O-(4-Hydroxy-Z-cinnamoyl) | -73.32 | -123.56 |
| 12-Oleanene-1,3,11-triol, (1β,3β,11α)-form, 11-Me ether, 3-Ac | -20.33 | -49.41 | 2,3-Dihydroxy-12-oleanen-28-oic acid, (2α,3β)-form, Di-Ac, Me ester | -18.94 | -104.78 |
| 12-Oleanene-1,3,11-triol, (1β,3β,11α)-form | -90.61 | -8.85 | 2,3-Dihydroxy-12-oleanen-28-oic acid, (2α,3β)-form, Me ester | -93.63 | -93.99 |
| 12-Oleanene-1,3,11-triol, (1β,3β,11β)-form, 3-Hexadecanoyl | -44.81 | -129.06 | 2,3-Dihydroxy-12-oleanen-28-oic acid, (2α,3β)-form | -97.83 | -73.23 |
| 12-Oleanene-1,3,11-triol, (1β,3β,11β)-form | -90.64 | -2.27 | 2,3-Dihydroxy-12-oleanen-28-oic acid | -62.89 | -89.56 |
| 12-Oleanene-1,3,11-triol | -90.65 | -2.16 | 2,3-Dihydroxy-12-oleanen-28-oic acid, (2α,3β)-form, 2-Ac | -52.08 | -76.35 |
| 12-Oleanene-1,3,23,29-tetrol, (1α,3β)-form, 29-Carboxylic acid | -64.12 | -91.94 | 2,3-Dihydroxy-12-oleanen-28-oic acid, (2α,3β)-form, 2-Me ether, 3-Ac, Me ester | -61.56 | -92.34 |
| 12-Oleanene-1,3,23,29-tetrol, (1α,3β)-form | -60.52 | -48.73 | 2,3-Dihydroxy-12-oleanen-28-oic acid, (2α,3β)-form, 2-O-(2-Hydroxyethyl) ether | -92.02 | -92.80 |
| 12-Oleanene-1,3,23,29-tetrol | -56.32 | -86.18 | 2,3-Dihydroxy-12-oleanen-28-oic acid, (2α,3β)-form, 2-O-(3,4-Dihydroxy-E-cinnamoyl) (**2-*O*-caffeoyl maslinic acid**) | -113.99 | -151.30 |
| 12-Oleanene-1,3,23-triol, (1β,3β)-form | -90.29 | -88.54 | 2,3-Dihydroxy-12-oleanen-28-oic acid, (2α,3β)-form, 2-O-(4-Hydroxy-E-cinnamoyl) | -122.83 | -56.81 |
| 12-Oleanene-1,3,23-triol | -90.92 | -88.56 | 2,3-Dihydroxy-12-oleanen-28-oic acid, (2α,3β)-form, 3-(4-Hydroxy-3-methoxy-E-cinnamoyl) | -129.11 | -139.14 |
| 12-Oleanene-1,3-diol, (1α,3β)-form, 1-Ketone | -81.11 | -72.69 | 2,3-Dihydroxy-12-oleanen-28-oic acid, (2α,3β)-form, 3-Ac | -64.30 | -100.51 |
| 12-Oleanene-1,3-diol, (1α,3β)-form | -73.08 | -94.39 | 2,3-Dihydroxy-12-oleanen-28-oic acid, (2α,3β)-form, 3-O-(3,4-Dihydroxy-E-cinnamoyl) | 65.45 | -59.00 |
| 12-Oleanene-1,3-diol, 3α-form, 1-Ketone | -95.49 | -85.61 | 2,3-Dihydroxy-12-oleanene-23,28,30-trioic acid, (2β,3β)-form, 23,28-Di-Me ester | -104.79 | -94.27 |
| 12-Oleanene-1,3-diol, 3α-form | -98.85 | -99.34 | 2,3-Dihydroxy-12-oleanene-23,28,30-trioic acid, (2β,3β)-form | -28.91 | -74.92 |
| 12-Oleanene-1,3-diol | -84.06 | 6.73 | 2,3-Dihydroxy-12-oleanene-23,28,30-trioic acid | -28.01 | -77.47 |
| 12-Oleanene-2,3,11-triol, (2α,3β,11α)-form, 3-Ac | -108.87 | -96.64 | 2,3-Dihydroxy-12-oleanene-23,28-dioic acid, (2α,3β)-form, 2-Ketone | -86.29 | -83.59 |

**Supplementary Table 5 Cont’d:** Docking energies of *epi*-Oleanolic acid analogues for *L. infantum* and *T. cruzi* CYP51.

| **Compounds** | ***T. cruzi* CYP51** | ***L. infantum* CYP51** | **Compounds** | ***T. cruzi* CYP51** | ***L. infantum* CYP51** |
| --- | --- | --- | --- | --- | --- |
| 12-Oleanene-2,3,11-triol, (2α,3β,11α)-form | -100.70 | -88.11 | 2,3-Dihydroxy-12-oleanene-23,28-dioic acid, (2α,3β)-form, Di-Me ester | -109.42 | -116.42 |
| 12-Oleanene-2,3,11-triol | -100.91 | -88.08 | 2,3-Dihydroxy-12-oleanene-23,28-dioic acid, (2α,3β)-form | -88.70 | -69.40 |
| 12-Oleanene-2,3,15,21-tetrol, (2β,3β,15α,21β)-form | -95.36 | -97.56 | 2,3-Dihydroxy-12-oleanene-23,28-dioic acid, (2β,3β)-form | -88.76 | -66.57 |
| 12-Oleanene-2,3,15,21-tetrol | -95.47 | -97.37 | ,3-Dihydroxy-12-oleanene-23,28-dioic acid | -89.42 | -68.36 |
| 12-Oleanene-2,3,16,21,22,28-hexol, (2α,3β,16β,21β,22α)-form | -97.31 | -73.96 | 2,3-Dihydroxy-12-oleanene-24,28-dioic acid, (2α,3β)-form | -83.87 | -86.89 |
| 12-Oleanene-2,3,16,21,22,28-hexol | -97.29 | 19.51 | 2,3-Dihydroxy-12-oleanene-24,28-dioic acid | -83.76 | -93.37 |
| 12-Oleanene-2,3,16,23,28-pentol, (2β,3β,16α)-form | -70.71 | -93.84 | 2,3-Dihydroxy-12-oleanene-28,30-dioic acid, (2β,3β)-form, 2-Ac, 28-Me ester | -78.57 | -95.54 |
| 12-Oleanene-2,3,16,23,28-pentol, (2β,3β,16β)-form | -71.30 | -91.29 | 2,3-Dihydroxy-12-oleanene-28,30-dioic acid, (2β,3β)-form, 28-Me ester | -88.17 | -101.85 |
| 12-Oleanene-2,3,16,23,28-pentol, 23-Carboxylic acid | -94.27 | -49.54 | 2,3-Dihydroxy-12-oleanene-28,30-dioic acid, (2β,3β)-form, 3-Ac, 28-Me ester | -88.59 | -80.24 |
| 12-Oleanene-2,3,16,23,28-pentol, 28-Carboxylic acid, 23-aldehyde | -38.59 | -95.21 | 2,3-Dihydroxy-12-oleanene-28,30-dioic acid, (2β,3β)-form, 30-Me ester | -83.63 | -97.51 |
| 12-Oleanene-2,3,16,23,28-pentol | -31.63 | -90.92 | 2,3-Dihydroxy-12-oleanene-28,30-dioic acid, (2β,3β)-form | -85.07 | -99.92 |
| 12-Oleanene-2,3,19,23,28-pentol, (2α,3β,19α)-form | -84.09 | 4.29 | 2,3-Dihydroxy-12-oleanene-28,30-dioic acid | -84.74 | -96.51 |
| 12-Oleanene-2,3,19,23,28-pentol | -78.39 | -75.07 | 2,3-Dihydroxy-23-oxo-12-oleanen-28-oic acid, (2α,3β)-form | -75.59 | -108.67 |
| 12-Oleanene-2,3,19-triol, (2α,3α,19α)-form | -68.03 | -29.21 | 2,3-Dihydroxy-23-oxo-12-oleanen-28-oic acid, (2β,3β)-form | -62.42 | -74.73 |
| 12-Oleanene-2,3,19-triol | -42.16 | -7.87 | 2,3-Dihydroxy-23-oxo-12-oleanen-28-oic acid | -86.98 | -108.50 |
| 12-Oleanene-2,3,21,23,28-pentol, (2α,3β,21β)-form | -100.07 | -73.06 | 1,22-Epoxy-3-hydroxy-12-oleanen-28-oic acid, (3β,21β,22β)-form, Me ester | -78.92 | -84.37 |
| 12-Oleanene-2,3,21,23,28-pentol, 28-Carboxylic acid, 28→21 lactone | -104.16 | -93.34 | 21,22-Epoxy-3-hydroxy-12-oleanen-28-oic acid, (3β,21β,22β)-form | -75.55 | -52.08 |
| 2-Oleanene-2,3,21,23,28-pentol, 28-Carboxylic acid | -90.00 | -80.98 | 21,22-Epoxy-3-hydroxy-12-oleanen-28-oic acid | -74.19 | -56.03 |

**Supplementary Table 5 Cont’d:** Docking energies of *epi*-Oleanolic acid analogues for *L. infantum* and *T. cruzi* CYP51.

| **Compounds** | ***T. cruzi* CYP51** | ***L. infantum* CYP51** | **Compounds** | ***T. cruzi* CYP51** | ***L. infantum* CYP51** |
| --- | --- | --- | --- | --- | --- |
| 12-Oleanene-2,3,21,23,28-pentol | -105.72 | -71.49 | 22,30-Epoxy-12-oleanene-3,24,30-triol, (3β,22β,30R)-form, 30-Me ether | -86.94 | -37.53 |
| 12-Oleanene-2,3,21,28-tetrol, (2α,3α,21β)-form | -87.13 | -82.00 | 22,30-Epoxy-12-oleanene-3,24,30-triol, (3β,22β,30R)-form | -85.79 | -97.53 |
| 12-Oleanene-2,3,21,28-tetrol, (2α,3β,21β)-form, 28-Carboxylic acid, 28→21-lactone | -84.06 | -99.26 | 22,30-Epoxy-12-oleanene-3,24,30-triol | -85.44 | -97.50 |
| 12-Oleanene-2,3,21,28-tetrol, (2α,3β,21β)-form, 28-Carboxylic acid | -34.75 | -90.57 | 3,11,24,30-Tetrahydroxy-12-oleanen-28-oic acid, (3β,11β)-form | -107.26 | -67.08 |
| 12-Oleanene-2,3,21,28-tetrol, (2α,3β,21β)-form | -87.28 | -82.13 | 3,11,24,30-Tetrahydroxy-12-oleanen-28-oic acid | -102.64 | -44.75 |
| 12-Oleanene-2,3,21,28-tetrol, (2β,3β,21β)-form | -106.56 | -98.07 | 3,11-Dihydroxy-12-oleanen-28-oic acid, (3α,11α)-form, 11-Me ether | -96.46 | -21.21 |
| 12-Oleanene-2,3,21,28-tetrol | -100.78 | -76.52 | 3,11-Dihydroxy-12-oleanen-28-oic acid, (3α,11α)-form | -98.91 | -94.93 |
| 12-Oleanene-2,3,22-triol, (2β,3β,22α)-form | -105.95 | -15.39 | 3,15,16,21,22,28-Hexahydroxy-12-oleanen-23-oic acid, (3β,15α,16α,21β,22α)-form | -74.83 | -48.47 |
| 12-Oleanene-2,3,22-triol | -106.68 | -15.51 | 3,15,16,21,22,28-Hexahydroxy-12-oleanen-23-oic acid | -80.90 | -49.68 |
| 12-Oleanene-2,3,23,28-tetrol, (2α,3β)-form | -92.56 | -95.23 | 3,15,16-Trihydroxy-12-oleanen-28-oic acid, (3β,15α,16α)-form, Me ester | -90.31 | -93.24 |
| 12-Oleanene-2,3,23,28-tetrol, (2β,3β)-form | -92.39 | -95.24 | 3,15,16-Trihydroxy-12-oleanen-28-oic acid, (3β,15α,16α)-form | -78.67 | -88.26 |
| 12-Oleanene-2,3,23,28-tetrol | -92.46 | -95.33 | ,15,16-Trihydroxy-12-oleanen-28-oic acid, (3β,15β,16α)-form, 28→15 Lactone | -88.95 | -82.85 |
| 12-Oleanene-2,3,23,30-tetrol, (2α,3β)-form | -87.83 | -85.81 | 3,15,16-Trihydroxy-12-oleanen-28-oic acid, (3β,15β,16α)-form | -90.70 | -34.52 |
| 12-Oleanene-2,3,23,30-tetrol | -87.47 | -85.33 | 3,15,16-Trihydroxy-12-oleanen-28-oic acid | -100.73 | -82.73 |
| 12-Oleanene-2,3,23-triol, (2α,3β)-form | -97.54 | -83.91 | 3,15,19,21,22-Pentahydroxy-12-oleanen-28-oic acid, (3β,15α,19α,21β,22α)-form | -78.51 | -80.94 |
| 12-Oleanene-2,3,23-triol | -59.76 | -84.33 | 3,15,19,21,22-Pentahydroxy-12-oleanen-28-oic acid | -98.98 | -66.10 |
| 12-Oleanene-2,3,24,28-tetrol, (2α,3α)-form, 28-Carboxylic acid | -90.85 | -100.06 | 3,15,21,22,30-Pentahydroxy-12-oleanen-28-oic acid, (3β,15β,21β,22α)-form, 28→15 Lactone | -89.19 | -113.24 |
| 12-Oleanene-2,3,24,28-tetrol, (2α,3α)-form | -79.76 | -100.31 | 3,15,21,22,30-Pentahydroxy-12-oleanen-28-oic acid, (3β,15β,21β,22α)-form | -90.08 | -86.22 |

**Supplementary Table 5 Cont’d:** Docking energies of *epi*-Oleanolic acid analogues for *L. infantum* and *T. cruzi* CYP51.

| **Compounds** | ***T. cruzi* CYP51** | ***L. infantum* CYP51** | **Compounds** | ***T. cruzi* CYP51** | ***L. infantum* CYP51** |
| --- | --- | --- | --- | --- | --- |
| 12-Oleanene-2,3,24,28-tetrol, (2α,3β)-form, 28-Carboxylic acid | -91.95 | -75.65 | 3,15,21,22,30-Pentahydroxy-12-oleanen-28-oic acid | -94.26 | -83.08 |
| 12-Oleanene-2,3,24,28-tetrol, (2α,3β)-form | -92.24 | -43.59 | 3,15,21,23-Tetrahydroxy-12-oleanen-28-oic acid, (3β,15α,21β)-form | -90.43 | -35.26 |
| 12-Oleanene-2,3,24,28-tetrol, (2β,3β)-form, 28-Carboxylic acid | -92.25 | -86.48 | 3,15,21,23-Tetrahydroxy-12-oleanen-28-oic acid. | -92.90 | -90.61 |
| 12-Oleanene-2,3,24,28-tetrol, (2β,3β)-form | -76.35 | -93.48 | 3,15,21,30-Tetrahydroxy-12-oleanen-28-oic acid, (3β,15β,21β)-form, 28→15 Lactone, 21-Ac | -85.06 | -86.61 |
| 12-Oleanene-2,3,24,28-tetrol | -92.99 | -97.20 | 3,15,21,30-Tetrahydroxy-12-oleanen-28-oic acid, (3β,15β,21β)-form, 28→15 Lactone | -77.99 | -97.90 |
| 12-Oleanene-2,3,28-triol, (2α,3β)-form, (2β,3β)-form | -104.77 | -99.91 | 3,15,21,30-Tetrahydroxy-12-oleanen-28-oic acid, (3β,15β,21β)-form | -92.24 | -87.70 |
| 12-Oleanene-2,3,28-triol, (2α,3β)-form, 3-Ac | -93.14 | -44.02 | 3,15,21,30-Tetrahydroxy-12-oleanen-28-oic acid | -91.94 | -87.65 |
| 12-Oleanene-2,3,28-triol, (2α,3β)-form | -105.67 | -99.53 | 3,15,21-Trihydroxy-12-oleanen-28-oic acid, (3β,15α,21β)-form | -90.36 | -102.35 |
| 12-Oleanene-2,3,28-triol | -105.13 | -100.63 | 3,15,21-Trihydroxy-12-oleanen-28-oic acid | -90.80 | -102.06 |
| 12-Oleanene-2,3-diol, (2α,3α)-form | -93.97 | -62.74 | 3,15,22-Trihydroxy-12-oleanen-28-oic acid, (3β,15β,22α)-form, 28→15 Lactone | -75.39 | -39.37 |
| 12-Oleanene-2,3-diol, (2α,3β)-form | -66.28 | -45.32 | 3,15,22-Trihydroxy-12-oleanen-28-oic acid, (3β,15β,22α)-form | -72.79 | -96.05 |
| 12-Oleanene-2,3-diol | -101.28 | -64.01 | 3,15,22-Trihydroxy-12-oleanen-28-oic acid | -72.91 | -95.97 |
| 12-Oleanene-3,11,16,21,23,28-hexol, (3β,11α,16β,21α)-form | -85.90 | -85.39 | 3,15,23-Trihydroxy-12-oleanen-16-one, (3β,15α)-form | -88.90 | -95.51 |
| 12-Oleanene-3,11,16,21,23,28-hexol | -94.36 | -85.38 | 3,15,23-Trihydroxy-12-oleanen-16-one | -63.41 | -97.51 |
| 12-Oleanene-3,11,16,21,28-pentol, (3β,11α,16α,21α)-form | -109.63 | -95.47 | ,15,24-Trihydroxy-12-oleanene-11,21-dione, (3β,15α)-form | -93.00 | -67.73 |
| 12-Oleanene-3,11,16,21,28-pentol | -109.74 | -95.62 | 3,15,24-Trihydroxy-12-oleanene-11,21-dione | -92.97 | -66.88 |
| 12-Oleanene-3,11,16,23,28,29-hexol, (3β,11α,16β)-form | -87.64 | -37.15 | 3,15-Dihydroxy-12-oleanen-16-one, (3β,15α)-form | -55.23 | -98.43 |
| 12-Oleanene-3,11,16,23,28,29-hexol | -87.49 | -33.32 | 3,15-Dihydroxy-12-oleanen-16-one | -67.94 | -84.84 |
| 12-Oleanene-3,11,16,23,28-pentol, (3β,11α,16α)-form | -91.91 | -91.91 | 3,15-Dihydroxy-12-oleanen-28-oic acid, (3β,15β)-form, 28→15 Lactone | -76.45 | -76.41 |

**Supplementary Table 5 Cont’d:** Docking energies of *epi*-Oleanolic acid analogues for *L. infantum* and *T. cruzi* CYP51.

| **Compounds** | ***T. cruzi* CYP51** | ***L. infantum* CYP51** | **Compounds** | ***T. cruzi* CYP51** | ***L. infantum* CYP51** |
| --- | --- | --- | --- | --- | --- |
| 12-Oleanene-3,11,16,23,28-pentol, (3β,11α,16β)-form, 11-Ketone | -53.09 | -87.33 | 3,15-Dihydroxy-12-oleanen-28-oic acid, (3β,15β)-form | -80.28 | -74.49 |
| 12-Oleanene-3,11,16,23,28-pentol, (3β,11α,16β)-form | -46.96 | -94.22 | 3,15-Dihydroxy-12-oleanen-28-oic acid | -90.97 | -9.80 |
| 12-Oleanene-3,11,16,23,28-pentol, (3β,11β,16β)-form | -71.40 | -88.26 | 3,16,21,30-Tetrahydroxy-12-oleanen-28-oic acid, (3β,16α,21β)-form, 28→21-Lactone | -0.50 | -38.89 |
| 12-Oleanene-3,11,16,23,28-pentol | -73.83 | -88.67 | 3,16,21,30-Tetrahydroxy-12-oleanen-28-oic acid, (3β,16α,21β)-form, 30-Carboxylic acid, 28→21-lactone | -61.27 | -105.73 |
| 12-Oleanene-3,11,16,28-tetrol, (3β,11α,16α)-form, 16-Ketone, 3-Ac | -97.64 | -90.53 | 3,16,21,30-Tetrahydroxy-12-oleanen-28-oic acid, (3β,16α,21β)-form | -102.31 | -68.21 |
| 12-Oleanene-3,11,16,28-tetrol, (3β,11α,16α)-form | -80.42 | -50.77 | 3,16,21,30-Tetrahydroxy-12-oleanen-28-oic acid | -102.35 | -66.28 |
| 12-Oleanene-3,11,16,28-tetrol, (3β,11α,16β)-form | -97.13 | -19.53 | 3,16,21-Trihydroxy-12-oleanen-28-oic acid, (3β,16α,21β)-form, 21-Cinnamoyl(E-) | -71.39 | -39.68 |
| 12-Oleanene-3,11,16,28-tetrol | -99.75 | -100.86 | 3,16,21-Trihydroxy-12-oleanen-28-oic acid, (3β,16α,21β)-form, 28→21-Lactone, 3-Ac | -60.87 | -86.91 |
| 12-Oleanene-3,11,16-triol, (3β,11α,16β)-form, 11-Ketone, 3-Ac | -87.35 | -91.24 | 3,16,21-Trihydroxy-12-oleanen-28-oic acid, (3β,16α,21β)-form, 28→21-Lactone | -84.88 | -78.16 |
| 12-Oleanene-3,11,16-triol, (3β,11α,16β)-form, 3-Hexadecanoyl | -135.05 | -134.44 | 3,16,21-Trihydroxy-12-oleanen-28-oic acid, (3β,16α,21β)-form | -74.50 | -49.94 |
| 12-Oleanene-3,11,16-triol, (3β,11α,16β)-form | -100.08 | -49.31 | 3,16,21-Trihydroxy-12-oleanen-28-oic acid, (3β,16β,21β)-form, 28→21-Lactone | -87.97 | -85.10 |
| 12-Oleanene-3,11,16-triol | -80.04 | -24.50 | 3,16,21-Trihydroxy-12-oleanen-28-oic acid, (3β,16β,21β)-form | -14.45 | -33.74 |
| 2-Oleanene-3,11,21-triol, (3α,11α,21β)-form, 11-Me ether | -81.15 | -97.84 | 3,16,21-Trihydroxy-12-oleanen-28-oic acid | -74.04 | -49.58 |
| 12-Oleanene-3,11,21-triol, (3α,11α,21β)-form | -82.72 | -72.07 | 3,16,22,28-Tetrahydroxy-12-oleanen-30-oic acid, (3β,16β,22β)-form | -55.86 | -79.44 |
| 12-Oleanene-3,11,21-triol, (3β,11α,21β)-form | -83.42 | -33.28 | 3,16,22,28-Tetrahydroxy-12-oleanen-30-oic acid | -55.80 | -79.50 |
| 12-Oleanene-3,11,21-triol | -92.70 | -18.52 | 3,16,22-Trihydroxy-23-oxo-12-oleanen-28-oic acid, (3β,16α,22β)-form | -79.39 | -25.44 |
| 12-Oleanene-3,11,22,24-tetrol, (3β,11ξ,22α)-form | -56.48 | -63.71 | 3,16,22-Trihydroxy-23-oxo-12-oleanen-28-oic acid | -79.60 | -25.28 |

**Supplementary Table 5 Cont’d:** Docking energies of *epi*-Oleanolic acid analogues for *L. infantum* and *T. cruzi* CYP51.

| **Compounds** | ***T. cruzi* CYP51** | ***L. infantum* CYP51** | **Compounds** | ***T. cruzi* CYP51** | ***L. infantum* CYP51** |
| --- | --- | --- | --- | --- | --- |
| 12-Oleanene-3,11,22,24-tetrol, (3β,22β)-form | -75.34 | -78.31 | 3,16,23-Trihydroxy-12-oleanen-28-oic acid, (3β,16α)-form | 55.45 | 6.42 |
| 12-Oleanene-3,11,22,24-tetrol | -70.02 | -76.96 | 3,16,23-Trihydroxy-12-oleanen-28-oic acid, (3β,16β)-form, Tri-Ac | -76.22 | -65.46 |
| 12-Oleanene-3,11,23,28-tetrol, (3β,11α)-form | -96.92 | -17.81 | 3,16,23-Trihydroxy-12-oleanen-28-oic acid, (3β,16β)-form | -93.99 | -103.01 |
| 12-Oleanene-3,11,23,28-tetrol, (3β,11ξ)-form | -94.56 | -98.48 | 3,16,23-Trihydroxy-12-oleanen-28-oic acid | 51.18 | 5.94 |
| 12-Oleanene-3,11,23,28-tetrol | -71.11 | -16.79 | 3,16,24-Trihydroxy-12-oleanen-28-oic acid, (3β,16α)-form | -92.73 | -85.61 |
| 12-Oleanene-3,11,28-triol, (3β,11α)-form | -63.76 | -81.17 | 3,16,24-Trihydroxy-12-oleanen-28-oic acid | -93.31 | -64.39 |
| 12-Oleanene-3,11,28-triol | -68.76 | -36.74 | 3,16,28-Trihydroxy-12-oleanen-21-one, (3β,16α)-form | -68.01 | -85.41 |
| 12-Oleanene-3,11-diol, (3α,11α)-form, 11-Me ether | -53.22 | -85.13 | 3,16,28-Trihydroxy-12-oleanen-21-one | -86.95 | -85.34 |
| 12-Oleanene-3,11-diol, (3α,11α)-form | -87.16 | -39.74 | 3,16,28-Trihydroxy-12-oleanen-30-oic acid, (3β,16α)-form | -81.44 | -64.59 |
| 12-Oleanene-3,11-diol, (3β,11α)-form, 11-Me ether, 3-Ac | -37.19 | -56.45 | 3,16,28-Trihydroxy-12-oleanen-30-oic acid | -81.11 | -60.64 |
| 12-Oleanene-3,11-diol, (3β,11α)-form, 11-Me ether | -69.91 | 12.12 | 3,16-Dihydroxy-12-oleanen-15-one, (3β,16β)-form | -71.85 | -96.39 |
| 12-Oleanene-3,11-diol, (3β,11α)-form, 11-O-(αR,βS-Epoxy-4-hydroxy-3-methoxycinnamoyl), 3-Ac | -41.13 | -72.72 | 3,16-Dihydroxy-12-oleanen-15-one | -72.18 | -96.62 |
| 12-Oleanene-3,11-diol, (3β,11α)-form, 3-Ac | -65.26 | -96.34 | 3,16-Dihydroxy-12-oleanen-28-oic acid, (3β,16α)-form, 3-O-Sulfate | -37.69 | -96.00 |
| 12-Oleanene-3,11-diol, (3β,11α)-form | -82.13 | -58.77 | 3,16-Dihydroxy-12-oleanen-28-oic acid, (3β,16α)-form | -78.26 | -96.86 |
| 12-Oleanene-3,11-diol, (3β,11β)-form, 3-Hexadecanoyl | -122.31 | -120.26 | 3,16-Dihydroxy-12-oleanen-28-oic acid | -76.96 | -98.27 |
| 12-Oleanene-3,11-diol, (3β,11β)-form | -80.66 | -40.79 | 3,16-Dihydroxy-12-oleanene-23,28-dioic acid, (3β,16α)-form | -85.11 | -69.42 |
| 12-Oleanene-3,11-diol, (3β,11ξ)-form | -45.66 | -40.30 | 3,16-Dihydroxy-12-oleanene-23,28-dioic acid | -85.35 | -62.03 |
| 12-Oleanene-3,11-diol | -82.02 | -62.64 | 3,16-Dihydroxy-12-oleanene-28,30-dioic acid, (3β,16α)-form | -87.59 | -89.58 |

**Supplementary Table 5 Cont’d:** Docking energies of *epi*-Oleanolic acid analogues for *L. infantum* and *T. cruzi* CYP51.

| **Compounds** | ***T. cruzi* CYP51** | ***L. infantum* CYP51** | **Compounds** | ***T. cruzi* CYP51** | ***L. infantum* CYP51** |
| --- | --- | --- | --- | --- | --- |
| 12-Oleanene-3,11-diol, (3β,11α)-form, 11-Et ether, 3-Ac | -74.97 | -96.10 | 3,16-Dihydroxy-12-oleanene-28,30-dioic acid | -90.00 | -90.18 |
| 12-Oleanene-3,11-diol, (3β,11α)-form, 11-Et ether | -77.67 | -78.64 | 3,16-Dihydroxy-23-oxo-12-oleanen-28-oic acid, (3β,16α)-form, Me ester | -91.00 | -44.59 |
| 12-Oleanene-3,11-diol, (3β,11α)-form, 11-Hydroperoxide, 3-Ac | -88.97 | -65.90 | 3,16-Dihydroxy-23-oxo-12-oleanen-28-oic acid, (3β,16α)-form | -84.54 | -33.65 |
| 12-Oleanene-3,11-diol, (3β,11α)-form, 11-Ketone, 3-Ac | -37.30 | 0.23 | 3,16-Dihydroxy-23-oxo-12-oleanen-28-oic acid | -82.58 | -33.21 |
| 12-Oleanene-3,11-diol, (3β,11α)-form, 11-Ketone, 3-O-(3,4-dihydroxy-E-cinnamoyl) | -118.64 | -122.52 | 3,18,21-Trihydroxy-11-oxo-12-oleanen-29-oic acid, (3β,18α,21α)-form, 29 →18 Lactone | -77.44 | -100.12 |
| 12-Oleanene-3,11-diol, (3β,11α)-form, 11-Ketone, 3-O-(3,5-dihydroxy-E-cinnamoyl) | -66.92 | -83.87 | 3,18,21-Trihydroxy-11-oxo-12-oleanen-29-oic acid, (3β,18α,21α)-form | -106.71 | -70.74 |
| 12-Oleanene-3,11-diol, (3β,11α)-form, 11-Ketone, 3-hexadecanoyl | -112.99 | -91.04 | 3,18,21-Trihydroxy-11-oxo-12-oleanen-29-oic acid | -106.81 | -73.31 |
| 12-Oleanene-3,11-diol, (3β,11α)-form, 11-Ketone | -86.62 | -98.89 | 3,18-Dihydroxy-11-oxo-12-oleanen-29-oic acid, (3β,18α)-form, 29→18 lactone | -85.63 | -2.96 |
| 12-Oleanene-3,15,16,21,22,24,28-heptol, (3β,15α,16α,21β,22α)-form, 21,22-Diangeloyl | -85.18 | -90.63 | 3,18-Dihydroxy-11-oxo-12-oleanen-29-oic acid, (3β,18α)-form | -94.60 | -32.05 |
| 12-Oleanene-3,15,16,21,22,24,28-heptol, (3β,15α,16α,21β,22α)-form, 21-Angeloyl | -49.10 | -109.01 | 3,18-Dihydroxy-11-oxo-12-oleanen-29-oic acid | -95.00 | -31.83 |
| 12-Oleanene-3,15,16,21,22,24,28-heptol, (3β,15α,16α,21β,22α)-form | -58.69 | -96.36 | 3,18-Dihydroxy-11-oxo-12-oleanen-30-oic acid, (3β,18α)-form | -76.65 | -62.29 |
| 2-Oleanene-3,15,16,21,22,24,28-heptol | -36.90 | -96.06 | 3,18-Dihydroxy-11-oxo-12-oleanen-30-oic acid, (3β,18β)-form, 30→18 Lactone, 3-Ac | -75.35 | -73.11 |
| 12-Oleanene-3,15,16,21,22,28-hexol, (3β,15α,16α,21β,22α)-form, 21,22-Diangeloyl | -45.32 | -42.68 | 3,18-Dihydroxy-11-oxo-12-oleanen-30-oic acid, (3β,18β)-form | -86.14 | -97.77 |
| 12-Oleanene-3,15,16,21,22,28-hexol, (3β,15α,16α,21β,22α)-form, 21-Angeloyl, 22-Ac | -109.70 | -108.71 | 3,18-Dihydroxy-11-oxo-12-oleanen-30-oic acid | -86.97 | -97.69 |
| 12-Oleanene-3,15,16,21,22,28-hexol, (3β,15α,16α,21β,22α)-form, 22-Tigloyl | -68.23 | 24.94 | 3,19,23,24-Tetrahydroxy-12-oleanen-28-oic acid, (3β,19α)-form, 24-Carboxylic acid | -102.65 | -74.13 |
| 12-Oleanene-3,15,16,21,22,28-hexol, (3β,15α,16α,21β,22α)-form | -70.65 | -13.57 | 3,19,23,24-Tetrahydroxy-12-oleanen-28-oic acid, (3β,19α)-form | -87.61 | -112.64 |
| 12-Oleanene-3,15,16,21,22,28-hexol | -53.65 | -10.49 | 3,19,23,24-Tetrahydroxy-12-oleanen-28-oic acid | -101.23 | -97.23 |

**Supplementary Table 5 Cont’d:** Docking energies of *epi*-Oleanolic acid analogues for *L. infantum* and *T. cruzi* CYP51.

| **Compounds** | ***T. cruzi* CYP51** | ***L. infantum* CYP51** | **Compounds** | ***T. cruzi* CYP51** | ***L. infantum* CYP51** |
| --- | --- | --- | --- | --- | --- |
| 12-Oleanene-3,15,16,21,22,28-hexol, (3β,15α,16α,21β,22α)-form, 21-Angeloyl, 22-O-(3-methyl-2-butenoyl) | -115.91 | -114.48 | 3,19,23-Trihydroxy-12-oleanen-28-oic acid, (3β,19α)-form, 23-Aldehyde | -37.46 | -105.70 |
| 12-Oleanene-3,15,16,21,22,28-hexol, (3β,15α,16α,21β,22α)-form, 21-Angeloyl | -97.36 | -103.59 | 3,19,23-Trihydroxy-12-oleanen-28-oic acid, (3β,19α)-form, 23-Carboxylic acid | -86.21 | -77.83 |
| 12-Oleanene-3,15,16,21,22,28-hexol, (3β,15α,16α,21β,22α)-form, 21-O-(2-Methyl-2-butenoyl) | -48.75 | -104.03 | 3,19,23-Trihydroxy-12-oleanen-28-oic acid, (3β,19α)-form | -98.27 | -100.83 |
| 12-Oleanene-3,15,16,21,22,28-hexol, (3β,15α,16α,21β,22α)-form, 21-O-(3-Methyl-2-butenoyl) | -98.95 | 12.89 | 3,19,23-Trihydroxy-12-oleanen-28-oic acid | -98.61 | -100.75 |
| 12-Oleanene-3,15,16,21,22,28-hexol, (3β,15α,16α,21β,22α)-form, 21-O-(3-Methylbutanoyl), 15,22-di-Ac | -80.99 | -76.81 | ,19,24-Trihydroxy-12-oleanen-28-oic acid, (3α,19α)-form | -89.29 | -112.58 |
| 12-Oleanene-3,15,16,21,22,28-hexol, (3β,15α,16α,21β,22α)-form, 22-Angeloyl | -89.09 | -80.19 | 3,19,24-Trihydroxy-12-oleanen-28-oic acid, (3β,19α)-form | -89.65 | -112.62 |
| 12-Oleanene-3,15,16,21,22,28-hexol, (3β,15α,16α,21β,22α)-form, 22-O-(2-Methylbutanoyl), 21-Ac | -78.49 | -26.92 | 3,19,24-Trihydroxy-12-oleanen-28-oic acid | -91.41 | -112.59 |
| 12-Oleanene-3,15,16,21,22,28-hexol, (3β,15α,16α,21β,22α)-form, 22-O-(3-Methylbutanoyl) | -91.71 | -94.36 | 3,19-Dihydroxy-12-oleanen-28,21-olide, (3β,19α,21β)-form | -107.86 | -88.46 |
| 12-Oleanene-3,15,16,22,28-pentol, (3β,15α,16α,22α)-form, 22-Angeloyl | -90.05 | -50.30 | 3,19-Dihydroxy-12-oleanen-28,21-olide | -107.74 | -88.42 |
| 12-Oleanene-3,15,16,22,28-pentol, (3β,15α,16α,22α)-form, 22-O-(2-Methyl-2-butenoyl) | -106.66 | -120.06 | 3,19-Dihydroxy-12-oleanen-28-oic acid, (3α,19α)-form | -89.52 | -57.20 |
| 12-Oleanene-3,15,16,22,28-pentol, (3β,15α,16α,22α)-form, 22-O-(2-Methylbutanoyl) | -72.68 | -46.24 | 3,19-Dihydroxy-12-oleanen-28-oic acid, (3β,19α)-form, 3-Ac | -71.10 | -84.76 |
| 12-Oleanene-3,15,16,22,28-pentol, (3β,15α,16α,22α)-form, 22-O-(3-Methyl-2-butenoyl) | -101.62 | -50.04 | 3,19-Dihydroxy-12-oleanen-28-oic acid, (3β,19α)-form | -89.42 | -55.29 |
| 12-Oleanene-3,15,16,22,28-pentol, (3β,15α,16α,22α)-form, 28-Angeloyl | 107.09 | -0.26 | ,19-Dihydroxy-12-oleanen-28-oic acid, (3β,19β)-form | -89.25 | -57.26 |

**Supplementary Table 5 Cont’d:** Docking energies of *epi*-Oleanolic acid analogues for *L. infantum* and *T. cruzi* CYP51.

| **Compounds** | ***T. cruzi* CYP51** | ***L. infantum* CYP51** | **Compounds** | ***T. cruzi* CYP51** | ***L. infantum* CYP51** |
| --- | --- | --- | --- | --- | --- |
| 12-Oleanene-3,15,16,22,28-pentol, (3β,15α,16α,22α)-form | -80.95 | -104.06 | 3,19-Dihydroxy-12-oleanen-28-oic acid | -89.40 | -57.28 |
| 12-Oleanene-3,15,16,22,28-pentol | -81.35 | -104.00 | 3,19-Dihydroxy-12-oleanene-24,28-dioic acid, (3β,19α)-form | -77.24 | -95.09 |
| 12-Oleanene-3,15,22,24-tetrol, (3β,15α,22β)-form | -102.15 | -99.05 | 3,19-Dihydroxy-12-oleanene-24,28-dioic acid | -78.19 | -96.75 |
| 12-Oleanene-3,15,22,24-tetrol | -102.33 | -99.06 | 3,21,22,24-Tetrahydroxy-12-oleanen-29-oic acid, (3β,21β,22β)-form, Me ester | -45.80 | -104.42 |
| 12-Oleanene-3,15,22,28-tetrol, (3β,15ξ,22α)-form | -81.56 | -95.64 | 3,21,22,24-Tetrahydroxy-12-oleanen-29-oic acid, (3β,21β,22β)-form | -68.34 | -25.28 |
| 12-Oleanene-3,15,22,28-tetrol | -90.25 | -95.10 | 3,21,22,24-Tetrahydroxy-12-oleanen-29-oic acid | -82.05 | -97.88 |
| 2-Oleanene-3,15,24-triol, (3β,15α)-form | -62.71 | -99.87 | 3,21,22-Trihydroxy-12-oleanen-28-oic acid, (3β,21α,22α)-form | -79.62 | -60.08 |
| 12-Oleanene-3,15,24-triol | -62.42 | -102.32 | 3,21,22-Trihydroxy-12-oleanen-28-oic acid, (3β,21β,22α)-form, 21-(6-Methyl-2E,4E-octadienoyl), 22-Ac | -107.98 | -58.62 |
| 2-Oleanene-3,15,28,30-tetrol, (3β,15α)-form | -78.57 | -81.81 | 3,21,22-Trihydroxy-12-oleanen-28-oic acid, (3β,21β,22α)-form, 21-(6-Methyl-2Z,4E-octadienoyl), 22-Ac | -112.26 | -68.48 |
| 12-Oleanene-3,15,28,30-tetrol | -43.64 | -88.73 | ,21,22-Trihydroxy-12-oleanen-28-oic acid, (3β,21β,22α)-form | -94.00 | -66.27 |
| 12-Oleanene-3,15-diol, (3β,15α)-form, 3-O-(4-Hydroxy-E-cinnamoyl) | -100.82 | -118.25 | 3,21,22-Trihydroxy-12-oleanen-28-oic acid | -95.23 | -90.80 |
| 12-Oleanene-3,15-diol, (3β,15α)-form, 3-O-(4-Methoxy-E-cinnamoyl) | -85.85 | -137.40 | 3,21,23-Trihydroxy-12-oleanen-28-oic acid, (3β,21β)-form, 23-Aldehyde | -100.98 | -37.26 |
| 12-Oleanene-3,15-diol, (3β,15α)-form | -73.32 | -83.11 | 3,21,23-Trihydroxy-12-oleanen-28-oic acid, (3β,21β)-form | -72.51 | -73.70 |
| 12-Oleanene-3,15-diol | -73.68 | -83.22 | 3,21,23-Trihydroxy-12-oleanen-28-oic acid | -72.54 | -73.28 |
| 12-Oleanene-3,16,21,22,23,28-hexol, (3β,16α,21β,22α)-form, 21,22-Bis-O-(2-methylbutanoyl) | -88.64 | -93.85 | 3,21,24-Trihydroxy-12-oleanen-22-one, (3β,21β)-form | -95.52 | -67.10 |
| 12-Oleanene-3,16,21,22,23,28-hexol, (3β,16α,21β,22α)-form, 21,22-Diangeloyl | -64.56 | -108.36 | 3,21,24-Trihydroxy-12-oleanen-22-one | -95.59 | -65.10 |
| 12-Oleanene-3,16,21,22,23,28-hexol, (3β,16α,21β,22α)-form, 23-Aldehyde | -90.71 | -92.35 | 3,21,24-Trihydroxy-12-oleanen-29-oic acid, (3β,21α)-form | -77.90 | -61.54 |

**Supplementary Table 5 Cont’d:** Docking energies of *epi*-Oleanolic acid analogues for *L. infantum* and *T. cruzi* CYP51.

| **Compounds** | ***T. cruzi* CYP51** | ***L. infantum* CYP51** | **Compounds** | ***T. cruzi* CYP51** | ***L. infantum* CYP51** |
| --- | --- | --- | --- | --- | --- |
| 12-Oleanene-3,16,21,22,23,28-hexol, (3β,16α,21β,22α)-form, 3,21,22,28-Tetra-Ac | -83.21 | -110.21 | 3,21,24-Trihydroxy-12-oleanen-29-oic acid | -43.88 | -75.28 |
| 12-Oleanene-3,16,21,22,23,28-hexol, (3β,16α,21β,22α)-form | -72.02 | -30.74 | 3,21,30-Trihydroxy-12-oleanen-28-oic acid, (3β,21β)-form, 28→21 Lactone | -72.39 | -61.25 |
| 12-Oleanene-3,16,21,22,23,28-hexol, (3β,16β,21β,22α)-form | -71.91 | -45.57 | 3,21,30-Trihydroxy-12-oleanen-28-oic acid, (3β,21β)-form | -90.17 | -22.63 |
| 12-Oleanene-3,16,21,22,23,28-hexol | -70.51 | -26.71 | 3,21,30-Trihydroxy-12-oleanen-28-oic acid | -92.69 | -24.15 |
| 12-Oleanene-3,16,21,22,23,28-hexol, (3β,16α,21β,22α)-form, 21,22-Ditigloyl | -83.96 | -67.65 | 3,21-Dihydroxy-11-oxo-12-oleanen-29-oic acid, (3β,21α)-form, Me ester | -97.21 | -95.59 |
| 12-Oleanene-3,16,21,22,23,28-hexol, (3β,16α,21β,22α)-form, 21-Angeloyl | -105.28 | -86.51 | 3,21-Dihydroxy-11-oxo-12-oleanen-29-oic acid, (3β,21α)-form | -95.83 | -91.33 |
| 12-Oleanene-3,16,21,22,23,28-hexol, (3β,16α,21β,22α)-form, 21-O-(2-Methylbutanoyl) | -64.94 | -64.05 | 3,21-Dihydroxy-11-oxo-12-oleanen-29-oic acid | -96.27 | -91.43 |
| 12-Oleanene-3,16,21,22,23,28-hexol, (3β,16α,21β,22α)-form, 21-O-(3-Methylbutanoyl) | -48.56 | -77.39 | 3,21-Dihydroxy-12-oleanen-28-oic acid, (3β,21α)-form | -77.63 | -26.93 |
| 2-Oleanene-3,16,21,22,23,28-hexol, (3β,16α,21β,22α)-form, 21-Tigloyl | -110.49 | -91.83 | ,21-Dihydroxy-12-oleanen-28-oic acid, (3β,21β)-form, 21-Cinnamoyl | -111.35 | -94.94 |
| 12-Oleanene-3,16,21,22,23,28-hexol, (3β,16α,21β,22α)-form, 22-Angeloyl. | -78.14 | -52.68 | 3,21-Dihydroxy-12-oleanen-28-oic acid, (3β,21β)-form, 21-Ketone | -94.18 | -40.38 |
| 12-Oleanene-3,16,21,22,23,28-hexol, (3β,16α,21β,22α)-form, 23-Aldehyde, 21,22-di-Ac | -80.39 | -60.92 | 3,21-Dihydroxy-12-oleanen-28-oic acid, (3β,21β)-form, 21-O-(4-Methoxy-E-cinnamoyl) | -104.84 | -104.79 |
| 12-Oleanene-3,16,21,22,23,28-hexol, (3β,16α,21β,22α)-form, 23-Aldehyde, 22-angeloyl | -71.71 | -79.76 | 3,21-Dihydroxy-12-oleanen-28-oic acid, (3β,21β)-form, 28→21 Lactone | -76.83 | -57.83 |
| 12-Oleanene-3,16,21,22,24,28-hexol, (3β,16α,21β,22α)-form, 21,22-Diangeloyl | -87.84 | -65.63 | 3,21-Dihydroxy-12-oleanen-28-oic acid, (3β,21β)-form, Et ester | -98.92 | -46.08 |
| 12-Oleanene-3,16,21,22,24,28-hexol, (3β,16α,21β,22α)-form, 21-Angeloyl | -75.51 | -88.00 | 3,21-Dihydroxy-12-oleanen-28-oic acid, (3β,21β)-form | -78.61 | -29.08 |
| 12-Oleanene-3,16,21,22,24,28-hexol, (3β,16α,21β,22α)-form, 21-Tigloyl, 16-Ac | -105.02 | -10.70 | (3,21-Dihydroxy-12-oleanen-28-oic acid, 3α-form, 21-Ketone | -93.71 | -40.46 |
| 12-Oleanene-3,16,21,22,24,28-hexol, (3β,16α,21β,22α)-form, 21-Tigloyl, 22-Ac | -106.26 | -106.75 | 3,21-Dihydroxy-12-oleanen-28-oic acid, 3α-form | -82.60 | -32.50 |
| 12-Oleanene-3,16,21,22,24,28-hexol, (3β,16α,21β,22α)-form, 21-Tigloyl | -92.40 | -109.43 | 3,21-Dihydroxy-12-oleanen-28-oic acid | -77.49 | -28.29 |

**Supplementary Table 5 Cont’d:** Docking energies of *epi*-Oleanolic acid analogues for *L. infantum* and *T. cruzi* CYP51.

| **Compounds** | ***T. cruzi* CYP51** | ***L. infantum* CYP51** | **Compounds** | ***T. cruzi* CYP51** | ***L. infantum* CYP51** |
| --- | --- | --- | --- | --- | --- |
| 12-Oleanene-3,16,21,22,24,28-hexol, (3β,16α,21β,22α)-form, 24-Aldehyde, 21,22-diangeloyl | -67.62 | -62.57 | 3,21-Dihydroxy-12-oleanene-28,29-dioic acid, (3β,21β)-form, 28-Alcohol | -82.74 | -69.75 |
| 12-Oleanene-3,16,21,22,24,28-hexol, (3β,16α,21β,22α)-form, 24-Aldehyde | -83.74 | -98.50 | 3,21-Dihydroxy-12-oleanene-28,29-dioic acid, (3β,21β)-form | -102.18 | -54.29 |
| 12-Oleanene-3,16,21,22,24,28-hexol, (3β,16α,21β,22α)-form | -86.45 | -96.47 | 3,21-Dihydroxy-12-oleanene-28,29-dioic acid | -101.80 | -59.33 |
| 12-Oleanene-3,16,21,22,24,28-hexol | -86.38 | -96.44 | 3,21-Dihydroxy-7-oxo-12-oleanen-28-oic acid, (3β,21β)-form | -79.69 | -13.39 |
| 12-Oleanene-3,16,21,22,28,29-hexol, (3β,16α,21β,22α)-form | -97.29 | 3.63 | 3,21-Dihydroxy-7-oxo-12-oleanen-28-oic acid | -81.45 | 24.80 |
| 12-Oleanene-3,16,21,22,28,29-hexol | -58.41 | -93.40 | 3,22,23-Trihydroxy-12-oleanen-28-oic acid, (3β,22α)-form | -31.76 | -76.85 |
| 12-Oleanene-3,16,21,22,28-pentol, (3α,16α,21α,22α)-form | -63.69 | -84.11 | 3,22,23-Trihydroxy-12-oleanen-28-oic acid | -96.68 | -85.39 |
| 12-Oleanene-3,16,21,22,28-pentol, (3α,16α,21β,22α)-form | -103.32 | -90.30 | 3,22,24,29-Tetrahydroxy-12-oleanen-30-oic acid, (3β,22β)-form, 30→22 Lactone | -89.14 | -63.26 |
| 12-Oleanene-3,16,21,22,28-pentol, (3β,16α,21β,22α)-form, 22-Angeloyl | -71.84 | -115.98 | 3,22,24,29-Tetrahydroxy-12-oleanen-30-oic acid, (3β,22β)-form | -75.50 | -80.46 |
| 12-Oleanene-3,16,21,22,28-pentol, (3β,16α,21β,22α)-form, 22-Ketone | -75.52 | -91.42 | 3,22,24,29-Tetrahydroxy-12-oleanen-30-oic acid | -83.44 | -76.03 |
| 12-Oleanene-3,16,21,22,28-pentol, (3β,16α,21β,22α)-form, 22-O-(3-Methyl-2-butenoyl) | -74.58 | -118.03 | 3,22,24-Trihydroxy-11-oxo-12-oleanen-30-oic acid, (3β,22α)-form | -81.75 | -80.25 |
| 12-Oleanene-3,16,21,22,28-pentol, (3β,16α,21β,22α)-form, 28-Aldehyde | -102.76 | -88.22 | 3,22,24-Trihydroxy-11-oxo-12-oleanen-30-oic acid, (3β,22β)-form | -81.84 | -80.40 |
| 12-Oleanene-3,16,21,22,28-pentol, (3β,16α,21β,22α)-form | -86.79 | -81.56 | 3,22,24-Trihydroxy-11-oxo-12-oleanen-30-oic acid, 30→22 Lactone | -35.74 | -63.81 |
| 12-Oleanene-3,16,21,22,28-pentol, (3β,16β,21β,22α)-form | -101.69 | -88.79 | 3,22,24-Trihydroxy-11-oxo-12-oleanen-30-oic acid | -81.53 | -80.16 |
| 12-Oleanene-3,16,21,22,28-pentol | -34.34 | -79.36 | 3,22,24-Trihydroxy-12-oleanen-19-one, (3β, 19β, 22β)-form | -68.91 | -76.51 |
| 12-Oleanene-3,16,21,22,28-pentol, (3β,16α,21β,22α)-form, 21,22-Diangeloyl | -60.56 | -90.04 | 3,22,24-Trihydroxy-12-oleanen-19-one | -68.75 | -76.34 |

**Supplementary Table 5 Cont’d:** Docking energies of *epi*-Oleanolic acid analogues for *L. infantum* and *T. cruzi* CYP51.

| **Compounds** | ***T. cruzi* CYP51** | ***L. infantum* CYP51** | **Compounds** | ***T. cruzi* CYP51** | ***L. infantum* CYP51** |
| --- | --- | --- | --- | --- | --- |
| 12-Oleanene-3,16,21,22,28-pentol, (3β,16α,21β,22α)-form, 21,22-Dibenzoyl. | -101.13 | -116.84 | 3,22,24-Trihydroxy-12-oleanen-28-al, (3β,22β)-form | -77.71 | 33.60 |
| 12-Oleanene-3,16,21,22,28-pentol, (3β,16α,21β,22α)-form, 21-Angeloyl | -123.07 | -105.89 | 3,22,24-Trihydroxy-12-oleanen-28-al | -53.41 | 35.09 |
| 12-Oleanene-3,16,21,22,28-pentol, (3β,16α,21β,22α)-form, 21-Benzoyl | -95.92 | -66.01 | 3,22,24-Trihydroxy-12-oleanen-28-oic acid, (3β,22β)-form, 22-Angeloyl | -94.99 | -107.08 |
| 12-Oleanene-3,16,21,22,28-pentol, (3β,16α,21β,22α)-form, 21-O-(2,3-Dihydroxy-2-methylbutanoyl), 22-angeloyl | -19.90 | -100.66 | 3,22,24-Trihydroxy-12-oleanen-28-oic acid, (3β,22β)-form | -73.26 | -72.85 |
| 12-Oleanene-3,16,21,22,28-pentol, (3β,16α,21β,22α)-form, 21-O-(2,3-Epoxy-2-methylbutanoyl), 22-angeloyl | -87.81 | -97.02 | 3,22,24-Trihydroxy-12-oleanen-28-oic acid | -74.30 | -73.26 |
| 12-Oleanene-3,16,21,22,28-pentol, (3β,16α,21β,22α)-form, 21-Tigloyl | -124.33 | -39.70 | 3,22,24-Trihydroxy-12-oleanen-29-oic acid, (3β,22β)-form | -82.12 | -96.42 |
| 12-Oleanene-3,16,21,22,28-pentol, (3β,16α,21β,22α)-form, 22-Angeloyl, 16,28-di-Ac | -80.74 | -118.19 | 3,22,24-Trihydroxy-12-oleanen-29-oic acid | -80.73 | -95.78 |
| 12-Oleanene-3,16,21,23,28-pentol, (3β,16β,21β)-form, 16-Benzoyl | -79.91 | -103.75 | 3,22,24-Trihydroxy-12-oleanen-30-oic acid, (3β,22β)-form, 22-Ketone | -80.91 | -106.51 |
| 12-Oleanene-3,16,21,23,28-pentol, (3β,16β,21β)-form | -90.76 | -77.56 | 3,22,24-Trihydroxy-12-oleanen-30-oic acid, (3β,22β)-form | -92.03 | -72.28 |
| 12-Oleanene-3,16,21,23,28-pentol | -85.13 | -84.30 | ,22,24-Trihydroxy-12-oleanen-30-oic acid | -97.74 | -59.15 |
| 12-Oleanene-3,16,21,28-tetrol, (3β,16β,21β)-form | -91.91 | -55.78 | 3,22,27-Trihydroxy-12-oleanen-16-one, (3β,22α)-form | -93.74 | -97.23 |
| 12-Oleanene-3,16,21,28-tetrol | -89.75 | -81.79 | 3,22,27-Trihydroxy-12-oleanen-16-one | -93.96 | -97.42 |
| 12-Oleanene-3,16,21,30-tetrol, (3β,16β,21β)-form | -78.50 | -59.80 | 3,22,29-Trihydroxy-11-oxo-12-oleanen-30-oic acid, (3β,22β)-form | -91.49 | -104.22 |
| 12-Oleanene-3,16,21,30-tetrol | -71.35 | -19.10 | 3,22,29-Trihydroxy-11-oxo-12-oleanen-30-oic acid | -91.53 | -103.50 |
| 12-Oleanene-3,16,22,23,28-pentol, (3β,16α,22α)-form, 23-Aldehyde | -26.06 | -78.36 | 3,22,30-Trihydroxy-12-oleanen-11-one, (3β,22β)-form, 30-Carboxylic acid, 22-Ac | -65.01 | -17.17 |
| 12-Oleanene-3,16,22,23,28-pentol, (3β,16α,22α)-form | -64.12 | -87.77 | 3,22,30-Trihydroxy-12-oleanen-11-one, (3β,22β)-form, 30-Carboxylic acid, 30→22-lactone | -31.40 | -61.18 |

**Supplementary Table 5 Cont’d:** Docking energies of *epi*-Oleanolic acid analogues for *L. infantum* and *T. cruzi* CYP51.

| **Compounds** | ***T. cruzi* CYP51** | ***L. infantum* CYP51** | **Compounds** | ***T. cruzi* CYP51** | ***L. infantum* CYP51** |
| --- | --- | --- | --- | --- | --- |
| 12-Oleanene-3,16,22,23,28-pentol, (3β,16α,22β)-form, 22,28-Di-Ac | -74.59 | -71.67 | 3,22,30-Trihydroxy-12-oleanen-11-one, (3β,22β)-form | -86.83 | -98.58 |
| 12-Oleanene-3,16,22,23,28-pentol, (3β,16α,22β)-form | -25.04 | -81.84 | 3,22,30-Trihydroxy-12-oleanen-11-one | -87.59 | -98.58 |
| 12-Oleanene-3,16,22,23,28-pentol, (3β,16β,22α)-form | -31.06 | -87.80 | 3,22-Dihydroxy-11-oxo-12-oleanene-27,29-dioic acid, (3β,22α)-form, 29→22 Lactone, 27-Me ester | -103.01 | -79.78 |
| 12-Oleanene-3,16,22,23,28-pentol | -23.47 | -79.89 | 3,22-Dihydroxy-11-oxo-12-oleanene-27,29-dioic acid, (3β,22α)-form | -67.80 | -72.66 |
| 12-Oleanene-3,16,22,23-tetrol, (3β,16β,22α)-form | -87.09 | -80.71 | 3,22-Dihydroxy-11-oxo-12-oleanene-27,29-dioic acid | -67.60 | -71.70 |
| 12-Oleanene-3,16,22,23-tetrol | -86.47 | -77.64 | 3,22-Dihydroxy-12-oleanen-25-al, (3β,22β)-form | -55.76 | -89.65 |
| 12-Oleanene-3,16,22,24-tetrol, (3β,16β,22β)-form | -92.89 | -89.61 | 3,22-Dihydroxy-12-oleanen-25-al | -41.75 | -89.75 |
| 12-Oleanene-3,16,22,24-tetrol | -82.15 | -87.20 | 3,22-Dihydroxy-12-oleanen-28-oic acid, (3β,22α)-form | -91.23 | -80.09 |
| 12-Oleanene-3,16,22,28-tetrol, (3α,16α,22α)-form | -92.46 | -47.15 | 3,22-Dihydroxy-12-oleanen-28-oic acid, (3β,22β)-form, 22-Angeloyl, 3-Ac | -99.21 | -113.92 |
| 12-Oleanene-3,16,22,28-tetrol, (3β,16α,22α)-form, 16-Ac | -63.35 | 21.15 | 3,22-Dihydroxy-12-oleanen-28-oic acid, (3β,22β)-form, 22-Angeloyl | -90.92 | -47.13 |
| 12-Oleanene-3,16,22,28-tetrol, (3β,16α,22α)-form, 28-Aldehyde, 16-Ac. | -29.40 | -81.49 | 3,22-Dihydroxy-12-oleanen-28-oic acid, (3β,22β)-form | -87.11 | -32.23 |
| 12-Oleanene-3,16,22,28-tetrol, (3β,16α,22α)-form, 28-Aldehyde | -90.12 | -9.50 | 3,22-Dihydroxy-12-oleanen-28-oic acid. | -85.62 | -20.46 |
| 12-Oleanene-3,16,22,28-tetrol, (3β,16α,22α)-form | -79.04 | -46.29 | 3,22-Dihydroxy-12-oleanen-29-oic acid, (3α,22α)-form, 29→22 Lactone | -51.93 | -45.26 |
| 12-Oleanene-3,16,22,28-tetrol, (3β,16β,22β)-form, 22-Benzoyl | -103.90 | -96.70 | 3,22-Dihydroxy-12-oleanen-29-oic acid, (3α,22α)-form | -91.55 | -84.92 |
| 12-Oleanene-3,16,22,28-tetrol, (3β,16β,22β)-form | -94.50 | -54.07 | 3,22-Dihydroxy-12-oleanen-29-oic acid, (3α,22β)-form | -55.77 | -85.51 |
| 12-Oleanene-3,16,22,28-tetrol | -79.02 | -44.06 | 3,22-Dihydroxy-12-oleanen-29-oic acid, (3β,22α)-form, 22-Ketone | -54.98 | -84.63 |
| 12-Oleanene-3,16,22,28-tetrol, (3β,16α,22α)-form, 16-Angeloyl | -107.45 | 44.47 | 3,22-Dihydroxy-12-oleanen-29-oic acid, (3β,22α)-form, 29→22 Lactone | -80.15 | -58.18 |

**Supplementary Table 5 Cont’d:** Docking energies of *epi*-Oleanolic acid analogues for *L. infantum* and *T. cruzi* CYP51.

| **Compounds** | ***T. cruzi* CYP51** | ***L. infantum* CYP51** | **Compounds** | ***T. cruzi* CYP51** | ***L. infantum* CYP51** |
| --- | --- | --- | --- | --- | --- |
| 12-Oleanene-3,16,22,28-tetrol, (3β,16α,22α)-form, 16-E-Cinnamoyl | -91.18 | -99.08 | 3,22-Dihydroxy-12-oleanen-29-oic acid, (3β,22α)-form, Me ester | -84.05 | -56.12 |
| 12-Oleanene-3,16,22,28-tetrol, (3β,16α,22α)-form, 16-O-(3-Methyl-2-butenoyl) | -25.23 | -101.85 | 3,22-Dihydroxy-12-oleanen-29-oic acid, (3β,22α)-form | -55.94 | -85.57 |
| 12-Oleanene-3,16,22,28-tetrol, (3β,16α,22α)-form, 16-Tigloyl | -107.49 | 60.73 | 3,22-Dihydroxy-12-oleanen-29-oic acid,(3β,22β)-form | -55.66 | -71.49 |
| 12-Oleanene-3,16,22,28-tetrol, (3β,16α,22α)-form, 22-Angeloyl, 16-Ac | -25.01 | -99.41 | 3,22-Dihydroxy-12-oleanen-29-oic acid | -93.22 | -92.17 |
| 12-Oleanene-3,16,22,28-tetrol, (3β,16α,22α)-form, 22-Angeloyl | -90.00 | -108.01 | 3,22-Dihydroxy-23-oxo-12-oleanene-28,29-dioic acid, (3β,22α)-form | -95.19 | -43.52 |
| 12-Oleanene-3,16,22,28-tetrol, (3β,16α,22α)-form, 22-O-(2-Methylbutanoyl), 16-Ac | -75.30 | -104.50 | 3,22-Dihydroxy-23-oxo-12-oleanene-28,29-dioic acid | -103.99 | -40.00 |
| 12-Oleanene-3,16,22,28-tetrol, (3β,16α,22α)-form, 22-O-(3-Methyl-2-butenoyl) | -62.01 | -99.40 | 3,23,24-Trihydroxy-12-oleanen-28-oic acid, 3β-form | 14.68 | -93.79 |
| 12-Oleanene-3,16,22-triol, (3β,16β,22α)-form | -92.96 | -99.51 | 3,23,24-Trihydroxy-12-oleanen-28-oic acid | -27.36 | -99.89 |
| 12-Oleanene-3,16,22-triol, (3β,16β,22β)-form | -93.13 | -99.65 | 3,23,27,29-Tetrahydroxy-12-oleanen-28-oic acid, 3β-form | -92.90 | -104.28 |
| 12-Oleanene-3,16,22-triol | -93.14 | -99.62 | 3,23,27,29-Tetrahydroxy-12-oleanen-28-oic acid | -93.68 | -104.09 |
| 12-Oleanene-3,16,23,28-tetrol, (3β,16α)-form | -93.16 | -48.48 | 3,23,29-Trihydroxy-12-oleanen-28-oic acid, 3α-form, 23-Aldehyde | -93.41 | -105.71 |
| 12-Oleanene-3,16,23,28-tetrol, (3β,16β)-form | -75.14 | -55.21 | 3,23,29-Trihydroxy-12-oleanen-28-oic acid, 3α-form, 29-Carboxylic acid, 23-aldehyde | -93.62 | -72.01 |
| 12-Oleanene-3,16,23,28-tetrol | -75.98 | -55.84 | 3,23,29-Trihydroxy-12-oleanen-28-oic acid, 3β-form, Me ester | -53.46 | -94.08 |
| 12-Oleanene-3,16,28,29-tetrol, (3β,16α)-form, 29-Carboxylic acid | -74.09 | -57.04 | 3,23,29-Trihydroxy-12-oleanen-28-oic acid, 3β-form | -42.90 | -84.58 |
| 12-Oleanene-3,16,28,29-tetrol, (3β,16α)-form | -102.64 | -63.36 | 3,23,29-Trihydroxy-12-oleanen-28-oic acid | -91.29 | -70.61 |
| 12-Oleanene-3,16,28,29-tetrol, (3β,16β)-form | -91.00 | -95.87 | 3,23,29-Trihydroxy-12-oleanen-28-oic acid, 3α-form, 29-Carboxylic acid | -91.37 | -87.71 |
| 12-Oleanene-3,16,28,29-tetrol | -69.44 | -82.90 | 3,23,29-Trihydroxy-12-oleanen-28-oic acid, 3α-form | -44.10 | -103.32 |
| 12-Oleanene-3,16,28,30-tetrol, (3β,16α)-form, 30-Aldehyde | -95.85 | -48.11 | 3,23,29-Trihydroxy-12-oleanen-28-oic acid, 3β-form, 23-Aldehyde | -88.93 | -60.91 |

**Supplementary Table 5 Cont’d:** Docking energies of *epi*-Oleanolic acid analogues for *L. infantum* and *T. cruzi* CYP51.

| **Compounds** | ***T. cruzi* CYP51** | ***L. infantum* CYP51** | **Compounds** | ***T. cruzi* CYP51** | ***L. infantum* CYP51** |
| --- | --- | --- | --- | --- | --- |
| 12-Oleanene-3,16,28,30-tetrol, (3β,16α)-form | -7.54 | -79.02 | 3,23,29-Trihydroxy-12-oleanen-28-oic acid, 3β-form, 23-Carboxylic acid, 23-Me ester | -100.74 | -51.06 |
| 12-Oleanene-3,16,28,30-tetrol, (3β,16β)-form, 28-Aldehyde | -47.66 | -57.48 | 3,23,29-Trihydroxy-12-oleanen-28-oic acid, 3β-form, 23-Carboxylic acid. | -50.83 | -80.35 |
| 2-Oleanene-3,16,28,30-tetrol, (3β,16β)-form | -80.36 | -84.85 | 3,23,29-Trihydroxy-12-oleanen-28-oic acid, 3β-form, 29-Carboxylic acid, 23-aldehyde | -84.04 | -88.36 |
| 12-Oleanene-3,16,28,30-tetrol | -2.82 | -49.04 | 3,23,29-Trihydroxy-12-oleanen-28-oic acid, 3β-form, 29-Carboxylic acid | -75.98 | -57.69 |
| 2-Oleanene-3,16,28-triol, (3β,16α)-form, 16-Ketone, 3-Ac | -88.84 | -84.61 | 3,23,29-Trihydroxy-12-oleanen-28-oic acid, 3β-form, 3,23-Disulfate | -88.77 | -26.01 |
| 12-Oleanene-3,16,28-triol, (3β,16α)-form, 16-Ketone | -80.15 | -88.95 | 3,23-Dihydroxy-12-oleanen-27-oic acid, 3α-form, 3,23-Isopropylidene | -79.14 | -97.95 |
| 12-Oleanene-3,16,28-triol, (3β,16α)-form, 3,28-Di-Ac | -23.46 | -20.05 | 3,23-Dihydroxy-12-oleanen-27-oic acid, 3α-form, 23-Aldehyde | -78.22 | -79.32 |
| 12-Oleanene-3,16,28-triol, (3β,16α)-form | -93.95 | -14.41 | 3,23-Dihydroxy-12-oleanen-27-oic acid, 3α-form, 23-Carboxylic acid, 3-Ac | -106.22 | -84.13 |
| 12-Oleanene-3,16,28-triol, (3β,16β)-form, 28-Aldehyde | -71.93 | 3.52 | 3,23-Dihydroxy-12-oleanen-27-oic acid, 3α-form | -82.85 | -77.12 |
| 12-Oleanene-3,16,28-triol, (3β,16β)-form, 3-Hexadecanoyl | -109.41 | -142.66 | 3,23-Dihydroxy-12-oleanen-27-oic acid | -85.83 | -83.05 |
| 12-Oleanene-3,16,28-triol, (3β,16β)-form, 3-Ketone | -92.87 | -50.83 | 3,23-Dihydroxy-12-oleanen-28-oic acid, 3α-form | -86.37 | -62.91 |
| 12-Oleanene-3,16,28-triol, (3β,16β)-form | -94.06 | -14.66 | 3,23-Dihydroxy-12-oleanen-28-oic acid, 3β-form, 23-O-(3,4-Dihydroxy-E-cinnamoyl) | -127.37 | -117.67 |
| 12-Oleanene-3,16,28-triol | -43.69 | -97.09 | 3,23-Dihydroxy-12-oleanen-28-oic acid, 3β-form | -65.89 | -99.37 |
| 12-Oleanene-3,16-diol, (3α,16β)-form | -80.78 | -67.86 | 3,23-Dihydroxy-12-oleanen-28-oic acid | -88.91 | -89.41 |
| 2-Oleanene-3,16-diol, (3β,16α)-form | -80.92 | -68.31 | 3,23-Dihydroxy-12-oleanen-28-oic acid, 3β-form, 23-O-(4-Hydroxy-3-methoxy-E-cinnamoyl) | -130.95 | -126.99 |
| 12-Oleanene-3,16-diol, (3β,16β)-form, 3-Ac | -74.25 | -104.34 | 3,23-Dihydroxy-12-oleanen-28-oic acid, 3β-form, 23-O-(4-Hydroxy-3-methoxy-Z-cinnamoyl) | -128.51 | -116.87 |
| 12-Oleanene-3,16-diol, (3β,16β)-form, 3-Dodecanoyl | -140.40 | -137.05 | 3,23-Dihydroxy-12-oleanen-28-oic acid, 3β-form, 23-O-(4-Hydroxy-E-cinnamoyl) | -64.55 | -130.94 |
| 12-Oleanene-3,16-diol, (3β,16β)-form, 3-Eicosanoyl | -109.90 | -88.93 | (3,23-Dihydroxy-12-oleanen-28-oic acid, 3β-form, 23-O-(4-Hydroxy-Z-cinnamoyl) | -90.42 | -131.15 |

**Supplementary Table 5 Cont’d:** Docking energies of *epi*-Oleanolic acid analogues for *L. infantum* and *T. cruzi* CYP51.

| **Compounds** | ***T. cruzi* CYP51** | ***L. infantum* CYP51** | **Compounds** | ***T. cruzi* CYP51** | ***L. infantum* CYP51** |
| --- | --- | --- | --- | --- | --- |
| 12-Oleanene-3,16-diol, (3β,16β)-form, 3-Hexadecanoyl | -93.35 | -143.94 | 3,23-Dihydroxy-12-oleanen-28-oic acid, 3β-form, 3-O-(3,4-Dihydroxy-E-cinnamoyl) | -126.22 | -99.85 |
| 12-Oleanene-3,16-diol, (3β,16β)-form, 3-Octadecanoyl (Maniladiol stearate) | -120.95 | -154.97 | 3,23-Dihydroxy-12-oleanen-28-oic acid, 3β-form, 3-O-(4-Hydroxy-Z-cinnamoyl) | -50.57 | -131.39 |
| 12-Oleanene-3,16-diol, (3β,16β)-form, 3-Tetradecanoyl | -132.02 | -138.87 | 3,23-Dihydroxy-12-oleanen-28-oic acid, 3β-form, 3-Sulfate | -52.53 | -100.04 |
| 12-Oleanene-3,16-diol, (3β,16β)-form | -80.59 | -67.82 | 3,23-Dihydroxy-12-oleanen-28-oic acid, 3β-form, Di-Ac, Me ester | -90.27 | -112.38 |
| 12-Oleanene-3,16-diol | -82.94 | -29.62 | 3,23-Dihydroxy-12-oleanen-29-oic acid, 3α-form, 23-Carboxylic acid, 3-Ac, 29-Me ester. | -89.79 | -36.00 |
| 12-Oleanene-3,19,24-triol, (3β,19β)-form | -93.84 | -93.25 | 3,23-Dihydroxy-12-oleanen-29-oic acid, 3α-form, 23-Carboxylic acid, 3-Ac | -90.01 | -97.59 |
| 12-Oleanene-3,19,24-triol | -103.24 | -89.29 | 3,23-Dihydroxy-12-oleanen-29-oic acid, 3α-form, 23-Carboxylic acid | -90.30 | -88.31 |
| 12-Oleanene-3,19,28-triol, (3β,19α)-form | -79.65 | -90.52 | 3,23-Dihydroxy-12-oleanen-29-oic acid, 3α-form | -56.81 | -85.81 |
| 12-Oleanene-3,19,28-triol | -86.86 | -87.22 | 3,23-Dihydroxy-12-oleanen-29-oic acid, 3β-form, 23-Carboxylic acid, 29-Me ester | -106.47 | -95.34 |
| 12-Oleanene-3,21,22,23,29-pentol, (3β,21β,22β)-form | -37.05 | -64.71 | 3,23-Dihydroxy-12-oleanen-29-oic acid, 3β-form, 23-Carboxylic acid | -88.77 | -89.18 |
| 12-Oleanene-3,21,22,23,29-pentol | -85.71 | 21.77 | 3,23-Dihydroxy-12-oleanen-29-oic acid, 3β-form | -86.07 | -85.82 |
| 12-Oleanene-3,21,22,24,28-pentol, (3β,21β,22α)-form, 28-Carboxylic acid | -83.58 | -60.95 | 3,23-Dihydroxy-12-oleanen-29-oic acid | -57.31 | -85.84 |
| 12-Oleanene-3,21,22,24,28-pentol, (3β,21β,22α)-form | -99.61 | -69.21 | 3,23-Dihydroxy-22-oxo-12-oleanen-29-oic acid, 3β-form | -88.32 | -41.23 |
| 12-Oleanene-3,21,22,24,28-pentol | -99.07 | -65.15 | 3,23-Dihydroxy-22-oxo-12-oleanen-29-oic acid | -91.66 | -47.29 |
| 12-Oleanene-3,21,22,24,29-pentol, (3β,21α,22β)-form | -61.79 | -51.09 | 3,23-Epoxy-12-oleanen-28-oic acid, 3α-form | -84.59 | -79.97 |
| 12-Oleanene-3,21,22,24,29-pentol, (3β,21β,22β)-form | -83.80 | -52.26 | 3,23-Epoxy-12-oleanen-28-oic acid, 3β-form | -82.76 | -99.12 |
| 12-Oleanene-3,21,22,24,29-pentol | -84.38 | -52.12 | 3,23-Epoxy-12-oleanen-28-oic acid | -84.70 | -80.87 |
| 12-Oleanene-3,21,22,24,30-pentol, (3β,21β,22β)-form | -89.00 | -83.57 | 3,24,29,30-Tetrahydroxy-12-oleanen-22-one, 3β-form | -91.22 | -104.81 |
| 12-Oleanene-3,21,22,24,30-pentol | -89.45 | -83.43 | 3,24,29,30-Tetrahydroxy-12-oleanen-22-one | -79.19 | -91.28 |

**Supplementary Table 5 Cont’d:** Docking energies of *epi*-Oleanolic acid analogues for *L. infantum* and *T. cruzi* CYP51.

| **Compounds** | ***T. cruzi* CYP51** | ***L. infantum* CYP51** | **Compounds** | ***T. cruzi* CYP51** | ***L. infantum* CYP51** |
| --- | --- | --- | --- | --- | --- |
| 12-Oleanene-3,21,22,24-tetrol, (3β,21α,22β)-form | -69.78 | -61.54 | 3,24,29-Trihydroxy-12-oleanen-22-one, 3β-form | -38.11 | -72.36 |
| 12-Oleanene-3,21,22,24-tetrol, (3β,21β,22β)-form | -58.74 | -95.41 | 3,24,29-Trihydroxy-12-oleanen-22-one | -38.44 | -72.22 |
| 12-Oleanene-3,21,22,24-tetrol | -58.48 | -95.45 | 3,24-Dihydroxy-11-oxo-12-oleanen-29-oic acid, 3β-form, Di-Ac, Me ester | -110.71 | -11.55 |
| 12-Oleanene-3,21,22,28-tetrol, (3α,21α,22α)-form | -87.81 | -95.81 | 3,24-Dihydroxy-11-oxo-12-oleanen-29-oic acid, 3β-form | -76.49 | -47.95 |
| 12-Oleanene-3,21,22,28-tetrol, (3β,21β,22α)-form | -87.87 | -95.24 | 3,24-Dihydroxy-11-oxo-12-oleanen-29-oic acid | -85.80 | -58.63 |
| 12-Oleanene-3,21,22,28-tetrol | -87.76 | -97.96 | 3,24-Dihydroxy-11-oxo-12-oleanen-30-oic acid, 3β-form, Di-Ac, Me ester | -104.33 | -99.29 |
| 12-Oleanene-3,21,22,29-tetrol, (3β,21β,22β)-form, 29-Carboxylic acid. | -77.60 | -97.93 | 3,24-Dihydroxy-11-oxo-12-oleanen-30-oic acid, 3β-form, Di-Ac | -113.92 | -108.01 |
| 2-Oleanene-3,21,22,29-tetrol, (3β,21β,22β)-form | -81.48 | -106.61 | 3,24-Dihydroxy-11-oxo-12-oleanen-30-oic acid, 3β-form, Me ester | -81.90 | -89.79 |
| 12-Oleanene-3,21,22,29-tetrol | -91.04 | -92.02 | 3,24-Dihydroxy-11-oxo-12-oleanen-30-oic acid, 3β-form | -96.30 | -52.02 |
| 12-Oleanene-3,21,22-triol, (3β,21β,22β)-form | -43.35 | -61.22 | 3,24-Dihydroxy-11-oxo-12-oleanen-30-oic acid | -97.51 | -50.09 |
| 12-Oleanene-3,21,22-triol | -45.50 | -66.06 | 3,24-Dihydroxy-12-oleanen-27-oic acid, 3α-form | -67.25 | -90.03 |
| 12-Oleanene-3,21,24-triol, (3β,21α)-form | -73.03 | -31.32 | 3,24-Dihydroxy-12-oleanen-27-oic acid | -67.04 | -90.36 |
| 12-Oleanene-3,21,24-triol, (3β,21β)-form | -89.42 | -90.80 | 3,24-Dihydroxy-12-oleanen-28-oic acid, 3α-form, 24-Aldehyde | -65.44 | -68.73 |
| 12-Oleanene-3,21,24-triol. | -88.24 | -64.61 | ,24-Dihydroxy-12-oleanen-28-oic acid, 3α-form | -70.67 | -95.74 |
| 12-Oleanene-3,21,28-triol, (3β,21β)-form | -92.88 | -72.26 | 3,24-Dihydroxy-12-oleanen-28-oic acid, 3β-form, 24-Aldehyde | -35.88 | -65.80 |
| 12-Oleanene-3,21,28-triol | -92.86 | -72.16 | 3,24-Dihydroxy-12-oleanen-28-oic acid, 3β-form, 24-O-(4-Hydroxy-E-cinnamoyl) | -134.10 | -131.53 |
| 12-Oleanene-3,21-diol, (3α,21β)-form | -63.52 | -65.30 | 3,24-Dihydroxy-12-oleanen-28-oic acid, 3β-form | -69.86 | -94.91 |
| 12-Oleanene-3,21-diol, (3β,21α)-form | -63.52 | -64.00 | 3,24-Dihydroxy-12-oleanen-28-oic acid | -69.52 | -95.37 |
| 12-Oleanene-3,21-diol, (3β,21β)-form | -64.64 | -76.87 | 3,24-Dihydroxy-12-oleanen-29-oic acid, 3β-form, Me ester | -85.47 | -112.78 |
| 12-Oleanene-3,21-diol, 21β-form | -60.47 | -65.12 | 3,24-Dihydroxy-12-oleanen-29-oic acid, 3β-form | -83.37 | -82.45 |
| 12-Oleanene-3,21-diol | -63.73 | -65.53 | 3,24-Dihydroxy-12-oleanen-29-oic acid | -81.40 | -82.21 |

**Supplementary Table 5 Cont’d:** Docking energies of *epi*-Oleanolic acid analogues for *L. infantum* and *T. cruzi* CYP51.

| **Compounds** | ***T. cruzi* CYP51** | ***L. infantum* CYP51** | **Compounds** | ***T. cruzi* CYP51** | ***L. infantum* CYP51** |
| --- | --- | --- | --- | --- | --- |
| 12-Oleanene-3,22,23,29-tetrol, (3β,22β)-form | -87.12 | -40.67 | 3,24-Dihydroxy-12-oleanen-30-oic acid, 3β-form | -103.41 | -111.65 |
| 12-Oleanene-3,22,23,29-tetrol | -87.47 | -42.34 | 3,24-Dihydroxy-12-oleanen-30-oic acid | -103.43 | -111.58 |
| 12-Oleanene-3,22,24,29-tetrol, (3β,22α)-form | -86.81 | -43.34 | 3,24-Dihydroxy-12-oleanene-28,30-dioic acid, 3α-form | -52.00 | -27.11 |
| 12-Oleanene-3,22,24,29-tetrol, (3β,22β)-form | -86.67 | -40.23 | 3,24-Dihydroxy-12-oleanene-28,30-dioic acid | -90.46 | -13.44 |
| 12-Oleanene-3,22,24,29-tetrol | -79.43 | -101.20 | 3,24-Dihydroxy-16-oxo-12-oleanen-29-oic acid, 3β-form | -85.19 | -103.03 |
| 12-Oleanene-3,22,24,30-tetrol, (3β,22β)-form | -73.00 | -55.30 | 3,24-Dihydroxy-16-oxo-12-oleanen-29-oic acid | -84.79 | -102.50 |
| 12-Oleanene-3,22,24,30-tetrol | -75.66 | -49.43 | 3,24-Dihydroxy-22-oxo-12-oleanen-29-oic acid, 3β-form | -95.83 | -43.53 |
| 12-Oleanene-3,22,24-triol, (3β,22α)-form | -77.05 | -68.65 | 3,24-Dihydroxy-22-oxo-12-oleanen-29-oic acid | -96.12 | -45.94 |
| 12-Oleanene-3,22,24-triol, (3β,22β)-form, 24-Aldehyde | -72.07 | -62.84 | 3,25-Dihydroxy-12-oleanen-28-oic acid, 3α-form, 3-Ac | -98.67 | -93.03 |
| 12-Oleanene-3,22,24-triol, (3β,22β)-form | -72.76 | -55.34 | 3,25-Dihydroxy-12-oleanen-28-oic acid, 3α-form | -91.93 | -70.54 |
| 12-Oleanene-3,22,24-triol | -74.22 | -52.25 | 3,25-Dihydroxy-12-oleanen-28-oic acid | -92.23 | -69.58 |
| 12-Oleanene-3,22,28-triol, (3β,22α)-form | -81.07 | -84.47 | 3,25-Dihydroxy-12-oleanen-30-oic acid, 3β-form, 25-Aldehyde | -75.68 | -95.65 |
| 12-Oleanene-3,22,28-triol | -80.37 | -84.55 | 3,25-Dihydroxy-12-oleanen-30-oic acid, 3β-form | -89.08 | -82.91 |
| 12-Oleanene-3,22,29-triol, (3β,22β)-form | -85.17 | -70.08 | 3,25-Dihydroxy-12-oleanen-30-oic acid | -49.15 | -83.48 |
| 12-Oleanene-3,22,29-triol | -82.21 | -64.61 | 3,25-Epoxy-2,3,7-trihydroxy-12-oleanen-28-oic acid, (2β,3αOH,7β)-form | -85.53 | -72.88 |
| 12-Oleanene-3,22,30-triol, (3β,22α)-form, 30-Carboxylic acid | -85.31 | -89.78 | 3,25-Epoxy-2,3,7-trihydroxy-12-oleanen-28-oic acid | -85.62 | -73.27 |
| 12-Oleanene-3,22,30-triol, (3β,22α)-form | -87.35 | -79.50 | 3,25-Epoxy-3,21,22-trihydroxy-12-oleanen-28-oic acid, (3αOH,21α,22β)-form | -84.49 | -84.12 |
| 12-Oleanene-3,22,30-triol, (3β,22β)-form, 22-Ketone | -70.98 | -79.33 | 3,25-Epoxy-3,21,22-trihydroxy-12-oleanen-28-oic acid, 22-(3-Methyl-2-butenoyl) | -96.84 | -100.97 |
| 12-Oleanene-3,22,30-triol, (3β,22β)-form, 30-Carboxylic acid, 22-ketone | -96.00 | -61.01 | 3,25-Epoxy-3,21,22-trihydroxy-12-oleanen-28-oic acid, 22-Angeloyl | -98.11 | -99.48 |
| 12-Oleanene-3,22,30-triol, (3β,22β)-form, 30-Carboxylic acid, 30→22 lactone | -90.25 | -64.05 | 3,25-Epoxy-3,21,22-trihydroxy-12-oleanen-28-oic acid, 22-Tigloyl | -105.44 | -102.77 |

**Supplementary Table 5 Cont’d:** Docking energies of *epi*-Oleanolic acid analogues for *L. infantum* and *T. cruzi* CYP51.

| **Compounds** | ***T. cruzi* CYP51** | ***L. infantum* CYP51** | **Compounds** | ***T. cruzi* CYP51** | ***L. infantum* CYP51** |
| --- | --- | --- | --- | --- | --- |
| 12-Oleanene-3,22,30-triol, (3β,22β)-form | -67.97 | -26.41 | 3,25-Epoxy-3,21,22-trihydroxy-12-oleanen-28-oic acid | -73.63 | -83.60 |
| 12-Oleanene-3,22,30-triol | -80.75 | -76.78 | 3,25-Epoxy-3,22-dihydroxy-11-oxo-12-oleanen-28-oic acid, (22β)-form, 22-O-(3-Methyl-2-butenoyl) | -99.16 | -44.48 |
| 12-Oleanene-3,22-diol, (3α,22β)-form | -50.28 | -46.33 | 3,25-Epoxy-3,22-dihydroxy-11-oxo-12-oleanen-28-oic acid, (22β)-form | -79.61 | -69.73 |
| 12-Oleanene-3,22-diol, (3β,22α)-form | -18.65 | -44.98 | 3,25-Epoxy-3,22-dihydroxy-11-oxo-12-oleanen-28-oic acid | -70.60 | -84.46 |
| 12-Oleanene-3,22-diol, (3β,22β)-form, 22-Ketone | -88.56 | -25.73 | 3,25-Epoxy-3,22-dihydroxy-12-oleanen-28-oic acid, (22β)-form, 22-Angeloyl | -103.09 | -113.49 |
| 12-Oleanene-3,22-diol, (3β,22β)-form | -80.78 | -71.32 | ,25-Epoxy-3,22-dihydroxy-12-oleanen-28-oic acid, (22β)-form, 22-O-(2-Methylbutanoyl) | -91.39 | -110.34 |
| 12-Oleanene-3,22-diol | 16.96 | -45.67 | 3,25-Epoxy-3,22-dihydroxy-12-oleanen-28-oic acid, (22β)-form, 22-O-(3-Methyl-2-butenoyl) | -97.18 | -107.50 |
| 12-Oleanene-3,23,28,30-tetrol, 3α-form, 28-Carboxylic acid, 23-aldehyde | -96.44 | -95.09 | 3,25-Epoxy-3,22-dihydroxy-12-oleanen-28-oic acid, (22β)-form, 22-O-(3-Methyl-2-butenoyl) | -103.70 | -101.41 |
| 12-Oleanene-3,23,28,30-tetrol, 3α-form | -80.17 | -82.84 | 3,25-Epoxy-3,22-dihydroxy-12-oleanen-28-oic acid, (22β)-form, 22-Tigloyl | -109.22 | -97.77 |
| 12-Oleanene-3,23,28,30-tetrol, 3β-form, 23,28-Dicarboxylic acid | -75.66 | -98.80 | 3,25-Epoxy-3,22-dihydroxy-12-oleanen-28-oic acid, (22β)-form, 22-Tigloyl | -102.35 | -108.56 |
| 2-Oleanene-3,23,28,30-tetrol, 3β-form, 28,30-Dicarboxylic acid, 3,23-di-Ac, 28-Me ester | -43.06 | -94.31 | 3,25-Epoxy-3,22-dihydroxy-12-oleanen-28-oic acid, (22β)-form, 3-Me ether, 22-angeloyl | -105.81 | -112.65 |
| 12-Oleanene-3,23,28,30-tetrol, 3β-form, 28,30-Dicarboxylic acid, 30-Me ester | -47.60 | -71.03 | 3,25-Epoxy-3,22-dihydroxy-12-oleanen-28-oic acid, (22β)-form | -74.47 | -65.97 |
| 12-Oleanene-3,23,28,30-tetrol, 3β-form, 28,30-Dicarboxylic acid | -44.92 | -79.65 | 3,25-Epoxy-3,22-dihydroxy-12-oleanen-28-oic acid | -74.88 | -65.07 |
| 12-Oleanene-3,23,28,30-tetrol, 3β-form, 28-Carboxylic acid, 30-aldehyde | -99.37 | -62.25 | 3,25-Epoxy-3-hydroxy-11-oxo-12-oleanen-28-oic acid | -98.72 | -84.46 |
| 12-Oleanene-3,23,28,30-tetrol, 3β-form | -80.15 | -93.72 | 3,25-Epoxy-3-hydroxy-12-oleanen-28-oic acid, 28-Alcohol | -89.08 | -90.88 |
| 12-Oleanene-3,23,28,30-tetrol. | -77.01 | -52.97 | 3,25-Epoxy-3-hydroxy-12-oleanen-28-oic acid, 28-Aldehyde | -88.66 | -88.07 |
| 12-Oleanene-3,23,28-triol, 3β-form, 23-O-(3,4-Dihydroxy-E-cinnamoyl) | -107.36 | -96.72 | 3,25-Epoxy-3-hydroxy-12-oleanen-28-oic acid, Et ether | -95.20 | -88.25 |

**Supplementary Table 5 Cont’d:** Docking energies of *epi*-Oleanolic acid analogues for *L. infantum* and *T. cruzi* CYP51.

| **Compounds** | ***T. cruzi* CYP51** | ***L. infantum* CYP51** | **Compounds** | ***T. cruzi* CYP51** | ***L. infantum* CYP51** |
| --- | --- | --- | --- | --- | --- |
| 12-Oleanene-3,23,28-triol, 3β-form, 3-O-(3,4-Dihydroxy-E-cinnamoyl) | -60.40 | -106.90 | 3,25-Epoxy-3-hydroxy-12-oleanen-28-oic acid | -77.83 | -92.41 |
| 12-Oleanene-3,23,28-triol, 3β-form, 3-O-(2-Hydroxyacetyl) | -88.11 | -72.88 | 3,26-Dihydroxy-12-oleanen-28-oic acid, 3α-form | -83.27 | -86.96 |
| 12-Oleanene-3,23,28-triol, 3β-form | -78.39 | -81.47 | 3,26-Dihydroxy-12-oleanen-28-oic acid | -83.63 | -86.72 |
| 12-Oleanene-3,23,28-triol | -75.33 | -79.28 | 3,27-Dihydroxy-11-oxo-12-oleanen-28-oic acid, 3β-form | -46.22 | -50.92 |
| 12-Oleanene-3,23-diol, 3α-form | -86.88 | -74.87 | 3,27-Dihydroxy-11-oxo-12-oleanen-28-oic acid | -46.13 | -50.47 |
| 12-Oleanene-3,23-diol, 3β-form | -83.70 | -80.78 | 3,27-Dihydroxy-12-oleanen-28-oic acid, 3α-form, 27-O-(3,4-Dihydroxy-E-cinnamoyl), 3-Ac, Me ester | -92.01 | -95.21 |
| 12-Oleanene-3,23-diol | -83.48 | -80.06 | 3,27-Dihydroxy-12-oleanen-28-oic acid, 3α-form | -43.52 | -50.49 |
| 12-Oleanene-3,24,28,29-tetrol, 3α-form, 28-Carboxylic acid | -94.75 | -95.64 | 3,27-Dihydroxy-12-oleanen-28-oic acid, 3β-form, 27-O-(4-Hydroxy-Z-cinnamoyl) | -55.19 | -115.55 |
| 12-Oleanene-3,24,28,29-tetrol, 3α-form | -78.00 | -93.62 | 3,27-Dihydroxy-12-oleanen-28-oic acid, 3β-form, 27-O-(4-Hydroxybenzoyl), 3-Ac, Me ester | -109.65 | -112.20 |
| 12-Oleanene-3,24,28,29-tetrol, 3β-form, 24,28-Dicarboxylic acid | -42.85 | -74.93 | 3,27-Dihydroxy-12-oleanen-28-oic acid, 3β-form, 3,27-Bis-O-(3,4-dihydroxy-E-cinnamoyl) | -60.82 | -105.34 |
| 12-Oleanene-3,24,28,29-tetrol, 3β-form, 28,29-Dicarboxylic acid | -95.72 | -68.05 | 3,27-Dihydroxy-12-oleanen-28-oic acid, 3β-form, 3,27-Bis-O-(4-hydroxy-E-cinnamoyl) | -88.98 | -139.91 |
| 12-Oleanene-3,24,28,29-tetrol, 3β-form | -76.08 | -68.55 | 3,27-Dihydroxy-12-oleanen-28-oic acid, 3β-form, 3-O-(4-Hydroxy-E-cinnamoyl), 27-O-(4-hydroxy-Z-cinnamoyl) (Asprellic acid B) | -76.69 | -149.05 |
| 12-Oleanene-3,24,28,29-tetrol | -76.91 | -92.95 | 3,27-Dihydroxy-12-oleanen-28-oic acid, 3β-form, 3-O-(4-Hydroxy-Z-cinnamoyl), 27-O-(4-hydroxy-E-cinnamoyl) | -120.51 | -118.23 |
| 12-Oleanene-3,24-diol, 3α-form, 24-Carboxylic acid, Ac | -83.33 | -77.53 | 3,27-Dihydroxy-12-oleanen-28-oic acid, 3β-form, 3-Sulfate | -95.33 | -83.07 |
| 12-Oleanene-3,24-diol, 3α-form, 24-Carboxylic acid, Ac | -94.94 | -86.61 | 3,27-Dihydroxy-12-oleanen-28-oic acid, 3β-form, Me ester | -51.64 | -85.24 |
| 12-Oleanene-3,24-diol, 3α-form. | -84.83 | -84.36 | 3,27-Dihydroxy-12-oleanen-28-oic acid, 3β-form, Me ester | -35.75 | -77.88 |
| 12-Oleanene-3,24-diol, 3β-form | -84.88 | -84.21 | 3,27-Dihydroxy-12-oleanen-28-oic acid, 3β-form | -92.15 | -37.31 |

**Supplementary Table 5 Cont’d:** Docking energies of *epi*-Oleanolic acid analogues for *L. infantum* and *T. cruzi* CYP51.

| **Compounds** | ***T. cruzi* CYP51** | ***L. infantum* CYP51** | **Compounds** | ***T. cruzi* CYP51** | ***L. infantum* CYP51** |
| --- | --- | --- | --- | --- | --- |
| 12-Oleanene-3,24-diol | -84.44 | -81.19 | 3,27-Dihydroxy-12-oleanen-28-oic acid, 3β-form, 27-Aldehyde | -67.37 | -67.99 |
| 12-Oleanene-3,27-diol, 3α-form | -84.99 | -93.67 | 3,27-Dihydroxy-12-oleanen-28-oic acid | -91.95 | -37.34 |
| 12-Oleanene-3,27-diol, 3β-form | -87.41 | -30.91 | 3,27-Dihydroxy-12-oleanen-28-oic acid, 3β-form, 27-Benzoyl, 3-Ac, Me ester | -16.21 | -101.68 |
| 12-Oleanene-3,27-diol | -87.22 | -30.87 | 3,27-Dihydroxy-12-oleanen-28-oic acid, 3β-form, 27-Benzoyl, Me ester | -37.95 | -85.60 |
| 12-Oleanene-3,28,29-triol, 3β-form | -96.03 | -79.73 | 3,27-Dihydroxy-12-oleanen-28-oic acid, 3β-form, 27-O-(3,4-Dihydroxy-E-cinnamoyl), 3-O-(4-hydroxy-E-cinnamoyl). | -63.25 | -132.64 |
| 2-Oleanene-3,28,29-triol | -96.11 | -80.09 | 3,27-Dihydroxy-12-oleanen-28-oic acid, 3β-form, 27-O-(3,4-Dihydroxy-E-cinnamoyl) | -61.57 | -114.86 |
| 12-Oleanene-3,28,30-triol, 3β-form, 28-Carboxylic acid, 30-O-(3,4-dihydroxycinnamoyl) | -89.86 | -141.39 | 3,27-Dihydroxy-12-oleanen-28-oic acid, 3β-form, 27-O-(4-Hydroxy-3-methoxy-E-cinnamoyl) | -98.83 | -117.37 |
| 12-Oleanene-3,28,30-triol, 3β-form, 28-Carboxylic acid, 30-aldehyde | -95.23 | -93.12 | 3,27-Dihydroxy-12-oleanen-28-oic acid, 3β-form, 27-O-(4-Hydroxy-3-methoxy-Z-cinnamoyl) | -101.65 | -118.15 |
| 12-Oleanene-3,28,30-triol, 3β-form, 28-Carboxylic acid | -94.64 | -55.56 | 3,27-Dihydroxy-12-oleanen-28-oic acid, 3β-form, 27-O-(4-Hydroxy-E-cinnamoyl) | -55.33 | -113.79 |
| 12-Oleanene-3,28,30-triol, 3β-form. | -110.17 | -90.77 | 3,27-Dihydroxy-2-oxo-12-oleanene-23,28-dioic acid, 3β-form | -111.66 | -81.79 |
| 12-Oleanene-3,28,30-triol | -104.63 | -104.76 | 3,27-Dihydroxy-2-oxo-12-oleanene-23,28-dioic acid | -111.79 | -82.61 |
| 12-Oleanene-3,28-diol, 3α-form | 93.11 | 97.44 | 3,28-Dihydroxy-11-oxo-12-oleanen-30-oic acid, 3β-form, Di-Ac, Me ester | -77.31 | -101.45 |
| 12-Oleanene-3,28-diol, 3β-form, 28-(4-Hydroxyphenyl) ether | -41.80 | -112.53 | 3,28-Dihydroxy-12-oleanen-29-oic acid, 3α-form | -78.37 | -90.91 |
| 12-Oleanene-3,28-diol, 3β-form, 3-Decanoyl | -39.79 | -106.44 | 3,28-Dihydroxy-12-oleanen-29-oic acid, 3β-form | -78.57 | -91.16 |
| 12-Oleanene-3,28-diol, 3β-form, 3-Dotriacontanoyl | 22.80 | -37.92 | 3,28-Dihydroxy-12-oleanen-29-oic acid | -77.66 | -90.63 |
| 12-Oleanene-3,28-diol, 3β-form, 3-Hexadecanoyl | -125.82 | -120.36 | 3,29-Dihydroxy-12-oleanen-27-oic acid, 3α-form, 29-Aldehyde | -102.99 | -101.31 |
| 12-Oleanene-3,28-diol, 3β-form, 3-Hexatriacontanoyl | -55.15 | -78.42 | 3,29-Dihydroxy-12-oleanen-27-oic acid, 3α-form, 29-Carboxylic acid | -105.85 | -92.43 |

**Supplementary Table 5 Cont’d:** Docking energies of *epi*-Oleanolic acid analogues for *L. infantum* and *T. cruzi* CYP51.

| **Compounds** | ***T. cruzi* CYP51** | ***L. infantum* CYP51** | **Compounds** | ***T. cruzi* CYP51** | ***L. infantum* CYP51** |
| --- | --- | --- | --- | --- | --- |
| 12-Oleanene-3,28-diol, 3β-form, 3-O-(9Z,12Z-Octadecadienoyl) | -142.74 | -133.48 | 3,29-Dihydroxy-12-oleanen-27-oic acid, 3α-form | -102.84 | -91.72 |
| 12-Oleanene-3,28-diol, 3β-form, 3-Octacosanoyl | -51.70 | -95.78 | 3,29-Dihydroxy-12-oleanen-27-oic acid | -102.78 | -89.12 |
| 12-Oleanene-3,28-diol, 3β-form, 3-Octadecanoyl (Erythrodiol 3-stearate) | -126.00 | -146.54 | 3,29-Dihydroxy-12-oleanen-28-oic acid, 3α-form, 3-Ac | -109.65 | -118.18 |
| 12-Oleanene-3,28-diol, 3β-form, 3-Tetratriacontanoyl | 76.97 | -85.53 | 3,29-Dihydroxy-12-oleanen-28-oic acid, 3α-form | -96.12 | -110.78 |
| 12-Oleanene-3,28-diol, 3β-form, 3-Triacontanoyl | -81.69 | -68.10 | 3,29-Dihydroxy-12-oleanen-28-oic acid, 3β-form, 29-Aldehyde | -107.38 | -99.84 |
| 12-Oleanene-3,28-diol, 3β-form, Di-Ac | -43.18 | -99.92 | 3,29-Dihydroxy-12-oleanen-28-oic acid, 3β-form, 3-Ac | -100.72 | -99.28 |
| 12-Oleanene-3,28-diol, 3β-form, 28-Ac | -79.30 | -97.32 | 3,29-Dihydroxy-12-oleanen-28-oic acid, 3β-form | -96.05 | -110.72 |
| 12-Oleanene-3,28-diol, 3β-form | 94.07 | 97.25 | 3,29-Dihydroxy-12-oleanen-28-oic acid | -95.10 | -107.42 |
| 12-Oleanene-3,28-diol | 95.88 | 97.21 | 3,30-Dihydroxy-12-oleanen-29-oic acid, 3β-form, Me ester | -105.83 | -79.34 |
| 12-Oleanene-3,28-diol, 3β-form, 28-Aldehyde, 3-(3,4-dihydroxy-E-cinnamoyl) | -129.14 | -124.23 | 3,30-Dihydroxy-12-oleanen-29-oic acid, 3β-form | -105.81 | -102.80 |
| 12-Oleanene-3,28-diol, 3β-form, 28-Aldehyde, 3-Ac | -73.18 | -74.23 | 3,30-Dihydroxy-12-oleanen-29-oic acid | -101.00 | -59.14 |
| 12-Oleanene-3,28-diol, 3β-form, 28-Aldehyde | -48.90 | -82.77 | 3,5,25-Trihydroxy-12-oleanene-23,28-dioic acid, (3α,5α)-form | -47.28 | -85.05 |
| 12-Oleanene-3,28-diol, 3β-form, 3-(3,4-Dihydroxy-E-cinnamoyl) | -129.66 | -128.91 | 3,5,25-Trihydroxy-12-oleanene-23,28-dioic acid | -74.92 | -74.46 |
| 12-Oleanene-3,28-diol, 3β-form, 3-(4-Hydroxy-E-cinnamoyl) | 26.11 | 105.22 | 3,6,11,23,24-Pentahydroxy-12-oleanen-28-oic acid, (3β,6β,11β)-form | -91.68 | -114.25 |
| 12-Oleanene-3,28-diol, 3β-form, 3-(4-Hydroxy-Z-cinnamoyl) | 31.69 | 72.28 | 3,6,11,23,24-Pentahydroxy-12-oleanen-28-oic acid | -99.45 | -91.49 |
| 12-Oleanene-3,28-diol, 3β-form, 3-Ac | -33.43 | -90.54 | 3,6,16,23-Tetrahydroxy-12-oleanen-28-oic acid, (3β,6β,16α)-form | -91.73 | -72.46 |
| 12-Oleanene-3,29-diol, 3β-form | -82.53 | -37.07 | 3,6,16,23-Tetrahydroxy-12-oleanen-28-oic acid | -91.62 | -73.92 |
| 12-Oleanene-3,29-diol | -89.00 | -93.84 | 3,6,19,23-Tetrahydroxy-12-oleanen-28-oic acid, (3β,6β,19α)-form, 6-Ketone | -103.31 | -92.65 |
| 12-Oleanene-3,6,16,21,23-pentol, (3β,6β,16β,21β)-form | -97.96 | -91.52 | 3,6,19,23-Tetrahydroxy-12-oleanen-28-oic acid, (3β,6β,19α)-form | -85.70 | 42.20 |

**Supplementary Table 5 Cont’d:** Docking energies of *epi*-Oleanolic acid analogues for *L. infantum* and *T. cruzi* CYP51.

| **Compounds** | ***T. cruzi* CYP51** | ***L. infantum* CYP51** | **Compounds** | ***T. cruzi* CYP51** | ***L. infantum* CYP51** |
| --- | --- | --- | --- | --- | --- |
| 12-Oleanene-3,6,16,21,23-pentol | -97.96 | -91.28 | 3,6,19,23-Tetrahydroxy-12-oleanen-28-oic acid | -22.54 | -48.25 |
| 12-Oleanene-3,6,16,21-tetrol, (3β,6β,16β,21β)-form | -103.07 | -57.40 | 3,6,21,23-Tetrahydroxy-12-oleanen-28-oic acid, (3β,6α,21β)-form | -90.50 | -30.51 |
| 12-Oleanene-3,6,16,21-tetrol | -97.05 | -60.23 | 3,6,21,23-Tetrahydroxy-12-oleanen-28-oic acid | -90.48 | -30.56 |
| 12-Oleanene-3,6,16,23-tetrol, (3β,6β,16β)-form | -104.13 | -40.59 | 3,6,22-Trihydroxy-12-oleanen-28-oic acid, (3β,6β,22α)-form | -70.07 | -77.76 |
| 12-Oleanene-3,6,16,23-tetrol | -104.06 | -47.04 | 3,6,22-Trihydroxy-12-oleanen-28-oic acid | -80.93 | -85.78 |
| 12-Oleanene-3,6,16,28-tetrol, (3β,6β,16α)-form, 16-Ac | -69.37 | -1.93 | 3,6,24-Trihydroxy-12-oleanen-27-oic acid, (3β,6β)-form | -87.41 | -90.61 |
| 12-Oleanene-3,6,16,28-tetrol, (3β,6β,16α)-form | -88.41 | -75.37 | 3,6,24-Trihydroxy-12-oleanen-27-oic acid | -63.43 | -89.00 |
| 12-Oleanene-3,6,16,28-tetrol | -90.04 | -16.92 | 3,6,24-Trihydroxy-12-oleanen-28-oic acid, (3α,6α)-form | -89.86 | -97.97 |
| 12-Oleanene-3,6,16-triol, (3β,6β,16β)-form | -96.15 | -54.67 | 3,6,24-Trihydroxy-12-oleanen-28-oic acid, (3β,6α)-form | -90.28 | -97.98 |
| 12-Oleanene-3,6,16-triol | -100.24 | -51.72 | 3,6,24-Trihydroxy-12-oleanen-28-oic acid, (3β,6β)-form | -90.12 | -96.92 |
| 12-Oleanene-3,6,21-triol, (3β,6β,21β)-form | -96.60 | -97.89 | 3,6,24-Trihydroxy-12-oleanen-28-oic acid | -90.30 | -98.02 |
| 12-Oleanene-3,6,21-triol | -96.58 | -97.82 | 3,6,7-Trihydroxy-12-oleanen-27-oic acid, (3β,6β,7α)-form | -59.10 | -106.39 |
| 12-Oleanene-3,6,23,28-tetrol, (3β,6β)-form, 28-Carboxylic acid | -79.15 | -84.32 | 3,6,7-Trihydroxy-12-oleanen-27-oic acid | -55.14 | -105.81 |
| 12-Oleanene-3,6,23,28-tetrol, (3β,6β)-form | -71.14 | -69.46 | 3,6-Dihydroxy-12-oleanen-27-oic acid, (3α,6β)-form | -96.02 | -102.87 |
| 12-Oleanene-3,6,23,28-tetrol | -71.55 | -65.33 | 3,6-Dihydroxy-12-oleanen-27-oic acid, (3β,6α)-form | -51.76 | -85.16 |
| 12-Oleanene-3,6,7,16,23,28-hexol, (3β,6β,7β,16β)-form | -93.12 | -65.68 | 3,6-Dihydroxy-12-oleanen-27-oic acid, (3β,6β)-form | -49.35 | -85.09 |
| 12-Oleanene-3,6,7,16,23,28-hexol | -78.86 | -43.00 | 3,6-Dihydroxy-12-oleanen-27-oic acid, 3-Ac | -70.65 | -101.24 |
| 12-Oleanene-3,6-diol, (3β,6α)-form | -83.66 | -48.07 | 3,6-Dihydroxy-12-oleanen-27-oic acid | -48.66 | -85.13 |
| 12-Oleanene-3,6-diol, (3β,6β)-form | -83.43 | -48.05 | 3,6-Dihydroxy-12-oleanen-28-oic acid, (3α,6α)-form | -6.59 | -90.00 |
| 12-Oleanene-3,6-diol | -69.50 | -79.61 | 3,6-Dihydroxy-12-oleanen-28-oic acid, (3α,6β)-form | -7.14 | -90.16 |

**Supplementary Table 5 Cont’d:** Docking energies of *epi*-Oleanolic acid analogues for *L. infantum* and *T. cruzi* CYP51.

| **Compounds** | ***T. cruzi* CYP51** | ***L. infantum* CYP51** | **Compounds** | ***T. cruzi* CYP51** | ***L. infantum* CYP51** |
| --- | --- | --- | --- | --- | --- |
| 12-Oleanene-3,7,15,22,24-pentol, (3β,7β,15α,22β)-form | -88.15 | -100.68 | 3,6-Dihydroxy-12-oleanen-28-oic acid, (3β,6α)-form, 6-Ac | -61.88 | -94.23 |
| 12-Oleanene-3,7,15,22,24-pentol | -88.50 | -100.78 | 3,6-Dihydroxy-12-oleanen-28-oic acid, (3β,6α)-form | -88.40 | -92.24 |
| 12-Oleanene-3,7,22,24,29-pentol, (3β,7β,22β)-form | -102.56 | -102.66 | 3,6-Dihydroxy-12-oleanen-28-oic acid, (3β,6β)-form, 3-Ac, Me ester | -104.19 | -92.25 |
| 12-Oleanene-3,7,22,24,29-pentol | -103.02 | -107.80 | 3,6-Dihydroxy-12-oleanen-28-oic acid, (3β,6β)-form | -87.87 | -92.23 |
| 12-Oleanene-3,7-diol, (3β,7β)-form, 3-Hexadecanoyl | -136.87 | -116.30 | 3,6-Dihydroxy-12-oleanen-28-oic acid | -88.26 | -92.28 |
| 12-Oleanene-3,7-diol, (3β,7β)-form | -94.57 | -103.60 | 3,6-Dihydroxy-12-oleanen-29-oic acid, (3β,6β)-form | -100.73 | -28.80 |
| 12-Oleanene-3,7-diol | -82.16 | -98.51 | 3,6-Dihydroxy-12-oleanen-29-oic acid | -100.76 | -28.29 |
| 12-Oleanene-3,9,11-triol, (3β,9α,11α)-form, 3,11-Di-Ac | -49.34 | -74.36 | 3,6-Dihydroxy-12-oleanene-23,29-dioic acid, (3β,6β)-form | -37.05 | -79.22 |
| 12-Oleanene-3,9,11-triol, (3β,9α,11α)-form.sdf | -89.09 | -64.81 | 3,6-Dihydroxy-12-oleanene-23,29-dioic acid | -34.89 | -78.95 |
| 12-Oleanene-3,9,11-triol | -88.62 | -64.59 | 3,6-Dihydroxy-12-oleanene-27,28-dioic acid, (3β,6α)-form | -91.02 | -98.91 |
| 15,16-Epoxy-12-oleanen-3-ol, (3β,15α,16α)-form | -77.54 | -59.49 | 3,6-Dihydroxy-12-oleanene-27,28-dioic acid | -90.72 | -98.95 |
| 15,16-Epoxy-12-oleanen-3-ol | -87.52 | -74.34 | 3,7,21,23-Tetrahydroxy-12-oleanen-28-oic acid, (3β,7α,21β)-form | -82.27 | -73.81 |
| 15,16-Epoxy-12-oleanene-3,11,28-triol, (3β,11α,15α,16α)-form | -91.73 | -78.31 | 3,7,21,23-Tetrahydroxy-12-oleanen-28-oic acid | -82.04 | -86.25 |
| 15,16-Epoxy-12-oleanene-3,11,28-triol | -104.42 | -95.79 | 3,7,22-Trihydroxy-12-oleanen-28-oic acid, (3β,7β,22β)-form | -101.09 | -76.31 |
| 15,16-Epoxy-12-oleanene-3,28,30-triol, (3β,15α,16α)-form | -95.29 | -80.16 | 3,7,22-Trihydroxy-12-oleanen-28-oic acid | -101.69 | -75.42 |
| 15,16-Epoxy-12-oleanene-3,28,30-triol | -95.29 | -80.63 | 3,7-Dihydroxy-12-oleanen-28-oic acid, (3β,7α)-form | -106.11 | -110.41 |
| 15,27-Cyclo-3,21-dihydroxy-12-oleanen-28-oic acid, (3β,21β)-form | -68.10 | -76.57 | 3,7-Dihydroxy-12-oleanen-28-oic acid, (3β,7β)-form | -93.35 | -70.45 |
| 15,27-Cyclo-3,21-dihydroxy-12-oleanen-28-oic acid | -101.52 | -77.22 | 3,7-Dihydroxy-12-oleanen-28-oic acid | -105.56 | -110.64 |

**Supplementary Table 5 Cont’d:** Docking energies of *epi*-Oleanolic acid analogues for *L. infantum* and *T. cruzi* CYP51.

| **Compounds** | ***T. cruzi* CYP51** | ***L. infantum* CYP51** | **Compounds** | ***T. cruzi* CYP51** | ***L. infantum* CYP51** |
| --- | --- | --- | --- | --- | --- |
| 16,21-Epoxy-12-oleanene-3,15,22,28,30-pentol, (3β,15α,16α,21α,22α)-form | -92.44 | -86.17 | 3-(Octahydro-4,7-dimethyl-1-oxocyclopenta[c]pyran-3-yloxy)-12-oleanen-28-oic acid | -134.35 | -92.92 |
| 16,21-Epoxy-12-oleanene-3,15,22,28,30-pentol | -67.07 | -60.07 | 3-Hydroxy-11-oxo-12-oleanen-28-oic acid, 3α-form | -92.91 | -91.89 |
| 16,28-Epoxy-12-oleanen-3-ol, (3β,16β)-form | -73.93 | -65.48 | 3-Hydroxy-11-oxo-12-oleanen-28-oic acid, 3β-form, 3-Octanoyl | -111.28 | -121.38 |
| 16,28-Epoxy-12-oleanen-3-ol | -73.97 | -65.35 | 3-Hydroxy-11-oxo-12-oleanen-28-oic acid, 3β-form, Ac | -75.09 | -69.55 |
| 18-Hydroperoxy-3-hydroxy-12-oleanen-11-one, (3β,18α)-form, 3-Ac | -78.00 | -60.75 | 3-Hydroxy-11-oxo-12-oleanen-28-oic acid, 3β-form | -84.49 | -87.81 |
| 18-Hydroperoxy-3-hydroxy-12-oleanen-11-one, (3β,18α)-form | -94.98 | -95.63 | 3-Hydroxy-11-oxo-12-oleanen-28-oic acid | -84.59 | -88.05 |
| 18-Hydroperoxy-3-hydroxy-12-oleanen-11-one | -94.99 | -95.67 | 3-Hydroxy-11-oxo-12-oleanen-29-oic acid, 3β-form, Ac, Me ester | -92.36 | -100.81 |
| 2,3,11,23-Tetrahydroxy-12-oleanene-28,30-dioic acid, (2β,3β,11α)-form, 23-Aldehyde, 30-Me ester | -52.47 | -105.34 | 3-Hydroxy-11-oxo-12-oleanen-29-oic acid, 3β-form, Ac | -103.99 | -82.98 |
| 2,3,11,23-Tetrahydroxy-12-oleanene-28,30-dioic acid, (2β,3β,11α)-form | -116.80 | -34.47 | -Hydroxy-11-oxo-12-oleanen-29-oic acid, 3β-form, Me ester | -95.00 | -40.56 |
| 2,3,11,23-Tetrahydroxy-12-oleanene-28,30-dioic acid, (2β,3β,11β)-form | -89.26 | -93.56 | 3-Hydroxy-11-oxo-12-oleanen-29-oic acid, 3β-form | -93.67 | -75.23 |
| 2,3,11,23-Tetrahydroxy-12-oleanene-28,30-dioic acid | -110.04 | -102.54 | 3-Hydroxy-11-oxo-12-oleanen-29-oic acid | -90.64 | -43.80 |
| 2,3,15,21-Tetrahydroxy-12-oleanen-28-oic acid, (2β,3β,15α,21β)-form | -88.61 | -83.29 | 3-Hydroxy-11-oxo-12-oleanen-30-oic acid, (3β,18α)-form | -91.42 | -43.18 |
| 2,3,15,21-Tetrahydroxy-12-oleanen-28-oic acid | -93.89 | -80.34 | 3-Hydroxy-11-oxo-12-oleanen-30-oic acid, (3β,18β)-form, 3-Ac, 3-phenyl-2-propenyl ester | -54.52 | -122.85 |
| 2,3,15-Trihydroxy-12-oleanene-23,28-dioic acid, (2α,3β)-form | -101.25 | -44.00 | 3-Hydroxy-11-oxo-12-oleanen-30-oic acid, (3β,18β)-form, 3-Ac | -46.31 | -90.90 |
| 2,3,15-Trihydroxy-12-oleanene-23,28-dioic acid | -101.59 | -44.91 | 3-Hydroxy-11-oxo-12-oleanen-30-oic acid, (3β,18β)-form, 3-O-(3-Carboxypropanoyl) | -46.41 | -117.82 |
| 2,3,16,21,23-Pentahydroxy-12-oleanen-28-oic acid, (2β,3β,16α,21β)-form | -104.08 | -111.62 | 3-Hydroxy-11-oxo-12-oleanen-30-oic acid, (3β,18β)-form, 30-Alcohol | -91.82 | -104.05 |

**Supplementary Table 5 Cont’d:** Docking energies of *epi*-Oleanolic acid analogues for *L. infantum* and *T. cruzi* CYP51.

| **Compounds** | ***T. cruzi* CYP51** | ***L. infantum* CYP51** | **Compounds** | ***T. cruzi* CYP51** | ***L. infantum* CYP51** |
| --- | --- | --- | --- | --- | --- |
| 2,3,16,21,23-Pentahydroxy-12-oleanen-28-oic acid, (2β,3β,16β,21β)-form, 28→21 Lactone | -89.31 | -88.50 | 3-Hydroxy-11-oxo-12-oleanen-30-oic acid, (3β,18β)-form, Me ester | -95.17 | -104.93 |
| 2,3,16,21,23-Pentahydroxy-12-oleanen-28-oic acid, (2β,3β,16β,21β)-form | -98.66 | -111.95 | 3-Hydroxy-11-oxo-12-oleanen-30-oic acid, (3β,18β)-form | -54.88 | -100.47 |
| 2,3,16,21,23-Pentahydroxy-12-oleanen-28-oic acid | -105.66 | -112.01 | 3-Hydroxy-11-oxo-12-oleanen-30-oic acid | -93.70 | -8.97 |
| 2,3,16,21-Tetrahydroxy-12-oleanen-28-oic acid, (2β,3β,16β,21β)-form | -91.57 | -65.02 | 3-Hydroxy-12-oleanen-27-oic acid, (3β,18ξ)-form, Ac | -39.34 | -107.06 |
| 2,3,16,21-Tetrahydroxy-12-oleanen-28-oic acid | -91.22 | -23.23 | 3-Hydroxy-12-oleanen-27-oic acid, (3β,18ξ)-form | -99.38 | -97.66 |
| 2,3,16,21-Tetrahydroxy-12-oleanene-23,28-dioic acid, 28→21 Lactone | -100.04 | -97.88 | 3-Hydroxy-12-oleanen-27-oic acid, 3α-form, 3-O-(3,4-Dihydroxy-E-cinnamoyl) | -40.66 | -93.86 |
| 2,3,16,21-Tetrahydroxy-12-oleanene-23,28-dioic acid | -88.43 | -89.59 | 3-Hydroxy-12-oleanen-27-oic acid, 3α-form | -106.53 | -98.78 |
| 2,3,16,21-Tetrahydroxy-12-oleanene-24,28-dioic acid, (2β,3β,16β,21β)-form | -97.09 | -96.52 | 3-Hydroxy-12-oleanen-27-oic acid, 3β-form, Ac | -91.81 | -98.10 |
| 2,3,16,21-Tetrahydroxy-12-oleanene-24,28-dioic acid | -95.95 | -61.27 | 3-Hydroxy-12-oleanen-27-oic acid, 3β-form | -99.32 | -97.89 |
| 2,3,16,23,24-Pentahydroxy-12-oleanen-28-oic acid, (2β,3β,16α)-form | -68.99 | -92.84 | 3-Hydroxy-12-oleanen-27-oic acid | -99.29 | -98.88 |
| 2,3,16,23,24-Pentahydroxy-12-oleanen-28-oic acid | -71.50 | -99.30 | 3-Hydroxy-12-oleanen-28-oic acid, 3α-form | -99.97 | -13.62 |
| 2,3,16,23-Tetrahydroxy-12-oleanen-28-oic acid, (2β,3β,16α)-form, 16-Ketone | -104.61 | -96.91 | 3-Hydroxy-12-oleanen-28-oic acid, 3β-form, (3β,9β)-form | -86.81 | -96.72 |
| 2,3,16,23-Tetrahydroxy-12-oleanen-28-oic acid, (2β,3β,16α)-form | -87.61 | -87.58 | 3-Hydroxy-12-oleanen-28-oic acid, 3β-form, 3-O-(4-Hydroxy-Z-cinnamoyl) | -120.07 | -133.27 |
| 2,3,16,23-Tetrahydroxy-12-oleanen-28-oic acid | -91.02 | -87.25 | 3-Hydroxy-12-oleanen-28-oic acid, 3β-form, 3-Pentadecanoyl | -116.65 | -103.35 |
| 2,3,16,23-Tetrahydroxy-12-oleanene-24,28-dioic acid, (2β,3β,16α)-form | -86.28 | -82.12 | 3-Hydroxy-12-oleanen-28-oic acid, 3β-form, 3-Tetradecanoyl | -70.88 | -124.76 |
| 2,3,16,23-Tetrahydroxy-12-oleanene-24,28-dioic acid | -108.90 | -113.27 | 3-Hydroxy-12-oleanen-28-oic acid, 3β-form, Ac, Me ester | -99.63 | -68.57 |
| 2,3,16,24,30-Pentahydroxy-12-oleanen-28-oic acid, (2ξ,3ξ,16α)-form | -109.50 | -54.60 | 3-Hydroxy-12-oleanen-28-oic acid, 3β-form, Ac | -68.60 | -99.37 |

**Supplementary Table 5 Cont’d:** Docking energies of *epi*-Oleanolic acid analogues for *L. infantum* and *T. cruzi* CYP51.

| **Compounds** | ***T. cruzi* CYP51** | ***L. infantum* CYP51** | **Compounds** | ***T. cruzi* CYP51** | ***L. infantum* CYP51** |
| --- | --- | --- | --- | --- | --- |
| 2,3,16,24,30-Pentahydroxy-12-oleanen-28-oic acid | -105.58 | -55.53 | 3-Hydroxy-12-oleanen-28-oic acid, 3β-form,3-O-(4-Methoxy-Z-cinnamoyl), Me ester | -83.72 | -142.66 |
| 2,3,16-Trihydroxy-12-oleanen-28-oic acid, (2β,3β,16α)-form | -92.67 | -69.67 | 3-Hydroxy-12-oleanen-28-oic acid, 3β-form | -99.99 | -14.54 |
| 2,3,16-Trihydroxy-12-oleanen-28-oic acid | -92.67 | -69.60 | 3-Hydroxy-12-oleanen-28-oic acid | -99.99 | -35.41 |
| 2,3,16-Trihydroxy-12-oleanene-23,28-dioic acid, (2β,3β,16α)-form, 23-Me ester | -98.17 | -87.79 | 3-Hydroxy-12-oleanen-28-oic acid, 3β-form, 28-(4-Formylphenyl) ester | -108.10 | -94.11 |
| 2,3,16-Trihydroxy-12-oleanene-23,28-dioic acid, (2β,3β,16α)-form | -104.31 | -76.52 | 3-Hydroxy-12-oleanen-28-oic acid, 3β-form, 3-(2-Hydroxyethyl) ether | -76.60 | -106.42 |
| 2,3,16-Trihydroxy-12-oleanene-23,28-dioic acid | -104.62 | -76.35 | 3-Hydroxy-12-oleanen-28-oic acid, 3β-form, 3-Ac, 28-nonyl ester | -113.42 | -124.13 |
| 2,3,18-Trihydroxy-12-oleanen-28-oic acid, (2α,3β,18β)-form | -34.39 | -79.91 | 3-Hydroxy-12-oleanen-28-oic acid, 3β-form, 3-Hexadecanoyl | -126.08 | -120.34 |
| 2,3,18-Trihydroxy-12-oleanen-28-oic acid | -79.55 | -79.81 | 3-Hydroxy-12-oleanen-28-oic acid, 3β-form, 3-O-(3,4-Dihydroxycinnamoyl) | -122.22 | -139.55 |
| 2,3,18-Trihydroxy-12-oleanene-23,28-dioic acid, (2α,3β)-form, Di-Ac, di-Me ester | -75.12 | -76.99 | 3-Hydroxy-12-oleanen-28-oic acid, 3β-form, 3-O-(3-Carboxypropanoyl) | -82.11 | -109.39 |
| 2,3,18-Trihydroxy-12-oleanene-23,28-dioic acid, (2α,3β)-form, Di-Ac | -71.44 | -100.81 | 3-Hydroxy-12-oleanen-28-oic acid, 3β-form, 3-O-(4-Hydroxy-3-methoxy-E-cinnamoyl) | -80.71 | -116.28 |
| 2,3,18-Trihydroxy-12-oleanene-23,28-dioic acid, (2α,3β)-form | -95.40 | -103.15 | 3-Hydroxy-12-oleanen-28-oic acid, 3β-form, 3-O-(4-Hydroxy-E-cinnamoyl) | -120.15 | -129.29 |
| 2,3,18-Trihydroxy-12-oleanene-23,28-dioic acid | -95.26 | -103.17 | 3-Hydroxy-12-oleanen-29-oic acid, 3α-form, 3-Ac | -44.19 | -91.51 |
| 2,3,19,23,24-Pentahydroxy-11-oxo-12-oleanen-28-oic acid, (2α,3β,19α)-form | -73.28 | -55.24 | 3-Hydroxy-12-oleanen-29-oic acid, 3α-form, 3-Benzoyl, Me ester | -84.01 | -64.50 |
| 2,3,19,23,24-Pentahydroxy-11-oxo-12-oleanen-28-oic acid, (2α,3β,19β)-form | -72.70 | -74.32 | 3-Hydroxy-12-oleanen-29-oic acid, 3α-form | -85.55 | -65.78 |
| 2,3,19,23,24-Pentahydroxy-11-oxo-12-oleanen-28-oic acid | -78.78 | -98.49 | 3-Hydroxy-12-oleanen-29-oic acid, 3β-form | -79.56 | -45.26 |
| 2,3,19,23,24-Pentahydroxy-12-oleanen-28-oic acid, (2α,3α,19α)-form | -95.05 | -88.19 | 3-Hydroxy-12-oleanen-29-oic acid | -86.94 | -64.99 |
| 2,3,19,23,24-Pentahydroxy-12-oleanen-28-oic acid, (2α,3β,19α)-form, 23-Carboxylic acid | -105.24 | -79.23 | 3-Hydroxy-12-oleanen-30-oic acid, 3α-form, 3-O-(3,4-Dihydroxy-E-cinnamoyl) (**3-*O*-caffeoyl-20-Epikatonic acid**) | -138.32 | -143.99 |
| 2,3,19,23,24-Pentahydroxy-12-oleanen-28-oic acid, (2α,3β,19α)-form, 24-Carboxylic acid | -90.68 | -11.32 | 3-Hydroxy-12-oleanen-30-oic acid, 3α-form, 3-O-(4-Hydroxy-E-cinnamoyl) | -127.36 | -134.33 |

**Supplementary Table 5 Cont’d:** Docking energies of *epi*-Oleanolic acid analogues for *L. infantum* and *T. cruzi* CYP51.

| **Compounds** | ***T. cruzi* CYP51** | ***L. infantum* CYP51** | **Compounds** | ***T. cruzi* CYP51** | ***L. infantum* CYP51** |
| --- | --- | --- | --- | --- | --- |
| 2,3,19,23,24-Pentahydroxy-12-oleanen-28-oic acid, (2α,3β,19α)-form | -96.88 | -76.20 | 3-Hydroxy-12-oleanen-30-oic acid, 3α-form | -43.26 | -89.34 |
| 2,3,19,23,24-Pentahydroxy-12-oleanen-28-oic acid | -94.87 | -30.29 | 3-Hydroxy-12-oleanen-30-oic acid, 3β-form, 3-Ac | -80.92 | -88.00 |
| 2,3,19,23-Tetrahydroxy-11-oxo-12-oleanen-28-oic acid, (2α,3β,19α)-form | -100.76 | -100.43 | 3-Hydroxy-12-oleanen-30-oic acid, 3β-form | -46.13 | -89.37 |
| 2,3,19,23-Tetrahydroxy-11-oxo-12-oleanen-28-oic acid | -66.71 | -77.08 | 3-Hydroxy-12-oleanen-30-oic acid | -88.52 | -89.64 |
| 2,3,19,23-Tetrahydroxy-12-oleanen-28-oic acid, (2α,3α,19α)-form | -97.88 | -88.27 | 3-Hydroxy-12-oleanene-23,28-dioic acid, 3α-form | -90.40 | -89.96 |
| 2,3,19,23-Tetrahydroxy-12-oleanen-28-oic acid, (2α,3β,19α)-form, 2,3,23-Tri-Ac | -84.70 | -102.18 | 3-Hydroxy-12-oleanene-23,28-dioic acid, 3β-form, 3-Ac | -59.81 | -106.14 |
| 2,3,19,23-Tetrahydroxy-12-oleanen-28-oic acid, (2α,3β,19α)-form | -75.54 | -85.70 | 3-Hydroxy-12-oleanene-23,28-dioic acid, 3β-form | -89.42 | -89.50 |
| 2,3,19,23-Tetrahydroxy-12-oleanen-28-oic acid, (2α,3β,19β)-form | -95.12 | -109.98 | 3-Hydroxy-12-oleanene-23,28-dioic acid | -90.54 | -91.24 |
| 2,3,19,23-Tetrahydroxy-12-oleanen-28-oic acid, (2β,3β,19α)-form | -85.62 | -109.41 | 3-Hydroxy-12-oleanene-27,28-dioic acid, (3β,18α)-form | -85.00 | -96.01 |
| 2,3,19,23-Tetrahydroxy-12-oleanen-28-oic acid | -97.70 | -88.25 | 3-Hydroxy-12-oleanene-27,28-dioic acid, 3β-form | 117.65 | 95.37 |
| 2,3,19,24-Tetrahydroxy-11-oxo-12-oleanen-28-oic acid, (2α,3β,19β)-form | -122.06 | -20.33 | 3-Hydroxy-12-oleanene-27,28-dioic acid | 117.40 | 94.99 |
| 2,3,19,24-Tetrahydroxy-11-oxo-12-oleanen-28-oic acid | -95.47 | -55.65 | 3-Hydroxy-12-oleanene-28,29-dioic acid, 3α-form, 3-Ac | -106.84 | -111.46 |
| 2,3,19,24-Tetrahydroxy-12-oleanen-28-oic acid, (2α,3α,19α)-form | -98.00 | -95.44 | 3-Hydroxy-12-oleanene-28,29-dioic acid, 3α-form | -83.57 | -107.97 |
| 2,3,19,24-Tetrahydroxy-12-oleanen-28-oic acid, (2α,3β,19α)-form, 2,3,23-Tri-Ac | -84.58 | -121.77 | 3-Hydroxy-12-oleanene-28,29-dioic acid, 3β-form, 28-Me ester | -104.62 | -66.00 |
| 2,3,19,24-Tetrahydroxy-12-oleanen-28-oic acid, (2α,3β,19α)-form | -91.57 | -102.64 | 3-Hydroxy-12-oleanene-28,29-dioic acid, 3β-form, 29-Et ester | -102.22 | -80.92 |
| 2,3,19,24-Tetrahydroxy-12-oleanen-28-oic acid | -91.80 | -102.72 | 3-Hydroxy-12-oleanene-28,29-dioic acid, 3β-form, 3-Ac, di-Me ester | -69.17 | -120.00 |
| 2,3,19,24-Tetrahydroxy-12-oleanen-30-oic acid, (2α,3β,19α)-form | -93.97 | -62.26 | 3-Hydroxy-12-oleanene-28,29-dioic acid, 3β-form, 3-Ac | -84.14 | -103.85 |

**Supplementary Table 5 Cont’d:** Docking energies of *epi*-Oleanolic acid analogues for *L. infantum* and *T. cruzi* CYP51.

| **Compounds** | ***T. cruzi* CYP51** | ***L. infantum* CYP51** | **Compounds** | ***T. cruzi* CYP51** | ***L. infantum* CYP51** |
| --- | --- | --- | --- | --- | --- |
| 2,3,19,24-Tetrahydroxy-12-oleanen-30-oic acid | -93.75 | -74.57 | 3-Hydroxy-12-oleanene-28,29-dioic acid, 3β-form, Di-Me ester | -84.76 | -91.56 |
| 2,3,19-Trihydroxy-11-oxo-12-oleanen-28-oic acid, (2α,3β,19α)-form | -94.15 | -97.96 | 3-Hydroxy-12-oleanene-28,29-dioic acid, 3β-form | -97.97 | -104.07 |
| 2,3,19-Trihydroxy-11-oxo-12-oleanen-28-oic acid, (2α,3β,19β)-form | -94.46 | -97.52 | 3-Hydroxy-12-oleanene-28,29-dioic acid | -83.39 | -107.75 |
| 2,3,19-Trihydroxy-11-oxo-12-oleanen-28-oic acid | -94.56 | -97.66 | -Hydroxy-12-oleanene-28,30-dioic acid, 3β-form, 3-Ac, 30-Me ester | -97.76 | -79.20 |
| 2,3,19-Trihydroxy-12-oleanen-28-oic acid, (2α,3α,19α)-form | -93.24 | -85.87 | 3-Hydroxy-12-oleanene-28,30-dioic acid, 3β-form, 30-Me ester | -101.22 | -105.44 |
| 2,3,19-Trihydroxy-12-oleanen-28-oic acid, (2α,3β,19α)-form | -52.96 | -78.26 | 3-Hydroxy-12-oleanene-28,30-dioic acid, 3β-form, Di-Me ester | -25.78 | -101.94 |
| 2,3,19-Trihydroxy-12-oleanen-28-oic acid, (2α,3β,19β)-form | -88.32 | -100.54 | 3-Hydroxy-12-oleanene-28,30-dioic acid, 3β-form | -68.70 | -107.27 |
| 2,3,19-Trihydroxy-12-oleanen-28-oic acid | -53.21 | -80.48 | 3-Hydroxy-12-oleanene-28,30-dioic acid | -69.45 | -107.43 |
| 2,3,19-Trihydroxy-12-oleanen-29-oic acid, (2α,3β,19α)-form, Me ester | -91.37 | -70.66 | 3-Hydroxy-2-oxo-12-oleanen-30-oic acid, 3β-form | -70.69 | -86.53 |
| 2,3,19-Trihydroxy-12-oleanen-29-oic acid, (2α,3β,19α)-form | -88.80 | -82.22 | 3-Hydroxy-2-oxo-12-oleanen-30-oic acid | -73.91 | -86.13 |
| 2,3,19-Trihydroxy-12-oleanen-29-oic acid | -88.82 | -82.23 | 3-Hydroxy-23-oxo-12-oleanen-28-oic acid, 3β-form | -82.83 | -88.73 |
| 2,3,19-Trihydroxy-12-oleanene-23,28-dioic acid, (2α,3β,19α)-form | -103.09 | -102.78 | 3-Hydroxy-23-oxo-12-oleanen-28-oic acid | -100.95 | -86.31 |
| 2,3,19-Trihydroxy-12-oleanene-23,28-dioic acid, (2α,3β,19β)-form | -99.64 | -37.48 | 3,6-Dihydroxy-12-oleanene-27,28-dioic acid, (6β)-form, 28-Carboxylic acid, 3β-alcohol | -81.56 | -103.17 |
| 2,3,19-Trihydroxy-12-oleanene-23,28-dioic acid | -102.40 | -75.19 | 9,25-Cyclo-12-oleanen-3-ol, 3β-form | -100.32 | -90.20 |
| 2,3,19-Trihydroxy-12-oleanene-24,28-dioic acid, (2α,3β,19α)-form | -0.56 | 77.48 | 9,25-Cyclo-12-oleanen-3-ol | -100.33 | -88.88 |
| 2,3,19-Trihydroxy-12-oleanene-24,28-dioic acid, (2α,3β,19β)-form | 1.58 | 27.07 | Acacigenin B, 16-Deoxy, Me ester | -75.32 | -131.99 |
| 2,3,19-Trihydroxy-12-oleanene-24,28-dioic acid | 4.33 | 27.42 | Acacigenin B, 16-Deoxy | -81.48 | -104.83 |
| 2,3,21,28-Tetrahydroxy-12-oleanen-27-oic acid, (2β,3β,21β)-form | -22.19 | -43.91 | Acacigenin B | -8.04 | -97.34 |
| 2,3,21,28-Tetrahydroxy-12-oleanen-27-oic acid | -77.86 | -44.24 | Anemoclemoside A | -113.81 | -70.39 |

**Supplementary Table 5 Cont’d:** Docking energies of *epi*-Oleanolic acid analogues for *L. infantum* and *T. cruzi* CYP51.

| **Compounds** | ***T. cruzi* CYP51** | ***L. infantum* CYP51** | **Compounds** | ***T. cruzi* CYP51** | ***L. infantum* CYP51** |
| --- | --- | --- | --- | --- | --- |
| 2,3,22,27-Tetrahydroxy-12-oleanene-23,28-dioic acid, (2β,3β,22β)-form | -92.61 | -54.24 | Chilianthin A, 7′,8′-Diepimer | 26.59 | -101.49 |
| 2,3,22,27-Tetrahydroxy-12-oleanene-23,28-dioic acid | -95.02 | -52.84 | Chilianthin A | -50.20 | -79.85 |
| 2,3,22-Trihydroxy-12-oleanen-29-oic acid, (2α,3β,22α)-form, 29→22 Lactone | -73.59 | -78.10 | Chilianthin C | 42.88 | -16.25 |
| 2,3,22-Trihydroxy-12-oleanen-29-oic acid, (2α,3β,22α)-form | -48.61 | -48.80 | Chilianthin E | 149.64 | -90.23 |
| 2,3,22-Trihydroxy-12-oleanen-29-oic acid | -48.57 | -52.30 | Cyclamiretin C, 30-Me ether | -73.00 | -70.36 |
| 2,3,23,24-Tetrahydroxy-12-oleanen-28-oic acid, (2α,3β)-form | -96.47 | -90.99 | Cyclamiretin C, O30-Et (30α-) | -18.08 | -55.04 |
| 2,3,23,24-Tetrahydroxy-12-oleanen-28-oic acid | -61.75 | -88.21 | Cyclamiretin C, O30-Et (30β-) | -17.11 | -93.14 |
| 2,3,23,27-Tetrahydroxy-12-oleanen-28-oic acid, (2α,3β)-form | -91.36 | -113.87 | Cyclamiretin C, O30-Me | -97.20 | -80.76 |
| 2,3,23,27-Tetrahydroxy-12-oleanen-28-oic acid | -98.87 | -93.67 | Cyclamiretin C | -52.65 | -62.51 |
| 2,3,23,29-Tetrahydroxy-12-oleanen-28-oic acid, (2α,3α)-form, 3,23-O-Isopropylidene | -48.79 | -91.02 | Escigenin, 24-Deoxy | -56.87 | -58.18 |
| 2,3,23,29-Tetrahydroxy-12-oleanen-28-oic acid, (2α,3α)-form | -63.95 | -94.49 | Escigenin | -64.65 | -50.04 |
| 2,3,23,29-Tetrahydroxy-12-oleanen-28-oic acid, (2α,3β)-form, 3,23-Disulfate | -92.28 | -115.46 | Eupatoric acid | -91.71 | -52.04 |
| 2,3,23,29-Tetrahydroxy-12-oleanen-28-oic acid, (2α,3β)-form | -62.91 | -89.76 | Liquoric acid | -69.98 | -55.40 |
| 2,3,23,29-Tetrahydroxy-12-oleanen-28-oic acid | -67.83 | -92.14 | Monacanthic acid | -89.70 | -28.58 |
| 2,3,23,30-Tetrahydroxy-12-oleanen-28-oic acid, (2α,3α)-form | -77.92 | -87.61 | Officigenin | -100.46 | -116.98 |
| 2,3,23,30-Tetrahydroxy-12-oleanen-28-oic acid, (2α,3β)-form | -80.25 | -90.35 | Reglin | -101.84 | -78.94 |
| 2,3,23,30-Tetrahydroxy-12-oleanen-28-oic acid, (2β,3β)-form, 30-Aldehyde | -97.78 | -65.10 | Sanguidiogenin E | -8.38 | -60.60 |
| 2,3,23,30-Tetrahydroxy-12-oleanen-28-oic acid, (2β,3β)-form | -84.15 | -90.59 | Sanguidioside D | -16.90 | -10.05 |
| 2,3,23,30-Tetrahydroxy-12-oleanen-28-oic acid | -84.15 | -90.65 | Uragogin | -128.98 | -94.36 |
| 2,3,23-Trihydroxy-11-oxo-12-oleanen-28-oic acid, (2α,3β)-form | -100.14 | -107.96 |  |  |  |

**Supplementary Table 6:** Docking energies of CYP51 inhibitors.

| **CYP51 inhibitors** | **Docking energies (re-rank scores)** | | | | |
| --- | --- | --- | --- | --- | --- |
|  | *T. cruzi* | *T. brucei* | *M. Tuberculosis* | *L. infantum* | *H. sapiens* |
| ketoconazole | -129.83 | -122.15 | -103.20 | -148.25 | -129.36 |
| N-1-(2,4-dichlorophenyl)-2-(1H-imidazol-1-yl)ethyl)-4-(5-phenyl-1,3,4-oxadi-azol-2-yl)benzamide | -128.54 | -134.46 | -121.65 | -128.63 | -125.72 |
